# IL-10 dependent adaptation allows macrophages to adjust inflammatory responses to TLR4 stimulation history

**DOI:** 10.1101/2024.03.28.587272

**Published:** 2024-03-31

**Authors:** H. Bongartz, C. Bradfield, J. Gross, I.D.C. Fraser, A. Nita-Lazar, M. Meier-Schellersheim

**Affiliations:** 1 Computational Systems Biology Section, Laboratory of Immune System Biology, National Institute of Allergy and infectious Diseases, National Institutes of Health, Bethesda, MD, USA; 2 Signaling Systems Section, Laboratory of Immune System Biology, National Institute of Allergy and infectious Diseases, National Institutes of Health, Bethesda, MD, USA; 3 Functional Cellular Networks Section, Laboratory of Immune System Biology, National Institute of Allergy and infectious Diseases, National Institutes of Health, Bethesda, MD, USA

**Keywords:** innate immunity, macrophages, pattern recognition receptor (PRR), toll-like receptor 4 (TLR4), IL-10, temporal gradients, adaptation, inflammation

## Abstract

During an infection, innate immune cells must adjust nature and strength of their responses to changing pathogen abundances. To determine how stimulation of the pathogen sensing TLR4 shapes subsequent macrophage responses, we systematically varied priming and restimulation concentrations of its ligand KLA. We find that different priming strengths have very distinct effects at multiple stages of the signaling response, including receptor internalization, MAPK activation, cytokine and chemokine production, and nuclear translocation and chromatin association of NFκB and IκB members. In particular, restimulation-induced TNF-α production required KLA doses equal to or greater than those used for prior exposure, indicating that macrophages can detect and adaptively respond to changing TLR4 stimuli. Interestingly, while such adaptation was dependent on the anti-inflammatory cytokine IL-10, exogenous concentrations of IL-10 corresponding to those secreted after strong priming did not exert suppressive effects on TNF-α without such prior priming, confirming the critical role of TLR4 stimulation history.

## Introduction

Macrophages, together with other myeloid cells of the innate immune system, form the primary defense against many pathogens. Pattern recognition receptors (PRRs) such as toll-like receptors (TLR), expressed mainly by macrophages and other myeloid cells, sense pathogen associated molecular patterns (PAMPS). Among these receptors, TLR4 recognizes lipopolysaccharides (LPS) expressed on the cell surface of gram-negative bacteria (for review, see [1]). Its ligation leads to the activation of multiple intracellular signaling cascades such as the Myeloid differentiation primary response 88 (MyD88)-dependent mitogen-activated protein kinase (MAPK), nuclear factor κB (NFκB) and the Toll/IL-1R domain-containing adaptor-inducing IFN-β (TRIF)-dependent Interferon regulatory factor (IRF) pathways, culminating in target gene expression of pro- and anti-inflammatory cytokines (for review, see [2]).

Importantly, macrophages and other cells of the innate immune system must carefully adapt the production of pro- and anti-inflammatory cytokines to the strength and temporal evolution of the pathogenic challenge at hand to ensure efficient control over the course of an infection while minimizing damage to host tissues caused by overly strong inflammatory signals. Reflecting this need for adaptive regulation, innate immune cells have been found to become sensitized by low dose pathogen exposure, showing increased responses while, on the other hand, developing tolerance and becoming hypo-responsive upon prolonged and repeated endotoxin exposure [3, 4]. Such dynamics have been shown in macrophages following long-term and repeated stimulation of TLR4, a major player in pathological inflammation and sepsis [5-9].

We previously reported the existence of a threshold for TLR4 stimulation below which bone marrow derived macrophages (BMDM) did not respond by producing inflammatory cytokines [10]. Importantly, the dose dependence of the activation of inflammatory responses was aligned with the threshold-like response of MAPK phosphorylation, suggesting a gatekeeping role for MAP kinases.

To assess the potential involvement of MAP kinases in TLR4 adaptation and, more broadly, explore the mechanisms that control the balance of pro- and anti-inflammatory responses, we subjected BMDM to repeated TLR4 stimuli whose strengths were systematically varied. Analyzing several layers of regulation including TLR4 internalization, MAPK phosphorylation, NFκB activation and nuclear translocation, as well as mRNA and protein level dynamics of pro- and anti-inflammatory cytokines, we found a surprising degree of differential adaptation for these components and a dependence on IL-10 signaling that, itself, was determined by the history of TLR4 stimulation.

A variety of molecular mechanisms associated with hypo-responsiveness following prolonged TLR4 stimulation have been described [7, 11-16]. Interleukin-10 (IL-10), in particular, has been identified as an important signal mediating the downregulation of pro-inflammatory cytokines [17, 18] and playing a central role for the adaptation of macrophages [19]. Among the signaling components that are involved in adaptation and that have previously been reported to be modulated by IL-10 are, for instance, Suppressor of cytokine signaling proteins (SOCS), Interleukin-1 receptor-associated kinase M (IRAK-M), as well as several microRNAs [20-27]. It has, however, remained unclear how their modulation depends on the TLR4 activation history and, thus, how this history shapes their influence on the regulation of pro- and anti-inflammatory cytokines, in part due to inconsistent strengths and durations of TLR4 stimulations used across those studies. Whereas, for example, some studies found Suppressor of cytokine signaling 3 (SOCS-3) to be highly induced by IL-10 signaling and responsible for downregulation of pro-inflammatory cytokines, such as tumor necrosis factor alpha (TNF-α) and Interleukin-6 (IL-6) [20, 25] another study found that SOCS-3 was not able to interfere with TNF-α expression [28].

B-cell lymphoma 3-encoded protein (BCL-3), an IκB family member, is another molecule that has been reported to mediate some of the anti-inflammatory effects of IL-10 [29-32]. BCL-3 can translocate to the nucleus to attenuate DNA binding of the NFκB component p65 (also known as RelA) and thereby suppress TNF-α production [30, 32-34]. It was shown that IL-10 induces expression of BCL-3 in macrophages in a STAT3 dependent manner [32]. However, again, the dependence of BCL-3 induction on the quantitative characteristics of TLR4 stimuli, that is, whether pathogen-related signals increase or decrease over time, had not been systematically studied.

Reporting the first systematic study of the dependence of macrophage responsiveness on the history of TLR4 stimulation, we analyze in detail how priming cells with low concentrations of the TLR4 ligand KLA induces stronger responses in a subset of signaling components whereas priming with high KLA concentrations mostly leads to hypo-responsiveness upon restimulation.

For the premier inflammatory cytokine TNF-α we find a particularly interesting dependence on the TLR4 stimulation history: only restimulation with KLA doses greater than those used for priming leads to strong responses, indicating that macrophages can detect and respond to the temporal evolution of the pathogen load detected by TLR4.

Whereas TNF-α and IL-6, another cytokine associated with inflammation, showed a pattern of low-dose sensitization and high-dose adaptation, other components, among them, importantly, the anti-inflammatory cytokines IFN-β and IL-10, showed more complex dose-dependent behaviors with non-monotonous responses that featured ranges of minimal production after medium-strength priming with KLA. Many of these characteristics were strongly altered when IL-10 signals were blocked.

Further, we find that neither TLR4 endocytosis nor adaptation at the level of MAPK activation preclude the ability of BMDM to respond to KLA restimulation, but that the desensitization we observe is strongly controlled by IL-10 as blocking its receptor IL-10R1 completely abrogated adaptation of several inflammatory responses.

Surprisingly, the suppressive effect of IL-10 on TNF-α production turned out be dependent on prior stimulation with high KLA doses. Low and mid-level priming followed by application of exogenous IL-10 at concentrations matching those produced in response to strong priming did not lead to reduced TNF-α production upon restimulation. This suggests that the TLR4 stimulation history determines the ability of IL-10 to act as a negative regulator of inflammation.

## Materials and Methods

### Materials

Kdo2-Lipid A (KLA) was purchased from Avanti Polar Lipids. Mek inhibitor U0126 (V1121) was obtained from Promega and re-constituted in DMSO (Sigma-Aldrich, D8418). The Alexa Fluor^™^ 647-conjugated threonine 202- and tyrosine 204-phosphorylated Erk1/2 (13148), PE-conjugated threonine 183- and tyrosine 185-phosphorylated SAPK/JNK (5755), Alexa Fluor^™^ 488-conjugated serine 396-phosphorylated IRF3 (53539S), Pacific Blue^™^-conjugated IκBα (13656), Alexa Fluor^™^ 647-conjugated serine 536-phosphorylated NFκB p65 (4887) and PE-conjugated NFκB1 p105/p50 (24961) antibodies were obtained from Cell Signaling Technology. PE-CF594-conjugated threonine 180- and tyrosine 182-phosphorylated p38-MAPK (563569) and PE-conjugated tyrosine 705-phosphorylated STAT3 (612569) antibodies were purchased from BD Biosciences. FITC-conjugated BCL-3 (LS-C62564) antibody was bought from LSBio. The PE-conjugated antibody for murine TLR-4 antibody (145404) was purchased from BioLegend. anti-TNFR1 (Armenian hamster IgG, 16-1202-85), its isotype control (Armenian hamster IgG, 16-4888-85), anti-IL-10R (rat IgG1κ, 16-2101-85), its isotype control (rat IgG1κ, 14-4301-85), and anti-IFNAR1 (mouse IgG1κ, 16-5945-85), its isotype control (mouse IgG1κ, 14-4714-85) were from Invitrogen. Dulbecco’s modified Eagle’s medium (DMEM) was from Gibco Life Technologies. Heat-inactivated fetal bovine serum (FBS) was bought from GeminiBio.

### Cells and Cell Culture

Mice were maintained in specific-pathogen-free conditions and all procedures were approved by the NIAID Animal Care and Use Committee (NIH). Bone marrow cells were harvested from femurs and tibias of C57BL/6J (JAX664) mice and bone marrow progenitors were plated on non-tissue treated dishes and differentiated into BMDM during a 6-day culture in complete Dulbecco’s modified Eagle’s medium (DMEM) containing 5% FBS and supplemented with 60 ng/ml recombinant murine M-CSF (Stemcell Technologies) in a water saturated atmosphere at 37°C. For experiments, 2.5x10^6^ BMDM were seeded on non-tissue treated wells of 48 well plates in 5% FBS containing DMEM. On day of experiment, medium was replaced with fresh 5% FBS containing DMEM 30 min before experimental treatment.

### Flow cytometric analyses

FACS analysis of cell surface markers was conducted by placing cells on ice, replacing the medium with 5 mM EDTA/PBS buffer, and transferring them into a 96 well plate. Cells were blocked with 2% FBS and 0.2% goat serum containing HBSS including 1:1000 Mouse BD Fc Block^™^ (Clone 2.4G2, BDBiosciences) and stained with cell surface marker detecting fluorophore-conjugated antibodies. FACS analysis of intracellular proteins was performed by fixing cells with 2.5% PFA for 10 min at room temperature, permeabilizing in 90% methanol for 30 min at −30°C, and blocking with 2% FBS and 0.2% goat serum containing HBSS including 1:1000 Mouse BD Fc Block^™^ (Clone 2.4G2, BDBiosciences). Intracellular proteins were stained with fluorophore-conjugated antibodies overnight at 4°C. Samples were analyzed using a LSRII flow cytometer (BDBiosciences). The percent maximum value was calculated by subtraction of the baseline MFI value and devision of the MFI of each sample by the maximal MFI for each protein species measured within one experiment, multiplied by 100.

### Quantitative real-time PCR

Total RNA was isolated using QiaShredder columns and the RNeasy Mini Kit (Qiagen) according to manufacturer’s instructions. RNA (100 ng) was reverse transcribed into cDNA with iScript cDNA synthesis kit (Bio-RAD) according to manufacturer’s instructions. Gene expression of murine CXCL-1. CXCL-10, CCL-2, IL-6, IL-10, IFN-β, TNF-α BCL-3, SOCS-3 and SDHA was measured with primers for murine CXCL-1 (fw: 5- GCT TGA AGG TGT TGC CCT CAG -3 , rev: 5 - AAG CCT CGC GAC CAT TCT TG -3 ), murine CXCL-10 (fw: 5 - GCC GTC ATT TTC TGC CTC AT -3 , rev: 5 - GCT TCC CTA TGG CCC TCA TT -3 ), murine CCL-2 (fw: 5 - TTA AAA ACC TGG ATC GGA ACC AA - 3 , rev: 5 - GCA TTA GCT TCA GAT TTA CGG GT -3 ), murine IL-6 (fw: 5 -CTC TGC AAG AGA CTT CCA TCC AGT -3 , rev: 5 - GAA GTA GGG AAG GCC GTG G -3 ), murine IL-10 (fw: 5 - AAG GCA GTG GAG CAG GTG AA -3 , rev: 5 - CCA GCA GAC TCA ATA CAC AC -3 ), murine IFN-β (fw: 5 - AAG AGT TAC ACT GCC TTT GCC ATC -3 , rev: 5 - CAC TGT CTG CTG GTG GAG TTC ATC -3 ), murine TNF-α (fw: 5 - CAT CTT CTC AAA ATT CGA GTG ACA A -3 , rev: 5 - TGG GAG TAG ACA AGG TAC AAC CC -3’), murine BCL-3 (fw: 5 - GGA GCC GCG AAG TAG ACG T - 3 , rev: 5 - TGT GGT GAT GAC AGC CAG GT -3 ), murine SOCS-3 (fw: 5 - GCT CCA AAA GCG AGT ACC AGC -3 , rev: 5 - AGT AGA ATC CGC TCT CCT GCA G -3 ), murine JunB (fw: 5’-ATG TGC ACG AAA ATG GAA CA-3’, rev: 5’-CCT GAC CCG AAA AGT AGC TG-3’), murine c-Fos (fw: 5’-CGA AGG GAA CGG AAT AAG ATG-3’, rev: 5’-GCT GCC AAA ATA AAC TCC AG-3’), murine c-Jun (fw: 5’-AAA ACC TTG AAA GCG CAA AA-3’, rev: 5’-CGC AAC CAG TCA AGT TCT CA-3’),and murine SDHA (fw: 5’- TGG GGA GTG CCG TGG TGT CA -3’, rev: 5’- GTG CCG TCC CCT GTG CTG GT -3’). Real-time PCR was performed using Fast SYBR^™^ Green Master Mix (ThermoFisher Scientific, USA) according to manufacturer’s instructions in MicroAmp^™^ Fast Optical 96-Well Reaction Plates Thermo Fisher Scientific, 4346906) with a QuantStudio^™^ 6 Flex Real-Time PCR System (Thermo Fisher Scientific, 4485691). Quantification of gene expression was calculated as described by Pfaffl et al. [35].

### Immunocytochemistry and widefield fluorescence microscopy

For widefield fluorescence microscopy, 1x10^5 BMDM cells were seeded on Falcon 96 well Flat Bottom TC-treated Imaging Microplate (Corning, 353219) and cultivated for 24h before experimental treatment. After treatment, cells were fixed with 2.5% PFA for 10 min at room temperature. Cells were then permeabilized in 70% ethanol for 30 min. After permeabilization, cells were blocked with 2% FBS and 0.2% goat serum containing HBSS including 1:1000 Mouse BD Fc Block^™^ (Clone 2.4G2, BDBiosciences). For detection of endogenous p65, BCL-3 and p50, cells were stained with antibodies specific for p65, BCL-3 and p50 overnight at 4°C. Additionally, DAPI (BioLegend) was added for nuclear staining. Samples were imaged using a CellInsight CX7 Pro HCS Platform (Thermo Fisher Scientific) equipped with an 40x objective lens.

Quantification of the nuclear localization of Alexa Fluor-647-labeled p65, FITC-labeled BCL-3 and PE-labeled p50 were performed by using CellProfiler^™^ for determining the total intensity of fluorophores inside the nucleus, and the relative ratio of intensity of the fluorophores in the nucleus and in the cytoplasm. Nuclear to cytoplasmic ratio was calculated as the nuclear localized fluorophore intensity divided by the intensity of the fluorophore in the cytoplasm.

### Cytokine release quantification

For assessing cyto- and chemokine release, supernatant of treated BMDM cells was taken after putting cells on ice. Supernatant was frozen at −80°C for long-term preservation. After thawing, supernatant was prepared and analyzed with the LegendPlex^™^ Multiplex Assay Kits Mouse Proinflammatory Chemokine Panel (740451) and Mouse Inflammation Panel (740446) according to manufacturer’s instructions by using a LSRII flow cytometer (BDBiosciences). Raw data was further processed with LegendPlex^™^ Desktop software.

### Chromatin immunoprecipitation (ChIP) assay and sequencing

Samples for ChIP assays and sequencing from in vitro cultured BMDMs have been prepared according to Rousselet [36]. After fixation of adherent cells on a 10 cm dish in 1% paraformaldehyde and adding glycine to a final concentration of 125 mM, cells were washed with PBS containing phosphatase and protease inhibitor cocktail Halt^™^ (1:500) (Thermo Fisher Scientific, 78440) ,transferred in a 15 ml tube and centrifuged for 8 min at 300 xg and 4 °C. Pellet was resuspended in SDS-containing lysis buffer and lysates have been passed through a 27Gx1/2 needle to ensure separation of nuclei. Lysates were sonicated with a BioRuptor UCD-300 (Diagenode) for 13 cycles of 30 s on/30 s off, respectively. This was repeated 3 times. To check for the extent of chromatin shearing, 10% of the sample were transferred in a new tube and treated with Proteinase K and RNase and were reverse crosslinked. DNA was then extracted with phenol/chloroform/isoamylalcohol and precipitated with ethanol. DNA was size fractionated via gel electrophoresis on a 1% agarose gel. Successful chromatin fragmentation resulted in a smear of DNA fragments ranging from 100-600 bp. Fragmented chromatin has been pelleted and resuspended in ChIP dilution buffer (see [36]). Samples were pre-cleared with Pierce^™^ ChIP-grade Protein A/G conjugated magnetic beads (Thermo Fisher Scientific, 26162). Beads were removed using a magnetic tube rack and supernatants containing the pre-cleared chromatin were splitted into immunoprecipitation (IP) and input samples. Supernatants of each sample that were used for IP, have been incubated with 10 μg of either anti-BCL-3 (SantaCruz, sc-32741) or it’s corresponding isotype control antibody (Armenian Hamster IgG, Thermo Fisher Scientific, 16-4888-85) overnight at 4 °C. p50 and p65 IP have been performed with anti-p50 (Santa Cruz, sc-8414X) or anti-p65 (Santa Cruz, sc-8008X) antibodies or their respective isotype control antibody (Mouse IgG1 kappa, Thermo Fisher Scientific, 14-4714-85). The next day, in 1.5% fish skin gelatin (Sigma-Aldrich, G7041) and glycogen (Sigma-Aldrich, G8751) pre-blocked Pierce^™^ ChIP-grade Protein A/G conjugated magnetic beads (Thermo Fisher Scientific, 26162) were added to IP samples to bind chromatin/antibody complexes and incubated for 2 h at 4 °C. Tubes were placed in a magnetic tube rack, liquid was removed and beads were washed with a succession of buffers according to Rousselet [36]. Chromatin/antibody complexes were eluted from the beads for 30 min. IP samples and Input samples were reverse crosslinked over night at 65°C in a rotary shaker. After adding Proteinase K and RNase, samples were incubated for 1 h min at 37 °C. DNA was purified using Qiaquick^®^ PCR Purification Kit (Qiagen, 28104).

For analysis using quantitative real-time PCR, IP DNA sample and input sample were used as templates. Real-time PCR analysis of DNA samples was performed according to procedure described in section ‘Quantitative real-time PCR’ using primer for known BCL-3 specific NF-κB sites in the murine TNF-α gene (fw: 5’-CCA GGA GGG AGA ACA GAA ACT C-3’ , rev: 5’- CAC AAG CAG GAA TGA GAA GAG G -3’) [32], murine IL-6 gene (fw: 5’- GAC ATG CTC AAG TGC TGA GTC AC -3’, rev: 5’- AGA TTG CAC AAT GTG ACG TCG -3’) [37] and murine IL-10 gene (fw: 5’- TAG AAG AGG GAG GAG GAG CC -3’, rev: 5’- TGT GGC TTT GGT AGT GC AAG -3’) [38] . Data was analyzed and is given in % of input according to the ‘Constant Volume method’ by Solomon et al. [39].

### Statistical analysis

Statistical analyses were performed with R (version 4.3.0) and RStudio (version 2023.03.1) for MacOS. Data were tested for normal distribution with the Shapiro-Wilk test and homoscedasticity using Levene test. In case of non-normally distributed or heteroskedastic data, nonparametric tests such as Mann–Whitney-U (for single comparison) and Kruskal–Wallis (followed by post hoc Dunn–Bonferroni for multiple comparisons) were applied. For normally distributed data, parametric tests such as *t*-test (for single comparison) and ANOVA (followed by post hoc Tukey for multiple comparisons) were performed. Furthermore, effect size η^2^ was determined. The designations * p < .05, ** p < .01, *** p < .001 denote p-values for the measured differences. If no p-value is indicated, the comparison is considered non-significant. All experiments contained a minimum of three replicates (n = 3).

## Results

### Prolonged stimulation with KLA induces endocytosis of TLR4 and curtails MAPK responsiveness.

**Fig. 1:**
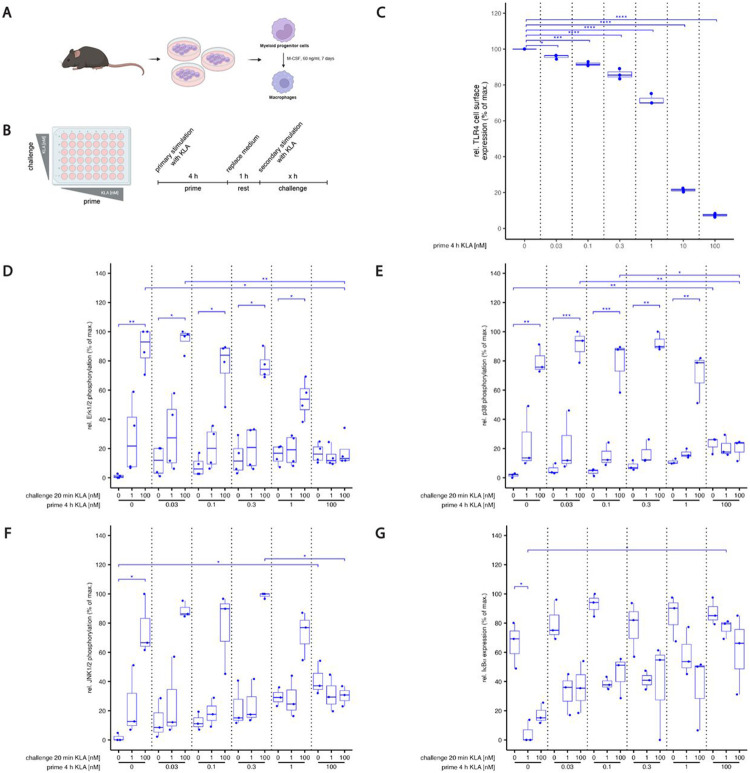
(A,B) Basic experimental protocol used for BMDM generation, priming and restimulation. (A) Bone marrow derived macrophages (BMDMs) were derived from isolated bone marrow cells of wildtype C57BL/6J mice by M-CSF differentiation. BMDMs were treated with varying doses of KLA (prime) for 4 hrs. Then, after replacing the medium, cells are rested for 1 hr and are restimulated (challenge) again with KLA. Depending on the experimental read-out, challenge times differ. **(C) TLR4 internalization becomes stronger as priming concentrations increase.** BMDMs were incubated with 5% FBS DMEM. During priming, cells were stimulated with 0, 0.03, 0.1, 0.3, 1, 10 or 100 nM KLA . After 4 hrs, cells were washed and medium was replaced with 5% FBS DMEM and cells were incubated for 1 hr. Subsequently, cells were washed and stained for TLR4 surface expression. Cell surface expression was determined by using a LSRII flow cytometer (BDBiosciences). Raw data was further processed with FlowJo^™^. Median fluorescence intensities were normalized to the median fluorescence intensity of the TLR4 detecting antibody from a TLR4 KO IMM cellline and is represented in percent of non-primed control sample (set as 100%). **(D-G) Dose dependent activation and adaptation of MAP kinases and IκBa.** BMDMs were incubated with 5% FBS DMEM. During priming, cells were stimulated with 0, 0.03, 0.1, 0.3, 1 or 100 nM KLA . After 4 hrs, cells were washed and medium was replaced with 5% FBS DMEM and cells were incubated for 1 hr. Subsequently, restimulation was performed using 0, 1 or 100 nM KLA. After 20 mins, cells were washed, fixed, permeabilized and intracellularly stained for (D) Erk1/2, (E) p38, (F) JNK1/2, and (G) IkBa expression. Median fluorescence intensity of samples was determined by using a LSRII flow cytometer (BDBiosciences). Raw data was further processed with FlowJo^™^. Data is given in % of maximal median fluorescence intensity within each replicate (set as 100%) and was normalized by subtracting median fluorescence intensity of the sample with the detected minimal median fluorescence intensity (set as 0%). Each replicate was performed with BMDMs from different mice and data sets include at least 4 replicates (n=4). For (C): Kruskal–Wallis test with post hoc Dunn-Bonferroni comparisons of primed samples with untreated sample (planned comparisons) and for (D-G): Kruskal–Wallis test with post hoc Dunn-Bonferroni comparisons: *p < .05, **p < .01, ***p < .001, ****p < .0001.

One of the primary adaptation mechanisms regulating many cellular signaling pathways is the internalization of the stimulatory receptor. However, while membrane-proximal activation of MyD88 is an indispensable step for the activation of the TLR4 pathway [40], important signaling processes involved in cytokine production by macrophages occur after TLR4 endocytosis through the activation of TRIF dependent pathways [41]. This renders the influence of TLR4 internalization more complex than in, for instance chemokine receptor pathways, where internalization typically abrogates cellular stimulation (for review, see [42]). Having previously found a gate-keeping role for MAPK [10], we thus wanted to quantify the relationship between KLA dose, TLR4 internalization and MAPK activation in primary murine bone marrow derived macrophages (BMDM, Fig. 1A,B).

Murine BMDMs were stimulated for 4 hours with KLA concentrations ranging from 0.03 to 100 nM and then stained for cell surface expression of TLR4. KLA reduced the cell surface receptor levels in a dose-dependent manner (Fig. 1C). The higher the KLA priming concentration, the lower the remaining TLR4 expression at the cell surface. Thus, TLR4 availability for restimulation was found to be drastically reduced in cells primed with high doses of KLA.

Next, we stimulated BMDM for 4 h again with KLA concentrations ranging from 0.03 to 100 nM, (‘priming’) then washed the cells and rested them for 1 h before restimulating them with 1 or 100 nM KLA for 20 minutes (‘challenge’), at which time the cells were fixed and stained for phosphorylated Erk1/2, p38, and JNK1/2 and total IκBα. Priming for four hours led to increasingly weak responses upon restimulation as the priming dose was raised, ultimately only allowing for shallow, if any, additional phosphorylation of the MAP kinases (Fig. 1D,E,F), while IκBα degradation showed slightly more sensitivity towards restimulation (Fig. 1G) in line with its somewhat different dose-response characteristics compared to MAP kinases, as we had reported previously [10]. MAPK-dependent target gene expression of AP-1 family members c-Jun and JunB were induced whereas cFos mRNA was not induced by KLA stimulation (Fig. S1 A-C). Importantly, the adaptation seen after 1 nM KLA was likely not caused by strong internalization of TLR4, as its surface expression was still at around 70% of the pre-priming level.

### Cytokine and chemokine responses show quantitative memory of priming stimulation

Having found strong, dose-dependent adaptation at the level of MAPK activation and IκBα degradation, we wanted to assess whether and how such adaptation would be reflected in the cytokine and chemokine responses downstream of these signaling components. Again, murine BMDMs were left unstimulated or were primed for 4 hours with KLA concentrations of 1, 10 or 100 nM. Following washing and a rest period of 1 hour, the cells were either not restimulated (control) or challenged with 1, 10, or 100 nM for another 3 hours. Subsequently, cyto- and chemokine releases were quantified.

**Fig. 2:**
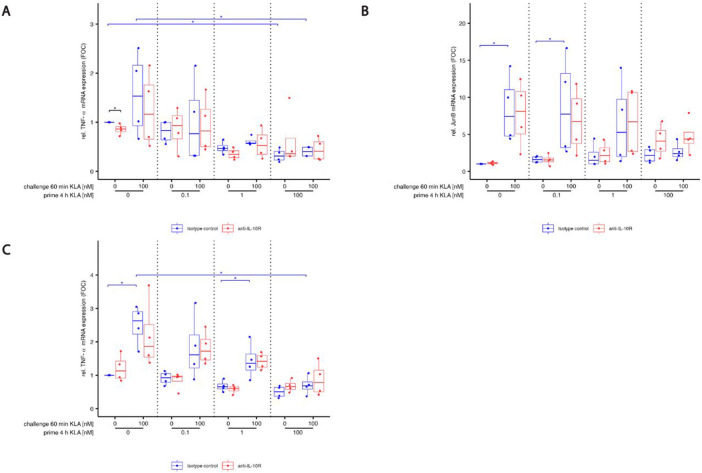
KLA concentration during priming dictates hyper- and hyporesponsive behavior of cyto- and chemokine release in response to restimulation BMDMs were incubated with 5% FBS DMEM. During priming, cells were stimulated with 0, 1, 10 or 100 nM KLA. After 4 hrs, cells were washed and medium was replaced with 5% FBS DMEM and cells were incubated for 1 hr. Subsequently, re-stimulation was performed using 0, 1, 10 or 100 nM KLA. After 3 hrs, supernatants were collected, processed with the LegendPlex^™^ Multiplex Assay Kits and cyto- and chemokine levels for (A) TNF-α, (B) IL-6, (C) CXCL-1, (D) IFN-β, (E) IL-10 and (F) CCL-2 were determined by using a LSRII flow cytometer (BDBiosciences). Raw data was further processed with LegendPlex^™^ Desktop software. Each replicate was performed with BMDMs from different mice and data sets include 6 replicates (n=6). Kruskal– Wallis test with post hoc Dunn-Bonferroni comparisons: *p < .05, **p < .01, ***p < .001, ****p < .0001. (G) Correlation analysis of TNF-α and IL-10 secretion in BMDMs. Spearman correlation coefficients for different prime and re-stimulation (challenge) KLA doses were calculated from samples shown in (A) and (E).

Stimulating *unprimed* cells for 3 hours induced the release of pro- and anti-inflammatory signals in similar amounts for 1, 10 and 100 nM KLA concentrations (Fig. 2). Only IFN-β showed a significantly stronger responses after 100 nM stimulation than after 1 nM (Fig. 2D). Priming for three hours with different KLA concentrations *without restimulation* increased cytokine and chemokine release that became stronger as KLA doses increased (Fig. 2A,B,C,E).

Restimulation of cells primed with 1 nM KLA led to stronger IL-6 secretion than had been observed after stimulation of unprimed cells (Fig. 2B), reflecting previously reported sensitization [10]. Restimulation of BMDMs primed with 10 or 100 nM KLA induced weaker production of TNF-α, IL-6 and CXCL-1 compared to cells that had been primed with 1 nM, indicating the onset of desensitization and the transition to a hyporesponsive state following pro-longed strong TLR4 exposure. Strikingly, in addition to becoming weaker, these secondary responses required restimulation with at least the KLA concentration that was used for priming. Note that neither the MAP kinases nor IκBα showed such graded sensitivity (Fig. 1D-G). Secretion of chemokines CCL-4, CCL-5 and CXCL-10 was induced by KLA and was increased with increasing priming concentrations, while re-stimulation had only a minor impact on those chemokines (Fig. S2)

### IL-10 mediates high-dose priming induced adaptation of inflammatory cytokines

The first series of priming and restimulation assays had not only shown a memory effect in some of the responses but also an abrupt transition of IFN-β production that was completely quenched after priming. On the other hand, IL-10 showed almost no dependence on priming. Since, however, both observations could be due to a lack of resolution in the low priming concentrations, we repeated the measurements, now including 0.03, 0.1 and 0.3 nM, in addition to 1 and 100 nM priming. As we had observed a strong negative correlation between IL-10 and TNF-α after challenging with 100 nM KLA (Fig. 2G), we wanted to quantify the anti-inflammatory effect of IL-10 and performed all measurements also with blocked IL-10 signals by adding non-signaling IL-10R1 blocking antibody.

**Fig. 3:**
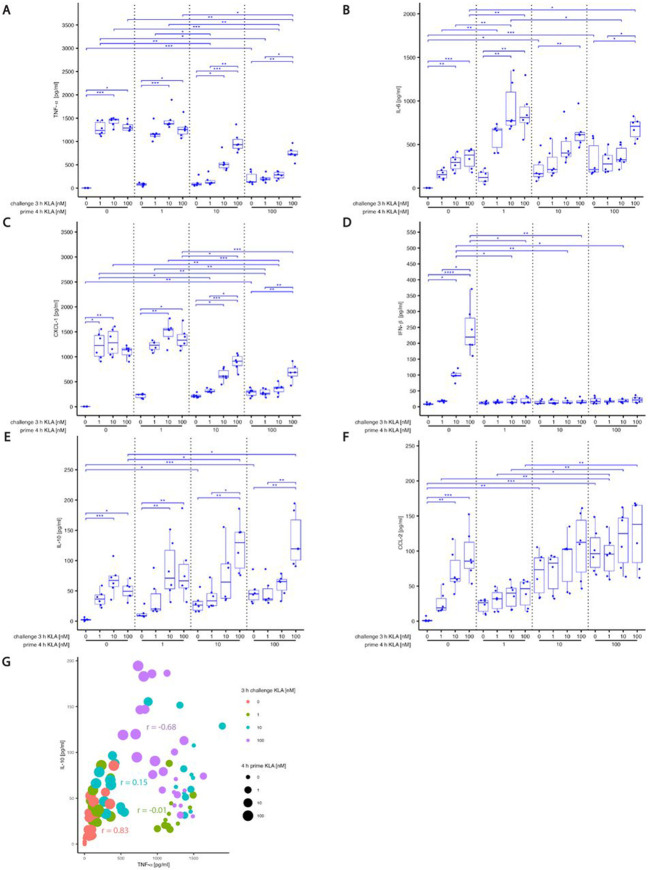
KLA concentration during priming dictates hyper- and hyporesponsive behavior of cyto- and chemokine release in response to restimulation while blocking IL-10 reverses hyporesponsiveness. BMDMs were incubated with 5% FBS DMEM containing either IL-10R blocking antibody or it’s isotype control. During priming, cells were stimulated with 0, 0.03, 0.1, 0.3, 1 or 100 nM KLA . After 4 hrs, cells were washed and medium was replaced with 5% FBS DMEM containing either IL-10R blocking antibody or it’s isotype control and cells were incubated for 1 hr. Subsequently, re-stimulation was performed using 0, 1 or 100 nM KLA. After 3 hrs, supernatants were collected, processed with the LegendPlex^™^ Multiplex Assay Kits and cyto- and chemokine levels for (A) TNF-α, (B) IL-6, (C) CXCL-1, (D) IFN-β, (E) IL-10 and (F) CCL-2 were determined by using a LSRII flow cytometer (BDBiosciences). Raw data was further processed with LegendPlex^™^ Desktop software. Each replicate was performed with BMDMs from different mice and data sets include at least 5 replicates (n=5). Kruskal–Wallis test with post hoc Dunn-Bonferroni comparisons: *p < .05, **p < .01, ***p < .001, ****p < .0001.

Blocking the IL-10 receptor led to a dramatic change in the behavior of the inflammatory cytokines TNF-α and IL-6 and the chemokine CXCL-1: Without IL-10 signals, the previously observed high-dose priming dependent suppression of their responses were completely abrogated (Fig. 3A,B,C compare red to blue). In particular, TNF-α and CXCL-1 production reached saturation following re-stimulation irrespective of the TLR4 activation history when the IL-10 receptor was blocked. Thus, IL-10 signaling interfered with cytokine release in high-dose KLA primed BMDMs after prolonged TLR4 activation. In contrast, cytokine release of low-dose primed BMDMs after re-stimulation was not affected when IL-10 signaling was blocked. Furthermore, blocking IL-10 signaling had little or no effect on chemokine secretion of CCL-4, CCL-5 and CXCL-10 throughout all priming concentrations (Fig. S3).

Responses of IFN-β after strong restimulation steadily decreased as priming concentrations increased, however, much of this adaptation was lost when IL-10 signaling was blocked (Fig. 3D), indicating that IL-10 was mediating a transition to an IL-10 dominated adaptation after stronger priming, a switch that had been suggested recently [43]. IL-10 showed a particularly interesting behavior, with low-dose priming resulting in decreasing responses upon restimulation with high KLA doses. In contrast, beyond a priming dose of 0.3 nM, restimulation-induced production of IL-10 increased again (Fig. 3E).

The results from the protein level analyses were recapitulated when we analyzed cytokine and chemokine mRNA after TLR4 stimulation with and without IL-10 signaling (Fig. S4), indicating that IL-10 mediated regulation was acting by modulating transcription.

### TNF-α signaling contributes strongly while IFN-β affects few cytokine and chemokine responses

Given their established roles as modulators of innate and adaptive immune responses or enhancers of inflammatory responses, respectively, we quantified the influence of blocking signaling through the IFN receptor IFNAR1 and the TNF receptor TNFR for cytokine and chemokine production after 4 hr priming with 0, 0.1 1 or 100 nM KLA followed by 3 hr restimulation with 100 nM KLA.

In these assays, the influence of IFN signaling on cytokines was rather mild, with only IL-10 responses showing an interesting pattern characterized by increased responses after stimulation without priming when the IFNAR1 was blocked but decreased responses after strong (100 nM) priming (Fig. S5). Low and intermediate priming (0.1 and 1 nM KLA) did not lead to differences between macrophages with intact and those with blocked type 1 interferon signaling. Among the chemokines we assessed, CXCL-1 and CXCL-10 showed very distinct dependencies on IFN: At the highest priming concentration (100 nM KLA), CXCL-1 showed enhanced responses after restimulation with 100 nM KLA when the IFNAR1 was blocked, whereas CXCL-10 responses under these conditions were greatly suppressed compared to stimulation with intact IFN signaling.

Our assays in which the TNF-α receptor was blocked mostly confirmed previous work reporting that TNF signaling is enhancing cytokine and chemokine production (Fig. S6).

### Even after strong adaptation, MAPK signals are important for cytokine production

The finding that interfering with IL-10 signals led to a loss of high-dose adaptation for inflammatory cytokines prompted us to test whether reduced MAPK signaling after strong priming still plays a role for downstream induction of cytokine transcription. Since Erk is essential for TLR4-induced cytokine production [10], we applied the MEK-specific inhibitor U0126 after priming, but before restimulation, and compared the resulting levels of activation of signaling components to those obtained without the inhibitor, while also analyzing the dependence of those responses on IL-10 (Fig. S7). Addition of U0126 abrogated Erk responses and reduced the restimulation-induced activation of pro-inflammatory components such as TNF-α, IL-6 and several chemokines after high-dose priming, indicating that even strongly adapted MEK-Erk signaling still contributed to restimulation-induced responses (Fig. S8). Suppressing MEK activation increased IFN-β responses as had been reported before [44]. Blocking IL-10 led, as before, to far stronger inflammatory responses, also when U0126 was applied. While we did not find an influence of IL-10 receptor blocking on Erk phosphorylation, it increased p38 responses (Fig. S7), which may be due to the lack of IL-10-supported and Tristetraprolin mediated production of DUSP1 [45], a phosphatase that may target phosphorylated p38 during TLR activation [46].

### KLA-induced mRNA expression of BCL-3, but not SOCS-3, is modulated by IL-10

Next, we analyzed anti-inflammatory regulatory components with known or potential IL-10 dependencies. SOCS-3 and BCL-3 are two inhibitors of TLR4-induced inflammatory responses in macrophages that are known to be influenced by IL-10 [20, 25, 32]. We therefore assessed mRNA expression of these molecules in primed and restimulated BMDMs.

**Fig. 4:**
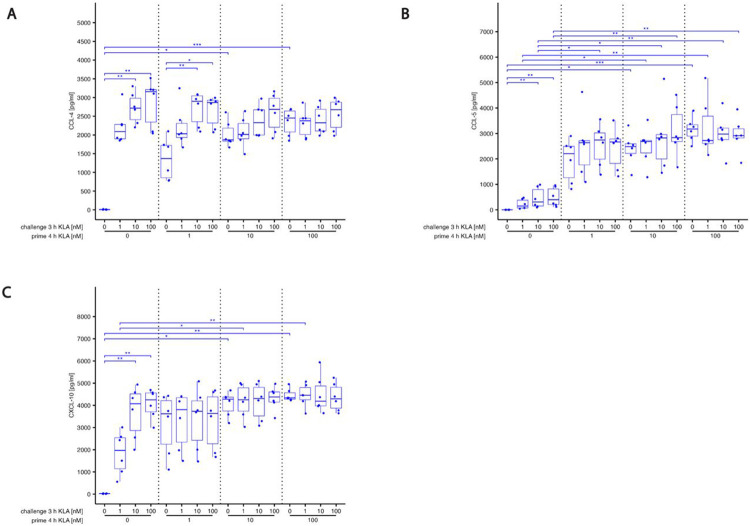
Expression of negative feedback inhibitor SOCS-3 and BCL-3 induced by TLR4 activation depends on priming concentration and is differently affected by IL-10. (A,B) BMDMs were incubated with 5% FBS DMEM containing either IL-10R blocking antibody or it’s isotype control. During priming, cells were stimulated with 0, 0.1, 1 or 100 nM KLA . After 4 hrs, cells were washed and medium was replaced with 5% FBS DMEM containing either IL-10R blocking antibody or it’s isotype control and cells were incubated for 1 hr. Subsequently, restimulation was performed using 0 or 100 nM KLA. After 1 hr, total RNA was isolated and subjected to qRT-PCR analysis to monitor (A) SOCS-3 and (B) BCL-3 mRNA expression. The expression of aforementioned mRNAs was normalized to SDHA mRNA expression. Relative expression of mRNA is given in fold of mRNA expression in naïve and unstimulated control cells. Each replicate was performed with BMDMs from different mice and data sets include at least 5 replicates (n=5). (C-F) BMDMs were incubated with 5% FBS DMEM containing either IL-10R blocking antibody or it’s isotype control. During priming, cells were stimulated with 0, 0.03, 0.1, 0.3, 1 or 100 nM KLA . After 4 hrs, cells were washed and medium was replaced with 5% FBS DMEM containing either IL-10R blocking antibody or it’s isotype control and cells were incubated for 1 hr. Subsequently, re-stimulation was performed using 0 or 100 nM for up to 2 hrs. After depicted times, cells were washed, fixed, permeabilized and intracellularly stained for (C,D) BCL-3 expression as well as (E,F) p50. Expression was determined by using a LSRII flow cytometer (BDBiosciences). Raw data was further processed with FlowJo^™^. Data is given in % of maximal median fluorescence intensity within each replicate (set as 100%). Each replicate was performed with BMDMs from different mice and data sets include at least 3 replicates (n=3). Kruskal–Wallis test with post hoc Dunn-Bonferroni comparisons: *p < .05, **p < .01, ***p < .001, ****p < .0001.

Stimulation of naïve BMDMs increased SOCS-3 mRNA expression within 60 min (Fig. 4A blue). Increasing priming concentrations (without restimulation) led to stronger SOCS-3 mRNA expression. While priming with 0.1 and 1 nM KLA did not affect restimulation-induced mRNA expression compared to naïve cells, priming with 100 nM KLA led to a lack of response of SOCS-3 mRNA in restimulated cells. Blocking IL-10 signals did not significantly modulate SOCS-3 mRNA expression (Fig. 4A, compare red and blue), confirming previous findings suggesting independence of SOCS-3 and IL-10 [28]. The finding that SOCS-3 mRNA expression did not increase after adaptation-inducing KLA doses suggests that SOCS-3 regulation is not part of the mechanisms controlling stimulation dose-dependent adaptation of the TLR4 pathway.

Expression of BCL-3 encoding mRNA was induced by stimulation with 100 nM KLA within 60 min in naïve BMDMs (Fig. 4B, blue). In contrast to the response of SOCS-3, even low-dose (0.1 nM) priming resulted in elevated expression of BCL-3 mRNA compared to unstimulated, naïve BMDMs. Restimulation did not induce additional BCL-3 mRNA expression in primed macrophages. However, in high dose KLA primed macrophages, BCL-3 mRNA expression was reduced when IL-10 signaling was abrogated (Fig. 4B, red).

Since IL-10 was required for induction of BCL-3 mRNA after strong KLA priming, we assessed how much of this IL-10 dependence was reflected in the BCL-3 protein response. We found that BCL-3 was acutely degraded within 20 minutes by restimulation in a dose-dependent manner (Fig. 4C). Later recovery after strong (100 nM) challenge occurred only after no or weak priming and was IL-10 dependent (Fig. 4D). Following strong priming, a 100 nM challenge led to BCL-3 degradation without recovery, independently of IL-10 (Fig. 4D), indicating that, similar to those of SOCS-3, total BCL-3 levels are not upregulated to induce the adaptation observed for high priming concentrations. Another negative regulator of transcriptional activity is p50 [47]. The behavior of p50 was rather similar to that of BCL-3 (Fig. 4E,F).

### TLR4 signals decrease BCL-3 but increase p50 and p65 nuclear intensities

Since BCL-3 is known to be important as anti-inflammatory regulator, its KLA signal induced degradation lacking recovery after strong priming came as a surprise. However, BCL-3, as p50, presumably exerts its inhibitory role in the nucleus, inducing release of transcription factor complexes containing p65 from the DNA [32]. We therefore analyzed microscopy data to quantify its nuclear intensity, together with those of the NFκB family members p50 and p65 (Fig. S9).

**Fig. 5:**
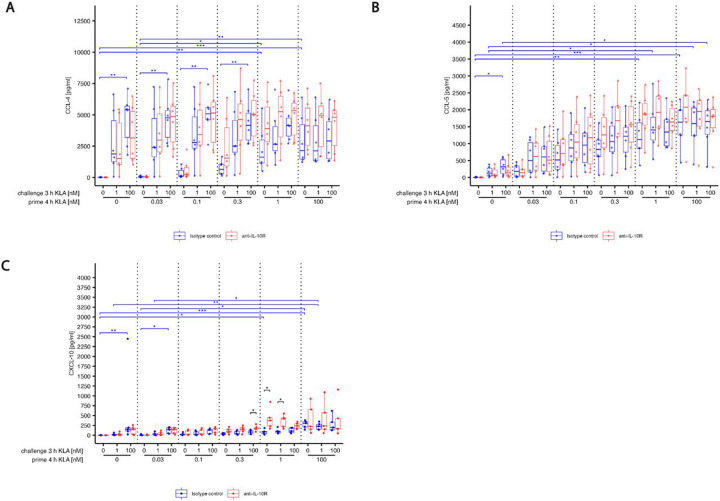
Restimulation induced nuclear localization of p65, p50 and BCL-3 are differently affected by varying priming concentrations and depend on IL-10 signaling. BMDMs were incubated with 5% FBS DMEM containing either IL-10R blocking antibody or it’s isotype control. During priming, cells were stimulated with 0, 0.03, 0.1, 0.3, 1 or 100 nM KLA . After 4 hrs, cells were washed and medium was replaced with 5% FBS DMEM containing either IL-10R blocking antibody or it’s isotype control and cells were incubated for 1 hr. Subsequently, re-stimulation was performed using 0, 1 or 100 nM KLA. After 20 mins, cells were washed, fixed, permeabilized and intracellularly stained for (A,B) p65, (C,D) p50, and (E,F) BCL-3 expression as well as chromatin. Expression and localization were determined by using a CellInsight CX7 Pro HCS Platform (Thermo Fisher Scientific) equipped with an 40x objective lens. Raw data was further processed with CellProfiler^™^. Data is given in relative mean nuclear intensity (A,C,E) and nuclear to cytoplasmic ratio (B,D,F) of the corresponding fluorophore-coupled antibody for p65, p50 or BCL-3. Each replicate was performed with BMDMs from different mice and data sets include 6 replicates (n=6). Kruskal–Wallis test with post hoc Dunn-Bonferroni comparisons: *p < .05, **p < .01, ***p < .001, ****p < .0001.

In line with the direct influence of upstream kinase activation and IκB degradation, restimulation-induced nuclear accumulation of p65 and p50 increased when TLR4 restimulation became stronger but showed adaptation for higher priming concentrations (Fig. 5A-D). Again, BCL-3 proved to be subjected to a different regulation, as its nuclear accumulation simply decreased with increasing priming concentration (Fig. 5E).

The ratios of nuclear to cytosolic intensities of p50 and p65 increased with the TLR4 signal, and mirrored the increases of their nuclear accumulation that, thus were mainly caused by translocation to the nucleus (Fig. 5B,D).

Lack of IL-10 signaling interestingly reduced the nuclear accumulation of both, p50 and p65 compared to the responses seen without the IL-10 receptor block. This suggests that the increased inflammatory responses seen without IL-10 signaling were not due to higher nuclear amounts of either of these two components.

In contrast to p50 and p65, BCL-3 is primarily located in the nucleus (Fig. 5F) in naïve and non-re-stimulated BMDMs. Comparing the dependence of the nuclear intensity of BCL-3 on TLR4 signal and IL-10 (Fig. 5E) with the ratio of nuclear to cytoplasmic BCL-3 we see a stronger loss of nuclear intensity than of the ratio of nuclear to cytoplasmic accumulation when IL-10 signals are absent. This suggests that, indeed, its IL-10-induced production counteracts TLR4-stimulation dependent degradation. Thus, IL-10 signals can, to a certain degree, balance the pro-inflammatory mechanisms that cause BCL-3 reduction. However, assuming that BCL-3 exerts its anti-inflammatory role by interfering with NFκB DNA association, the finding that its nuclear concentration decreased after strong priming and challenge appears to argue against a simple competition with NFκB components.

### Recruitment of BCL-3 to inflammatory gene loci is reduced while p65 recruitment is enhanced without IL-10 signals

Nuclear BCL-3 can interact with p50 via its ankyrin repeats. When p50 is bound to regulatory elements at genes containing κB sites, BCL-3 interaction with p50 stabilizes inhibitory p50 homodimers, thereby inhibiting gene transcription [47]. However, phosphorylated BCL-3 might also facilitate activation of gene transcription by dissociating inhibitory p50 homodimers from regulatory gene elements [48, 49]. It is currently not yet clear if those contrasting roles of BCL-3 in gene regulation appear in parallel on the same or different genes or differ depending on the organism (for review, see [50]).

To see whether BCL-3, p50 and p65 chromatin interaction was affected by different KLA priming doses or by blocking the IL-10 receptor with or without KLA restimulation, we first quantified chromatin recruitment of BCL-3, p50 and p65 by utilizing chromatin immunoprecipitation with BCL-3, p50 and p65. For qPCR analysis of co-immunoprecipitated DNA, we analyzed specific κB sites in the genes coding for TNF-α [32], IL-6 [37] and IL-10 [38] which have been reported previously to be not only NFκB but also BCL-3 recruitment sites.

**Fig. 6:**
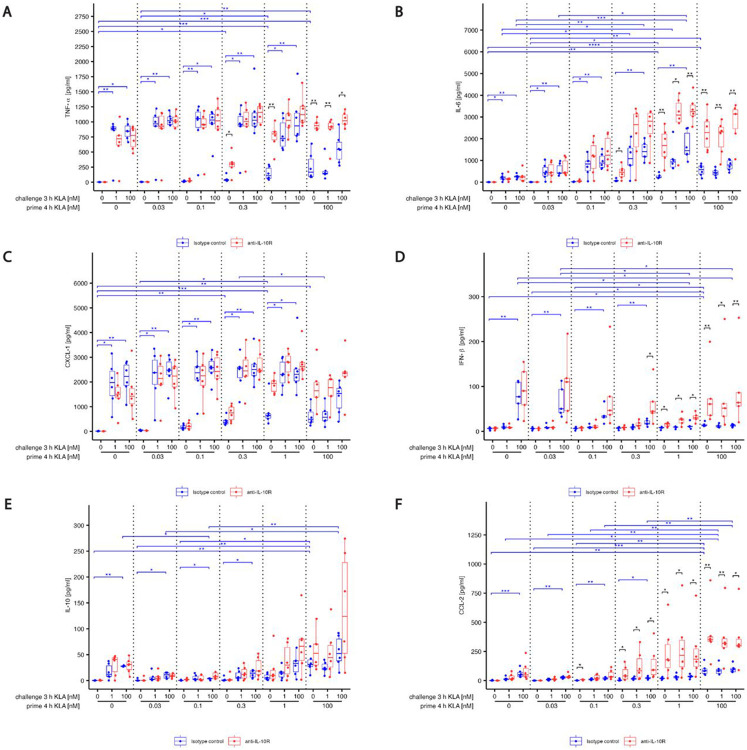
IL-10 dependent adaptation of BCL-3, p50 and p65 association with κB sites in TNF-α, IL-6 and IL-10 genes. BMDMs were incubated with 5% FBS DMEM containing either IL-10R blocking antibody or it’s isotype control. During priming, cells were stimulated with 100 nM KLA . After 4 hrs, cells were washed and medium was replaced with 5% FBS DMEM containing either IL-10R blocking antibody or it’s isotype control and cells were incubated for 1 hr. Subsequently, re-stimulation was performed using 0 or 100 nM KLA. After 20 mins, cells were washed and fixed to cross-link chromatin and protein interactions and chromatin was sheared and further processed. Chromatin immuno-precipitation (ChIP) of either, cross-linked (A-C) BCL-3, (D-F) p50 or (E-G) p65 – chromatin complexes was conducted with BCL-3, p50 or p65 targeting antibodies and, in parallel, with an Isotype control antibody. Protein – chromatin complexes were isolated and reverse cross-linked. DNA was purified and quantified with qPCR using specific primers for kB sites in TNF-α (A,D,G), IL-6 (B,E,FH and IL-10 (C,F,I) genes. The amount of aforementioned chromatin DNA in anti-p65 antibody immunoprecipitated samples was normalized to DNA amounts in their respective anti-Isotype-control antibody immuno-precipitated samples (fold enrichment). Each replicate was performed with BMDMs from different mice and data sets include 4 replicates (n=4). Kruskal–Wallis test with post hoc Dunn-Bonferroni comparisons in (A),(B),(C) and Wilcoxon- test in (D): *p < .05, **p < .01, ***p < .001, ****p < .0001 .

Binding of BCL-3 at κB site in the TNF-α and IL-6 coding genes in naïve macrophages was enhanced whereas binding of BCL-3 at κB site of the IL-10 coding gene was reduced after 20 min challenge with 100 nM KLA (Fig. 6A,B,C). In high dose primed BMDMs, 20 min challenge with 100 nM KLA appeared to increase BCL-3 chromatin binding at the TNF-α locus, but not at the IL-6 and IL-10 loci, even though these changes did not reach significance (Fig. 6A,B,C, blue). However, blocking IL-10 signaling significantly reduced BCL-3 occupancy of κB site in the TNF-α and IL-6 coding genes (Fig. 6A,B, compare red to blue), showing a selective behavior of BCL-3 that was in line with its anti-inflammatory role.

Chromatin binding of p50 in naïve cells appeared to be slightly increased at TNF-α and IL-10 κB sites after 20 min challenge with 100 nM KLA (Fig. 6D,F), but did not reach significance, while binding to the IL-6 site seemed reduced (Fig. 6E), however, again, without reaching significance. Interestingly, high dose primed cells showed a decrease in p50 occupancy of the κB site in TNF-α and IL-10 gene coding chromatin (Fig. 6D,F) while blocking IL-10 prevented challenge-induced reduction of p50 binding to the κB site of the TNF-α coding gene (Fig. 6D, compare red to blue). Finally, in naïve macrophages, interaction of p65 with κB sites in TNF-α and IL-6, but not IL-10, coding genes increased by 20 min stimulation with 100 nM KLA (Fig. 6G,H,I). In high dose primed macrophages, challenge did not increase p65 binding at κB sites in TNF-α, IL-6 and IL-10 coding genes (Fig. 6G,H,I) above baseline. However, blocking IL-10 signaling results in enhanced p65 interaction with a κB site in the TNF-α and IL-10 gene in high dose primed macrophages (Fig. 6G,I compare red to blue), showing the inverse behavior of BCL-3 and thereby making a competition between BCL-3 and p65 for binding to the TNF-α locus and a role of this competition for the observed adaptation plausible.

### Priming with high KLA concentrations enables IL-10 mediated suppression of inflammatory responses

The finding that a loss of IL-10 signaling abrogated priming-induced adaptation of inflammatory responses prompted the question whether this adaptation was a direct result of high IL-10 concentrations produced after strong TLR4 stimulation, as had recently been suggested [43]. To test this, we applied increasing concentrations of recombinant IL-10 to macrophages primed with 0.1 nM KLA, a TLR4 stimulus that had neither induced strong IL-10 production nor led to significant adaptation of any of the signaling components we had investigated (Fig 3). We then challenged the cells with 100 nM KLA and assessed cytokine and chemokine production, comparing the ensuing responses to those obtained after strong (100 nM) KLA priming followed by 100 nM challenge.

**Fig. 7:**
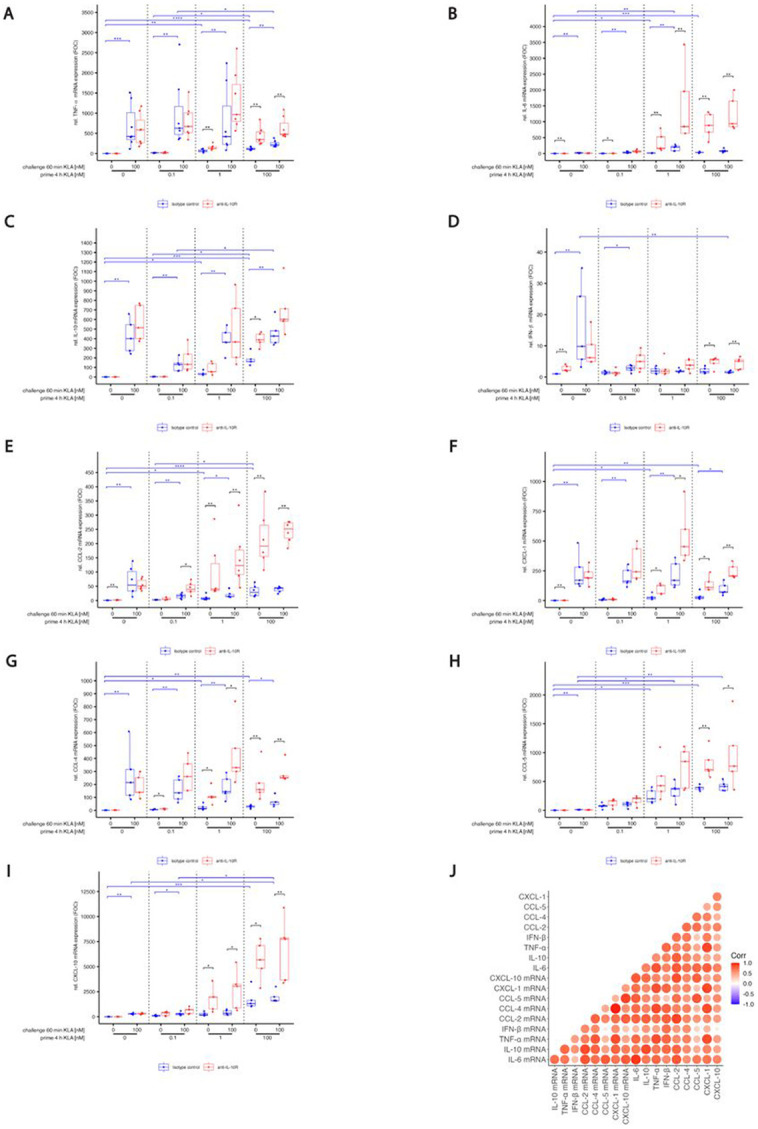
Hypo-responsiveness (Adaptation) induced not only by IL-10 BMDMs were incubated with 5% FBS DMEM. During priming, cells were stimulated with 0, 0.1 or 100 nM KLA. 20 minutes later, recombinant murine IL-10 was added in depicted concentrations. After 4 hrs priming, cells were washed, medium was replaced with 5% FBS DMEM and cells were incubated for 1 hr. Subsequently, re-stimulation was performed using 0 or 100 nM KLA. 20 minutes later, recombinant murine IL-10 was added in depicted concentrations. After 3 hrs re-stimulation, supernatants were collected, processed with the LegendPlex^™^ Multiplex Assay Kits and cyto- and chemokine levels for (A) TNF-α, (B) IL-6, (C) CXCL-1, (D) IFN-β, (E) IL-10 and (F) CCL-2 were determined by using a LSRII flow cytometer (BDBiosciences). Raw data was further processed with LegendPlex^™^ Desktop software. Each replicate was performed with BMDMs from different mice and data sets include 6 replicates (n=6). Kruskal–Wallis test with post hoc Dunn-Bonferroni comparisons: *p < .05, **p < .01, ***p < .001, ****p < .0001.

Unexpectedly, TNF-α showed almost no dependence on exogenously applied IL-10 concentrations and, importantly, did not experience any adaptation without prior strong KLA priming (Fig. 7A). This behavior contrasted with the behavior of IL-6 and IFN-β that showed some increasing adaptation as exogenous IL-10 increased (Fig. 7B,D). Other cyto- and chemokines showed little dependence on either priming or exogenous IL-10 (Fig. 7, Fig. S10).

## Discussion

In this study, we systematically and quantitatively assessed the impact of prior KLA exposure on subsequent restimulation-induced responses in bone marrow derived macrophages. Our assays, following the signal flow along the TLR4 pathway, showed multiple levels of dose-dependent adaptation, from receptor internalization to altered MAPK activation to cytokine specific sensitization or, after priming with high KLA concentrations, strong suppression of inflammatory responses. The dose dependence of the behavior of these different signaling tiers turned out to be very distinct with surprising discrepancies. For instance, priming with a medium concentration of 1 nM KLA led to a reduction of TLR4 surface levels by only about 30%, but downstream MAPK responses to restimulation with the same 1 nM concentration of KLA were completely abrogated, whereas even further downstream, MAPK-dependent production of TNF-α, IL-6 and CXCL-1 under corresponding priming and challenge conditions was, again, only mildly affected.

One of the most surprising results of our study was the observation that TNF-α and CXCL-1 responses showed a memory of prior KLA stimulation and required restimulation with at least the KLA concentration applied during priming to elicit a secondary response. Such an ability of the TLR4 pathway to compare current to previous stimulation strengths may play an important physiological role for macrophage mediated inflammatory responses as it would allow the innate immune system to adjust its behavior to the temporal characteristics of the pathogen challenge it faces. Whereas inflammatory cyto- and chemokine production in response to prolonged exposure to pathogen derived stimuli may, at some point, require attenuation to reduce the risk of tissue damage, innate immune cells have to weigh this risk against the potentially fatal harm stemming from uncontrolled infection. Clearly, the ability to sense whether the pathogen load increases or decreases over time would allow macrophages to carefully balance their behavior in the face of these competing risks. Decreasing TLR4 ligands may indicate a waning pathogenic danger, possibly as a result of a successful immune response, and may permit a reduction of pro-inflammatory signals. Conversely, persistent or even mounting levels of pathogenic stimuli may indicate that further release of inflammatory TNF-α to and of neutrophil recruiting CXCL-1 are required. Our study shows that such assessment of the temporal gradient of molecular indicators of bacterial activity such as KLA, is, indeed, part of the TLR4 signaling pathway.

While the important role of IL-10 for controlling inflammation had been recognized for a long time, our analyses delineate how this role is determined by the strength of TLR4 stimulation. Without IL-10 signaling, the restimulation-induced expression of TNF-α becomes essentially a flat line, showing maximal strength over the entire range of KLA priming concentrations in our assays. With IL-10 signaling intact, TNF-α production following TLR4 restimulation shows two important features: First, responses become attenuated after priming with high concentrations of KLA and second, restimulation with KLA concentrations above the priming strength are required to induce increased TNF-α production. Thus, IL-10 is shown to be the mediator of the macrophages’ ability to tune inflammatory responses to the temporal characteristics of the TLR4 signal. Looking at the activity patterns of the components in the TLR4 pathway suggests that, while MAPK phosphorylation may be the gatekeeper controlling initial inflammatory cytokine activation, other branches of the pathway likely regulate its ability to sense the temporal evolution of the pathogenic challenge. One candidate for this is the IκBα branch due to its graded responses towards KLA concentrations even after strong priming (Fig. 1G).

Another noteworthy observation is how the interplay between IFN-β and IL-10 is shaped by the TLR4 stimulation history. With IL-10 signaling intact, IFN-β had ceased to respond to restimulation following medium to strong priming with 1 or 100 nM KLA. In contrast, when the IL-10 receptor was blocked, it showed graded responses to challenges after such priming. This suggests that macrophages actively switch between IL-10 and IFN-β regulated behavior by suppressing IFN-β responses as IL-10 production rises with increasing KLA stimulation. Such a negative regulatory impact of IL-10 on IFN-β had been reported previously [51] but our study emphasizes the dependence of this effect on the TLR4 stimulation strength. Interestingly, some of this regulation also acts reciprocally, with high IL-10 production requiring some degree of IFN-β signaling (Fig. S4) [52].

Following the quantitative assessment of IL-10’s role for adaptation, we assessed whether it was involved in modulating the abundances of the anti-inflammatory components SOCS-3 and BCL-3. While mRNA for both was induced by TLR4 signals, SOCS-3 showed no dependence on IL-10 signaling. mRNA for BCL-3, in contrast, was significantly lower after strong priming when IL-10 signals were missing, suggesting that, indeed, IL-10 was contributing to BCL-3 anti-inflammatory role as has been previously shown [Kuwata, 2003]. However, subsequent analysis of BCL-3 protein expression levels indicated that, in particular for strong priming followed by strong restimulation, its dependence on IL-10 was weak. Similarly, we found that the nuclear accumulation of BCL-3 was reduced as TLR4 signals increased. This is clearly compatible with the idea that pathogen-derived signals should clear the way for inflammatory anti-pathogenic responses but less so with the anti-inflammatory role that BCL-3 plays for adaptation after prolonged TLR stimulation.

As expected, p50 and p65 showed dose-dependent nuclear accumulation whose responsiveness to TLR4 stimuli became weaker with stronger priming. Perhaps surprisingly however, blocking the IL-10 receptor led to a reduction of their nuclear concentrations, even after strong priming, for which we had seen strongly increased inflammatory responses in the absence of IL-10 signaling. Taking these observations together suggests that the translocation of NFκB components, while providing a readout of TLR4 activation over a wide range of stimulation strengths, does not control the fine tuning of cytokine production in response to acutely changing KLA concentrations, which thus has to happen even further downstream, by regulating the interactions between NFκB components and their target genes.

In fact, quantifying the DNA association of p50, p65 and BCL-3 finally showed the anticipated dependence on IL-10 signals, having reciprocal effects on p65 and BCL-3 accumulation at the TNF-α gene locus: when the IL-10 receptor was blocked, p65 was enhanced at this locus whereas BCL-3 was reduced. However, our single-cell microscopy data on BCL-3 and p65 expression levels and nuclear accumulation and their dependence on priming strengths as well as IL-10 signaling do not provide an explanation.

In many of our assays, we had observed IL-10 dependent adaptation following priming with high concentrations of KLA. One possible explanation for the IL-10 dependence of the suppression of inflammatory cytokine production was that IL-10 production itself was dose dependent, with increasing TLR4 stimulation beyond 0.3 nM priming resulting in stronger IL-10 release. To assess whether, indeed, the KLA concentration dependence of IL-10 provided a complete explanation for the observed adaptation, we combined weak TLR4 priming with IL-10 concentrations corresponding to levels measured after strong priming by applying recombinant exogenous IL-10. To our surprise, high IL-10 concentrations in the context of low KLA priming, while showing some effect on several cytokines and CXCL-1, had no effect on TNF-α production. This means that TNF-α, as the prime inflammatory cytokine with strong feedback attached to it, is controlled by more stringent mechanisms than, for example, IL-6, since strong priming has a far stronger effect on TNF-α than on IL-6. But this regulation requires more than the presence of anti-inflammatory IL-10. It requires the TNF-α secreting macrophage to have experienced persistent TLR4 stimulation of a certain strength. Ultimately, this shows how finely tuned different aspects of the regulation of innate immune responses are and how much mechanistic understanding of the underlying signaling pathways is still missing.

## Supplementary Material

S1 Fig. S1: Blocking IL-10 does not affect AP-1 family member c-Fos, JunB and c-Jun in TLR4 ligand primed and re-stimulated macrophages.
BMDMs were incubated with 5% FBS DMEM containing either IL-10R blocking antibody or it’s isotype control. During priming, cells were stimulated with 0, 0.1, 1 or 100 nM KLA . After 4 hrs, cells were washed and medium was replaced with 5% FBS DMEM containing either IL-10R blocking antibody or it’s isotype control and cells were incubated for 1 hr. Subsequently, restimulation was performed using 0 or 100 nM KLA. After 1 hr, total RNA was isolated and subjected to qRT-PCR analysis to monitor (A) c-Fos, (B) JunB and (C) c-Jun mRNA expression. The expression of aforementioned mRNAs was normalized to SDHA mRNA expression. Relative expression of mRNA is given in fold of mRNA expression in naïve and unstimulated control cells. Each replicate was performed with BMDMs from different mice and data sets include 4 replicates (n=4). Kruskal–Wallis test with post hoc Dunn-Bonferroni comparisons: *p < .05, **p < .01, ***p < .001, ****p < .0001.

S2 Fig. S2: KLA concentration during priming dictates hyper- and hyporesponsive behavior of cyto- and chemokine release in response to restimulation while blocking IL-10 reverses hyporesponsiveness.
BMDMs were incubated with 5% FBS DMEM. During priming, cells were stimulated with 0, 1, 10 or 100 nM KLA. After 4 hrs, cells were washed and medium was replaced with 5% FBS DMEM and cells were incubated for 1 hr. Subsequently, re-stimulation was performed using 0, 1, 10 or 100 nM KLA. After 3 hrs, supernatants were collected, processed with the LegendPlex^™^ Multiplex Assay Kits and cyto- and chemokine levels for (A) CCL-4, (B) CCL-5 and (C) CXCL-10 were determined by using a LSRII flow cytometer (BDBiosciences). Raw data was further processed with LegendPlex^™^ Desktop software. Each replicate was performed with BMDMs from different mice and data sets include 6 replicates (n=6). Kruskal–Wallis test with post hoc Dunn-Bonferroni comparisons: *p < .05, **p < .01, ***p < .001, ****p < .0001. (G) Correlation analysis of TNF-α and IL-10 secretion in BMDMs. Spearman correlation coefficients for different re-stimulation (challenge) KLA doses were calculated from samples shown in (A) and (E).

S3 Fig. S3: KLA concentration during priming dictates hyper- and hyporesponsive behavior of cyto- and chemokine release in response to restimulation while blocking IL-10 reverses hyporesponsiveness.
BMDMs were incubated with 5% FBS DMEM containing either IL-10R blocking antibody or it’s isotype control. During priming, cells were stimulated with 0, 0.03, 0.1, 0.3, 1 or 100 nM KLA . After 4 hrs, cells were washed and medium was replaced with 5% FBS DMEM containing either IL-10R blocking antibody or it’s isotype control and cells were incubated for 1 hr. Subsequently, re-stimulation was performed using 0, 1 or 100 nM KLA. After 3 hrs, supernatants were collected, processed with the LegendPlex^™^ Multiplex Assay Kits and cyto- and chemokine levels for (A) CCL-4, (B) CCL-5 and (C) CXCL-10 were determined by using a LSRII flow cytometer (BDBiosciences). Raw data was further processed with LegendPlex^™^ Desktop software. Each replicate was performed with BMDMs from different mice and data sets include at least 5 replicates (n=5). Kruskal–Wallis test with post hoc Dunn-Bonferroni comparisons: *p < .05, **p < .01, ***p < .001, ****p < .0001.

S4 Fig. S4: KLA concentration during priming affects RNA production of cytokines and chemokines upon restimulation while blocking IL-10 reverses hyporesponsiveness.
BMDMs were incubated with 5% FBS DMEM containing either IL-10R blocking antibody or it’s isotype control. During priming, cells were stimulated with 0, 0.1, 1 or 100 nM KLA . After 4 hrs, cells were washed and medium was replaced with 5% FBS DMEM containing either IL-10R blocking antibody or it’s isotype control and cells were incubated for 1 hr. Subsequently, restimulation was performed using 0 or 100 nM KLA. After 1 hr, total RNA was isolated and subjected to qRT-PCR analysis to monitor (A) TNF-α, (B) IFN-β, (C) CCL-5, (D) IL-6, © CCL-2, (F) CXCL-1, (G) IL-10, (H) CCL-4 and (I) CXCL-10 mRNA expression. The expression of aforementioned mRNAs was normalized to SDHA mRNA expression. Relative expression of mRNA is given in fold of mRNA expression in naïve and unstimulated control cells. Each replicate was performed with BMDMs from different mice and data sets include at least 5 replicates (n=5). Kruskal–Wallis test with post hoc Dunn-Bonferroni comparisons: *p < .05, **p < .01, ***p < .001, ****p < .0001.

S5 Fig. S5: IFN signaling affects IL-10 secretion in naive and high-dose primed BMDMs.
BMDMs were incubated with 5% FBS DMEM containing either IFNAR1 blocking antibody or it’s isotype control. During priming, cells were stimulated with 0, 0.03, 0.1, 0.3, 1 or 100 nM KLA . After 4 hrs, cells were washed and medium was replaced with 5% FBS DMEM containing either IFNAR1 blocking antibody or it’s isotype control and cells were incubated for 1 hr. Subsequently, re-stimulation was performed using 0, 1 or 100 nM KLA. After 3 hrs, supernatants were collected, processed with the LegendPlex^™^ Multiplex Assay Kits and cyto- and chemokine levels for (A) TNF-α, (B) IL-6, (C) IL-10, (D) IFN-β, (E) CCL-2, (F) CXCL-1, (G) CCL-4, (H) CCL-5 and (I) CXCL-10 were determined by using a LSRII flow cytometer (BDBiosciences). Raw data was further processed with LegendPlex^™^ Desktop software. Each replicate was performed with BMDMs from different mice and data sets include 3 replicates (n=3). Kruskal–Wallis test with post hoc Dunn-Bonferroni comparisons: *p < .05, **p < .01, ***p < .001, ****p < .0001.

S6 Fig. S6: TNF signaling enhances secretion of cyto- and chemokines.
BMDMs were incubated with 5% FBS DMEM containing either TNFR blocking antibody or it’s isotype control. During priming, cells were stimulated with 0, 0.03, 0.1, 0.3, 1 or 100 nM KLA. After 4 hrs, cells were washed and medium was replaced with 5% FBS DMEM containing either TNFR blocking antibody or it’s isotype control and cells were incubated for 1 hr. Subsequently, re-stimulation was performed using 0, 1 or 100 nM KLA. After 3 hrs, supernatants were collected, processed with the LegendPlex^™^ Multiplex Assay Kits and cyto- and chemokine levels for (A) TNF-α, (B) IL-6, (C) IL-10, (D) IFN-β, (E) CCL-2, (F) CXCL-1, (G) CCL-4, (H) CCL-5 and (I) CXCL-10 were determined by using a LSRII flow cytometer (BDBiosciences). Raw data was further processed with LegendPlex^™^ Desktop software. Each replicate was performed with BMDMs from different mice and data sets include 4 replicates (n=4). Kruskal–Wallis test with post hoc Dunn-Bonferroni comparisons: *p < .05, **p < .01, ***p < .001, ****p < .0001.

S7 Fig. S7: Influence of MEK inhibition on MAPK activation and p65 phosphorylation.
BMDMs were incubated with 5% FBS DMEM containing either IL-10R blocking antibody or it’s isotype control. During priming, cells were stimulated with 0 or 100 nM KLA . After 4 hrs, cells were washed and medium was replaced with 5% FBS DMEM containing either IL-10R blocking antibody or it’s isotype control and cells were incubated for 1 hr. 30 min prior to re-stimulation, cells have been treated with either U0126 (10 μM) or it’s solvent control DMSO. Subsequently, re-stimulation was performed using 0 or 100 nM KLA. After 20 mins, cells were washed, fixed, permeabilized and intracellularly stained for (A) Erk1/2, (B) p38, (C) p65 phosphorylation. Phosphorylation was determined by using a LSRII flow cytometer (BDBiosciences). Raw data was further processed with FlowJo^™^. Data is given in % of maximal median fluorescence intensity within each replicate (set as 100%) and was normalized by subtracting median fluorescence intensity of the sample with the detected minimal median fluorescence intensity (set as 0%). Each replicate was performed with BMDMs from different mice and data sets include at least 4 replicates (n=4). Kruskal–Wallis test with post hoc Dunn-Bonferroni comparisons: *p < .05, **p < .01, ***p < .001, ****p < .0001.

S8 Fig. S8: MEK inhibition differentially affects pro- and anti-inflammatory cytokine release.
BMDMs were incubated with 5% FBS DMEM containing either IL-10R blocking antibody or it’s isotype control. During priming, cells were stimulated with 0 or 100 nM KLA . After 4 hrs, cells were washed and medium was replaced with 5% FBS DMEM containing either IL-10R blocking antibody or it’s isotype control and cells were incubated for 1 hr. 30 min prior to re-stimulation, cells have been treated with either U0126 (10 μM) or it’s solvent control DMSO. Subsequently, re-stimulation was performed using 0 or 100 nM KLA. After 3 hr, supernatants were collected, processed with the LegendPlex^™^ Multiplex Assay Kits and cyto- and chemokine levels for (A) TNF-α, (B) IL-10, (C) IL-6, (D) IFN-β, (E) CCL-2, (F) CCL-4, (G) CXCL-1 and (H) CXCL-10 were determined by using a LSRII flow cytometer (BDBiosciences). Raw data was further processed with LegendPlex^™^ Desktop software. Each replicate was performed with BMDMs from different mice and data sets include at least 5 replicates (n=5). Kruskal–Wallis test with post hoc Dunn-Bonferroni comparisons: *p < .05, **p < .01, ***p < .001, ****p < .0001.

S9 Fig. S9: Fluorescence microscopy images of p65, p50 and BCL-3 localization
Raw image data shown here is representative for n=6 replicates and images of all 6 replicates were further processed for analysis shown in Fig. 6.

S10 Fig. S10: Hypo-responsiveness (Adaptation) induced not only by IL-10
BMDMs were incubated with 5% FBS DMEM. During priming, cells were stimulated with 0, 0.1 or 100 nM KLA. 20 minutes later, recombinant murine IL-10 was added in depicted concentrations. After 4 hrs priming, cells were washed, medium was replaced with 5% FBS DMEM and cells were incubated for 1 hr. Subsequently, re-stimulation was performed using 0 or 100 nM KLA. 20 minutes later, recombinant murine IL-10 was added in depicted concentrations. After 3 hrs re-stimulation, supernatants were collected, processed with the LegendPlex^™^ Multiplex Assay Kits and cyto- and chemokine levels for (A) CCL-4, (B) CCL-5 and (C) CXCL-10 were determined by using a LSRII flow cytometer (BDBiosciences). Raw data was further processed with LegendPlex^™^ Desktop software. Each replicate was performed with BMDMs from different mice and data sets include 6 replicates (n=6). Kruskal–Wallis test with post hoc Dunn-Bonferroni comparisons: *p < .05, **p < .01, ***p < .001, ****p < .0001.
