# Macrophages tune responses to pathogen dynamics through TLR4 stimulation memory and by licensing susceptibility to IL-10

**DOI:** 10.1101/2024.03.28.587272

**Published:** 2026-07-09

**Authors:** Hannes Bongartz, Christopher T. Boughter, Bernadette Marrero, Thorsten Prüstel, Clinton Bradfield, Julia Gross, Rachel A. Gottschalk, Aleksandra Nita-Lazar, Iain D. C. Fraser, Martin Meier-Schellersheim

**Affiliations:** 1 Computational Systems Biology Section, Laboratory of Immune System Biology Division of Intramural Research National Institute of Allergy and infectious Diseases National Institutes of Health Bethesda, MD, USA; 2 Signaling Systems Section, Laboratory of Immune System Biology Division of Intramural Research National Institute of Allergy and infectious Diseases National Institutes of Health Bethesda, MD, USA; 3 Department of Immunology, University of Pittsburgh School of Medicine, Pittsburgh, PA, USA; 4 Center for Systems Immunology, University of Pittsburgh, Pittsburgh, PA, USA; 5 Functional Cellular Networks Section, Laboratory of Immune System Biology Division of Intramural Research National Institute of Allergy and infectious Diseases National Institutes of Health Bethesda, MD, USA

## Abstract

Macrophages must scale inflammation to the trajectories of infections, escalating as pathogen loads rise, resolving as they fall. The cytokine IL-10 and its chromatin-level effector BCL-3 are critically important anti-inflammatory agents, yet how they avoid muting newly recruited cells before those cells read their own pathogen input has been unclear. Applying systematically varied consecutive TLR4 (Kdo2-Lipid A) stimuli to bone marrow-derived macrophages, we identify two coupled features. First, macrophages retain a quantitative memory of prior stimulation: only when a secondary TLR stimulus matches or exceeds a prior one, IκBα degradation, NF-κB/MAPK activation, and cytokine output increase. Second, IL-10 susceptibility is itself gated by TLR4 history: even a 100-fold IL-10 excess fails to suppress TNF-α in weakly primed cells. Both trace to history- and IL-10-dependent BCL-3 recruitment with p65 displacement at the Tnf κB site. Modeling shows how this licensing logic enables trajectory-aware responses that clear pathogens while limiting tissue damage.

## Introduction

Macrophages, together with other innate immune cells, form the primary defense against many pathogens. They sense pathogen-associated molecular patterns through pattern-recognition receptors such as Toll-like receptor 4 (TLR4), which mediates the response to lipopolysaccharide of gram-negative bacteria and activates the MyD88-dependent mitogen-activated protein kinase (MAPK) and nuclear factor-κB (NF-κB) cascades as well as the TRIF/IRF interferon arm, culminating in the production of pro- and anti-inflammatory cytokines (1–5). Pathogen loads, however, are not static. They rise during the establishment of an infection, plateau, and fall as the pathogen is contained or cleared, and the response that is appropriate at a given moment depends on this trajectory: a cell facing a given current input faces a very different task if the load is still increasing than if it is already being controlled. Sustained inflammation may unnecessarily induce tissue damage when deployed against an already-resolving threat, while premature de-escalation against a still rising load risks failure of pathogen control. Innate immune cells must therefore not merely detect pathogens but also assess whether their exposure is rising or declining.

That macrophages do not respond identically to every TLR4 stimulus has been documented for decades. Low-dose pre-exposure can sensitize cells to subsequent challenges (6–10), while strong or prolonged stimulation produces a hypo-responsive, tolerant state (11–15). A recent systematic study profiled single-cell NF-κB activation dynamics across numerous pairwise combinations of inflammatory ligands in primary macrophages and showed that the dose and duration of prior stimulation are encoded in subsequent NF-κB signaling, with both tolerance and priming arising in ligand- and dose-dependent ways (10). That work, however, varied only the strength of the primary stimulus against a single secondary dose, and focused primarily on combinations of different TLR ligands. Whether the amplitude of a secondary response within the same TLR pathway is set by a quantitative comparison between secondary and primary stimulus strengths has not been resolved. Further, it has remained unexplored whether such a comparison extends from NF-κB dynamics to MAP-kinase activation, IκBα degradation, and downstream cytokine and chemokine output.

At the molecular level, the components of the cellular signaling processes that dampen macrophage activation are well characterized. The anti-inflammatory cytokine IL-10, signaling through STAT3, is a central node (15–29), and the IL-10-inducible atypical IκB family member BCL-3 is an established chromatin-level mediator of cytokine gene suppression (30, 31). What has remained unclear is how anti-inflammatory regulation could preserve the system’s ability to follow the trajectory of an infection. If IL-10 acted on every cell simply as a function of its concentration, then once the early responders in an infected tissue had begun secreting IL-10, every macrophage newly recruited to the site would be immediately conditioned by the ambient IL-10 of its neighbors, muted before it could read its own pathogen input. The same problem arises from IL-10 contributed by other sources: regulatory T cells, B cells, neighboring leukocytes and, at mucosal sites, epithelial- and microbiota-driven signals supply ambient IL-10 at steady state (32, 33). Moreover, several intracellular bacteria and protozoa induce host IL-10 (34) while certain viruses encode IL-10 mimics that engage the host receptor as an immune-evasion strategy (35, 36). Under a concentration-only model, the collective effect of such inputs would be to collapse the trend information that distinguishes a rising load from a falling one into a uniform suppressed state, as opposed to permitting a graded, trajectory-aware response.

Two coupled questions follow that previous work has not fully addressed. First, do macrophages register the strength of prior TLR4 stimulation quantitatively, in a form that lets each cell compare a current input to its own history rather than respond only to its absolute current level? Second, is a cell’s susceptibility to IL-10 itself a fixed property or is it conditioned by its TLR4 history, such that cells which have not yet committed to a TLR4 response remain unaffected by ambient IL-10? Whereas many studies have elucidated the anti-inflammatory action of IL-10 and of its influence on the chromatin level functions of BCL-3, they have largely used uniform strong TLR4 stimulation (16, 23, 26, 31, 37, 38) and have not performed the dose-resolved comparisons required to answer these questions.

In this study, we systematically varied consecutive stimulations of BMDM with the TLR4 ligand Kdo2-Lipid A (KLA), spanning a wide range of doses and dose combinations in primary and secondary stimuli, and titrated exogenous IL-10 against both weak and strong primary stimulation. We find that BMDM register the strength of prior TLR4 input quantitatively: secondary stimuli amplify activation of MAPK, NF-κB and IκBα degradation and increase pro-inflammatory cytokine and chemokine output only when their strengths match or exceeds the primary stimuli, encoding the direction of a temporal trend rather than its instantaneous level. We further find that the susceptibility of BMDM to IL-10 is itself gated by TLR4 history, with even an approximately 100-fold excess of exogenous IL-10 failing to suppress TNF-α production in cells that have not received strong prior stimulation. We trace both features to a chromatin-level mechanism at the Tnf κB site, where TLR4 history and IL-10 jointly govern BCL-3 recruitment with reciprocal displacement of p65, in a manner controlled by the chromatin-binding competence of p50/BCL-3 rather than its abundance or nuclear localization. The result is a coincidence-detector logic that, rather than using them as indiscriminate suppressors, lets the anti-inflammatory components IL-10 and BCL-3 function as part of a regulator reading the cells stimulation histories.

Intuitively, such a cell-intrinsic logic may allow a macrophage population to stay aligned with the trajectory of an infection while indiscriminate suppression of innate function by ambient IL-10 may lead to a failure of pathogen control. To test this intuition quantitatively and explore whether history-gated IL-10 susceptibility may act as a discriminatory suppressor at the population level, we built a simple computational model of macrophages responding to an evolving infection and compared three regulatory logics: (i) history-gated licensing combined with quantitative memory, (ii) suppression set only by the ambient IL-10 concentration, and (iii) no IL-10 regulation of macrophage responses. Robust modeling results indicate that the licensing logic allows the macrophage population to control the pathogen while limiting inflammation-driven tissue damage.

## Results

### TLR4-ligand induced signaling responses show sensitization, adaptation and quantitative memory of primary stimulation

**Fig. 1:**
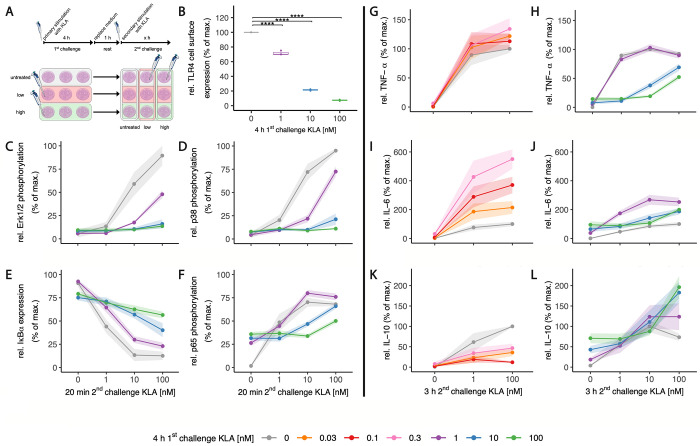
(A) Treatment regimen of 1^st^ challenge and 2^nd^ challenge with KLA of primary murine macrophages. Bone marrow derived macrophages (BMDMs) were differentiated from isolated bone marrow cells of wildtype C57BL/6J mice by M-CSF. Prior to experiment, BMDMs were incubated with 5% FBS DMEM without M-CSF. BMDMs were treated with M varying doses of KLA (1^st^ challenge) for 4 hrs. Then, after washing and replacing the medium, cells were rested for 1 hr and were restimulated (2^nd^ challenge) with N varying doses of KLA resembling a two-stage dosing design yielding an NxM matrix of dose combinations. Depending on the experimental read-out, 2^nd^ challenge times differ. (B) TLR4 internalization becomes stronger as primary KLA concentrations increase. During 1st challenge, cells were stimulated with 0, 1, 10 or 100 nM KLA for 4 hrs. Cells were washed, and medium was replaced with 5% FBS DMEM and were incubated for 1 hr. Subsequently, cells were stained for TLR4 surface expression. Surface expression was determined by using a LSRII flow cytometer (BDBiosciences). Raw data was processed with FlowJo^™^. Median fluorescence intensities were corrected to the median fluorescence intensity of the TLR4 detecting antibody from a TLR4-KO cell line (unspecific binding background), normalized to untreated BMDMs, and represented in percent of untreated control sample (grey, set as 100%). Data include 3 replicates (n=3). Each replicate was performed with BMDMs from different mice. P-values: Kruskal–Wallis test with post-hoc Dunn-Bonferroni comparisons of stimulated samples with untreated sample (planned comparisons). **(C-F) Dose dependent activation and adaptation of MAP kinases, IkBa and p65.** During 1^st^ challenge, cells were stimulated with 0, 1, 10 or 100 nM KLA. Restimulation was performed using 0, 1, 10 or 100 nM KLA. After 20 mins, cells were intracellularly stained for (C) phosphorylated Erk1/2, (D) phosphorylated p38, (E) IkBa, and (F) phosphorylated p65. Median fluorescence intensity of samples was determined by using a LSRII flow cytometer (BDBiosciences). Raw data was processed with FlowJo^™^. Data is given in % of maximal median fluorescence intensity within each replicate (set as 100%) and was normalized by subtracting median fluorescence intensity of the sample with the detected minimal median fluorescence intensity (set as 0%). Each replicate was performed with BMDMs from different mice. Data include at least 4 replicates (n=4) and are shown as mean ± standard error of mean (SEM). (G-L) KLA concentration during 1^st^ challenge dictates responsiveness of cytokine release in response to restimulation. During primary and secondary stimulation, BMDMs were incubated with 5% FBS DMEM. During 1^st^ challenge, cells were stimulated with 0, 0.03, 0.1,0.3 nM KLA (G,I,K) or with 0, 1, 10 or 100 nM KLA (H,J,L). Restimulation was performed using 0, 1, 10 or 100 nM KLA. After 3 hrs, supernatants were collected, processed with the LegendPlex^™^ Multiplex Assay Kits and cytokine levels of (G,H) TNF-a, (I,J) IL-6, and (K,L) IL-10 were determined. Raw data was processed with LegendPlex^™^ Desktop software. Each replicate was performed with BMDMs from different mice. Data include 6 replicates (n=6). Data was normalized to the mean maximal cytokine secretion induced by restimulation in naïve (0 nM KLA primary challenge) cells (set as 100%). Data are shown as mean ± SEM.

To examine how the TLR4 pathway balances pro- and anti-inflammatory output when stimulation strength changes over time, we exposed BMDM to consecutive KLA stimuli applied as systematically varied concentration sequences (Fig. 1A). Because surface TLR4 levels shape the balance between MyD88- and TRIF-dependent signaling (14), we first asked how surface TLR4 changes with KLA dose, extending earlier data limited to single LPS doses or to ≤90 min duration (12, 39). A 4 h primary challenge with 0, 1, 10 or 100 nM KLA reduced surface TLR4 in a dose-dependent manner, leaving ~10% of receptors after 100 nM (Fig. 1B).

To test whether macrophages with a significantly depleted surface TLR4 pool could still discriminate stimulus strength, we washed and rested the cells for 1 h after the 4 h primary challenge (“1st challenge”) and restimulated them for 20 min (“2nd challenge”), the time at which MAPK phosphorylation peaks for intermediate-to-strong signals (8). Increasing primary concentrations progressively weakened secondary MAPK responses and, notably, Erk1/2 and p38 were re-activated only by secondary stimuli exceeding the primary dose: for example, after 1 nM priming, both kinases responded only when the second stimulus surpassed 1 nM (Fig. 1C,D; target genes in Fig. S1A–C).

IκBα degradation and p65 phosphorylation were more readily induced by restimulation, responding when the secondary stimulus matched or exceeded the first and remaining robust even after strong priming (Fig. 1E,F), consistent with our earlier work (8) and indicating that NF-κB signaling stays responsive despite the loss of most surface TLR4.

We next asked whether this behavior propagated to downstream cytokine output. Low-dose priming (≤0.3 nM KLA) sensitized TNF-α and IL-6 responses to secondary challenge, increasingly so with higher primary dose (Fig. 1G,I), however past priming concentrations of 1 nM, this trend changed and secondary responses became progressively reduced (Fig. 1H,J). This was not a full shut-down: after 10 or 100 nM priming, TNF-α and IL-6 were still produced, but only in response to secondary stimuli reaching or exceeding the primary dose – mirroring p65 phosphorylation (Fig. 1F) and indicating that the cells respond selectively to stimuli that surpass their stimulation history. CXCL-1 behaved like TNF-α (Fig. S3C), while IFN-β was an exception, losing responsiveness sharply after priming with ≥1 nM KLA (Fig. S2D, S3D). In contrast to the pro-inflammatory mediators, IL-10 secretion depended non-monotonically on the stimulation sequence, with a minimum of restimulation-induced secretion at low-intermediate priming doses (0.03 −0.3 nM KLA) (Fig. 1K,L; Fig. S2E).

These findings suggested that IL-10-mediated regulation of TNF-α and IL-6 may itself depend on the history of TLR4 stimulation.

### Downstream responses show strongly varying impact of TRL4 stimulation history on IL-10 mediated anti-inflammatory effects

To identify which responses were directly controlled by IL-10, we applied a non-signaling IL-10-receptor-blocking antibody, or an isotype control, throughout primary and secondary stimulation.

**Fig. 2:**
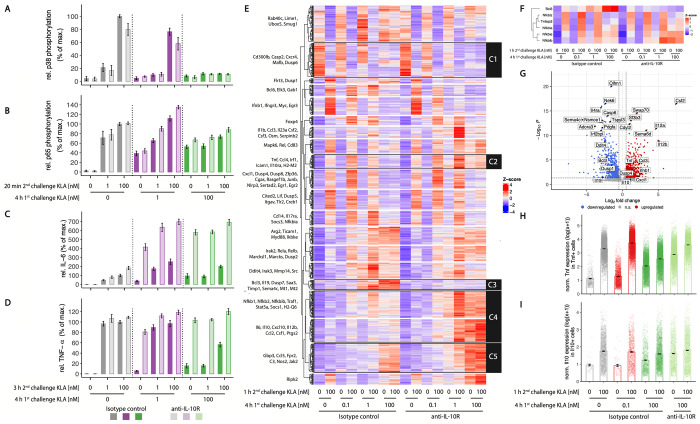
**(A-D) Blocking IL-10 signaling does not substantially affect upstream TLR4-ligand-induced MAPK and NFkB signaling but downstream cytokine secretion.** During primary and secondary stimulation, BMDMs were incubated with 5% FBS DMEM containing either IL-10R blocking antibody (weakly saturated bars) or its isotype control (saturated bars). During 1^st^ challenge, cells were stimulated with 0, 1, or 100 nM KLA. Restimulation was performed using 0, 1, or 100 nM KLA. (A,B) After 20 mins restimulation, cells were intracellularly stained for (A) phosphorylated p38, and (B) phosphorylated p65. Median fluorescence intensity of samples was determined by using a LSRII flow cytometer (BDBiosciences). Raw data was processed with FlowJo^™^. Each replicate was performed with BMDMs from different mice. Data include 4 replicates (n=4), and was normalized to the maximal median fluorescence intensity induced by restimulation in naïve (0 nM KLA primary challenge) cells (set as 100%). (C,D) After 3 hrs restimulation, supernatants were collected, processed with the LegendPlex^™^ Multiplex Assay Kits and cytokine levels of (C) IL-6, and (D) TNF-α, were determined. Raw data was processed with LegendPlex^™^ Desktop software. Each replicate was performed with BMDMs from different mice. Data include 6 replicates (n=6, see Fig. S3,S4), and was normalized to the mean maximal cytokine secretion induced by restimulation in naïve (0 nM KLA primary challenge) cells (set as 100%). Data are given as mean ± SEM. (E-G) IL-10 mediates hypo-responsiveness of pro-inflammatory target genes at transcriptional level. During primary and secondary stimulation, BMDMs were incubated with 5% FBS DMEM containing either IL-10R blocking antibody or its isotype control. During 1^st^ challenge, cells were stimulated with 0, 0.1, 1 or 100 nM KLA. Restimulation was performed using 0 or 100 nM KLA. After 1 hr, total RNA was isolated. (E) RNA from each sample was sequenced and analyzed. Expressed RNA significantly changed by TLR4 activation was clustered according to row-wise Z-scores which were derived from DESeq2-normalized counts, and representative genes for each cluster as well as their differential sensitivity towards primary stimulation and IL-10 were annotated. (F) Gene-expression of several NFkB pathway feedback inhibitors. (G) Differentially expressed gene analysis comparing effect of IL-10 receptor block (without vs. with anti-IL-10R) in 100 nM KLA primary and 100 nM KLA secondary stimulated cells identified IL-10 dependent genes. **(H,I) Gene expression levels in single cells together with size of gene expressing subpopulation determine the TLR4 activation history dependence of pro- and anti-inflammatory response.** During primary and secondary challenge, BMDMs were incubated with 5% FBS DMEM containing either IL-10R blocking antibody or its isotype control. During primary stimulation, cells were treated with 0, 0.1, or 100 nM KLA. Restimulation was performed using 0 or 100 nM KLA. After 1 hr, cells were subjected to single cell RNA sequencing. (H) Normalized Tnf expression in Tnf positive cells. (I) Normalized Il10 expression in Il10 positive cells. Data shown from 2 independent replicates.

Proximal signaling was largely IL-10- independent: p38 phosphorylation showed no dependence on IL-10 under our conditions, in contrast to some earlier reports (24), and p65 phosphorylation was only slightly increased by IL-10R blockade after intermediate (1 nM) priming (Fig. 2A,B). Cytokine output, by contrast, was strongly affected. The negative regulation of IL-6 seen after intermediate and strong priming was IL-10-dependent across all restimulation doses (Fig. 2C). In contrast, TNF-α had shown a much stronger suppression following strong priming (10 or 100 nM) than for priming with up to 1 nM KLA (Fig. 1H) and this suppression was abolished by IL-10R blockade (Fig. 2D). The effect was again selective: CCL-4 and CCL-5 were largely IL-10-independent, whereas CXCL-10 and IL-12p70 increased upon IL-10R blockade, but only after priming with ≥0.3 nM KLA (Fig. S2, S3). IL-10 itself rose when its receptor was blocked, consistent with autocrine feedback control of its expression (40, 41).

These findings suggested that there are complex patterns of gene expression control related to the strength of TLR4 signaling and to IL-10 exposure. To better characterize history-dependent responses and look for coherent clusters of regulated genes at a transcriptome-wide level, we performed RNA-seq on BMDM primed with 0, 0.1, 1 or 100 nM KLA and restimulated with 0 or 100 nM, with or without IL-10R blockade.

A 60 min challenge of naïve cells significantly altered 776 genes (Fold of control ≥ 2; Fig. 2E), which were grouped into multiple clusters, among which five were particularly distinguished by their dependence on KLA dose and on IL 10 (Table 1; full cluster gene lists in Table S1).

The clusters most relevant to adaptation were C2 and C3. C2 comprised pro-inflammatory genes (e.g. Tnf, Ccl4) that show high expression in response to primary stimulation across the dose range used, whereas they exhibit strong negative regulation after high primary KLA stimulation. The latter adaptation is lost upon IL-10R blockade. Notably, the IL-10 receptor itself fell into C2, indicating that strong pro-inflammatory signaling also raises the cells’ capacity to sense IL-10. C3 comprised IL-10-dependent anti-inflammatory genes (e.g. Bcl3, Il19, Dusp7) most strongly induced after 100 nM priming, many associated with the M2 phenotype (42–45).

Some other established negative regulators of TLR4 signaling, such as several members of the NFκB family exhibited patterns of TLR4 stimulation dependencies (Fig. 2F) that were different from the behavior of BCL-3.

Cluster C1, highly expressed in resting cells and progressively lost with increasing KLA, independently of IL-10 (Fig. 2E), includes genes of a tissue-resident identity program (e.g. Mafb, Cxcr4) dismantled during inflammatory activation, while clusters C4 and C5 contained strongly KLA-driven, M1-associated genes differing chiefly in their degree of IL-10 sensitivity. The mRNA profiles of most cytokines and chemokines recapitulated their secretion (compare Fig. S4 with Fig. 1, S2, S3), indicating that the observed regulation is largely transcriptional.

Prior work had shown that population responses of IL-10 were regulated by the fraction of IL-10 producers whereas single-cell TNF-a production may vary depending on the TLR4 stimulus the cells received (9, 46, 47). We therefore analyzed how TLR4 stimulation history shaped single cell expression levels of TNF-α and IL-10. The 0.1/100 protocol generated more high-Tnf cells than 100/100 and closely resembled 100/100 with the IL-10 receptor blocked (Fig. 2H). The history dependence of TNF-α output is therefore encoded in per-cell expression intensity rather than in the size of the producing population, resolving the apparent discrepancy with the secretion data (Fig. 1G,H). Il10 showed the converse: per-cell expression was largely invariant across protocols (Fig. 2I, Fig. S7), so that total IL-10 output was set by the fraction of producing cells (9, 46, 47).

### Susceptibility to IL-10 requires strong prior TLR4 stimulation, not merely IL-10 itself

In contrast to the behavior of IL-6 (Fig. 2C), the IL-10-dependent hypo-responsiveness of TNF-α occurred only in macrophages pre-exposed to the highest KLA concentrations (Figs. 2D, S2, S3). One potential explanation was that suppressing TNF-α requires more IL-10 than suppressing IL-6 – which is directly controlled by IL-10/STAT3 – so that only strong TLR4 stimulation generates sufficient IL-10 (46). To test this, we primed BMDM for four hours with a low (0.1 nM) or high (100 nM) KLA dose, restimulated for three hours with 100 nM KLA, and titrated recombinant IL-10 from 20 to 20,000 pg/ml, supplied during both, primary and secondary stimulation. Importantly, 200 pg/ml corresponded to the maximum IL-10 produced by the cells themselves after 100 nM KLA (Fig. S3 E) and was sufficient to suppress pro-inflammatory output after strong priming with 10 or 100 nM KLA (Fig. S3 A, B, C).

**Fig. 3:**
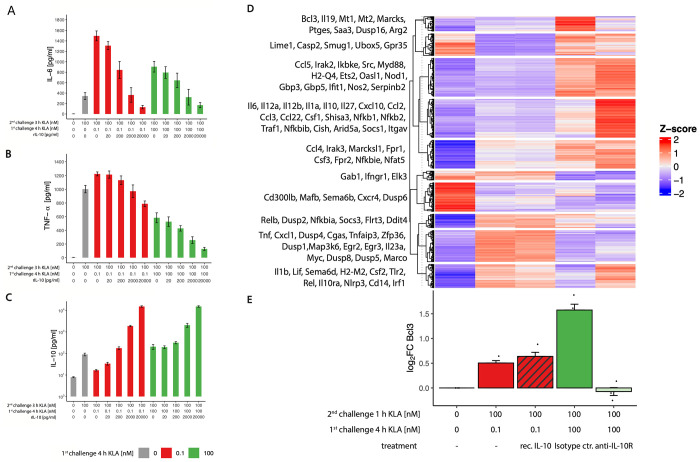
Broad induction of hypo-responsiveness requires prior strong TLR4 simulation, not just IL-10 (A-C) During primary stimulation, cells were treated with 0, 0.1, or 100 nM KLA. Restimulation was performed using 0 or 100 nM KLA. 20 min after each KLA stimulation, recombinant murine IL-10 was added in depicted concentrations. After 3 hrs restimulation, supernatants were collected, processed with the LegendPlex^™^ Multiplex Assay Kits and cytokine levels of (A) IL-6, (B) TNFα, and (C) IL-10 were determined. Raw data was processed with LegendPlex^™^ Desktop software. Each replicate was performed with BMDMs from different mice. Data include 9 replicates (n=9) and are given as mean ± SEM. (D,E) During primary and secondary challenge, BMDMs were incubated with 5% FBS DMEM containing either IL-10R blocking antibody or its isotype control. During primary stimulation, cells were stimulated with 0, 0.1, or 100 nM KLA. Restimulation was performed using 0 or 100 nM KLA. 20 min after each KLA stimulation, 200 pg/ml of recombinant murine IL-10 was added. After 1 hr restimulation, total RNA was isolated, sequenced and analyzed. (D) Expressed RNA significantly changed by TLR4 activation was clustered according to row-wise Z-scores which were derived from DESeq2-normalized counts. (E) Log_2_ fold changes for Bcl3 mRNA expression were calculated from DESeq2-normalized counts as mean expression ratios versus control (pseudocount = 1). Data are given in mean ± SEM of n=4 replicates.

Consistent with an unconditional susceptibility towards suppression by high concentrations of IL-10, IL-6 responses were suppressed below the level seen after strong TLR4 priming (Fig. 3A) by IL-10 concentrations above 200 pg/ml.

Confirming that the role of IL-10 for the regulation of TNF-α responses is quite different from its role for IL-6, even the highest concentrations of exogenous IL-10 failed to suppress TNF-α production to the level reached after strong priming (compare rightmost red column to leftmost green column in Fig. 3B). This suggests that the divergent behavior of IL-6 and TNF-α does not simply reflect different sensitivities to IL-10.

The responses of other cytokines and chemokines towards combinations of weak or strong TLR4 stimulations and exogenous IL-10 were highly heterogenous (Fig. S5). Interestingly, high concentrations of recombinant IL-10 could strongly suppress IFN-β and CXCL-1, however not beyond the suppression seen for strong TLR4 stimulation (Fig. S5 A, B).

In contrast, CCL-2 and CXCL-10 were only weakly secreted after low-dose pre-stimulation and showed only limited susceptibility to exogenous IL-10, while strongly pre-stimulated cells secreted higher levels of these chemokines and became responsive to IL-10-mediated suppression (Fig. S5C,F).

Macrophages pre-stimulated with 0.1 nM KLA and subsequently treated with 200 pg/ml exogenous IL-10 exhibited reduced IL-10-induced STAT3 phosphorylation compared with naïve cells treated with the same concentration of IL-10 (Fig. S5H). This suggests that susceptibility of macrophages towards the anti-inflammatory IL-10 may be determined by their TLR4 activation history in a target-gene specific manner.

Transcriptome-wide, the difference between the behavior of Il6 and that of other genes was striking. Reminiscent of what we had observed for TNF-α and IL-6 production (Fig. 3A, B), 200 pg/ml recombinant IL-10, although sufficient to suppress IL-6, altered only a small subset of genes in low-dose-primed macrophages (Fig. 3D), with the Il6-containing cluster the only one whose behavior approached that of strong priming. Direct suppression of IL-6 thus appears to be the exception rather than a model for the general action of IL-10 as anti-inflammatory agent. Consistent with this, the Bcl3-containing cluster showed almost no response to exogenous IL-10 when cells had received only weak priming, whereas dual strong stimulation strongly upregulated Bcl3 in an IL-10-dependent manner (Fig. 3E). Although very high IL-10 concentrations were found to induce BCL-3 previously (31), the highest IL-10 levels produced in our assays, while sufficient to suppress IL-6, did not induce Bcl3 without prior strong TLR4 stimulation. Post-transcriptional control may also contribute: Zfp36, encoding the TNF-mRNA-destabilizing protein TTP, depended strongly on TLR4 history but only weakly on IL-10 (Fig. 2E, 3D), in line with prior reports (40, 48).

### TLR4 history and IL-10 condition the chromatin association of BCL-3 independently of its nuclear abundance

**Fig. 4:**
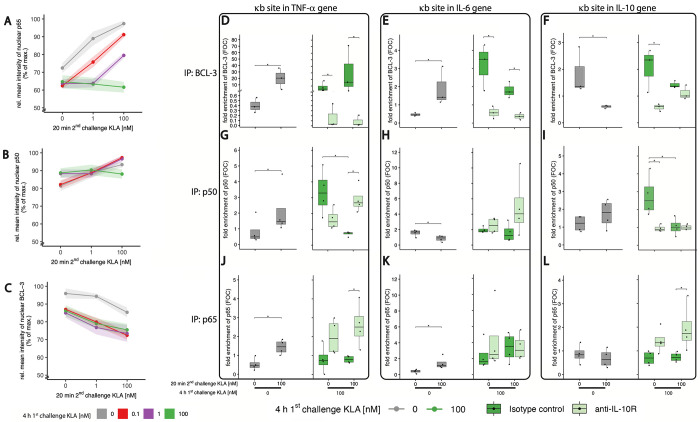
TLR4 primary and secondary stimulation as well as Il-10 signaling affect nuclear localization and binding of BCL-3, p65 and p50 at kB sites of target genes. (A-C) During primary and secondary stimulation, BMDMs were incubated with 5% FBS DMEM containing either IL-10R blocking antibody or its isotype control. During 1^st^ challenge, cells were stimulated with 0, 0.1, 1 or 100 nM KLA. Restimulation was performed using 0, 1 or 100 nM KLA. After 20 mins, cells were intracellularly stained for (A) p65, (B) p50, and (C) BCL-3 expression and localization was determined by using a CellInsight CX7 Pro HCS Platform (Thermo Fisher Scientific) equipped with an 40x objective lens. Raw data was processed with CellProfiler^™^. Data is given in % of maximal median fluorescence intensity within each replicate (set as 100%). Each replicate was performed with BMDMs from different mice. Data include at least 6 replicates (n=6) and are given as mean ± standard error of mean (SEM). (D-L) During primary and secondary challenge, BMDMs were incubated with 5% FBS DMEM containing either IL-10R blocking antibody or its isotype control. During 1^st^ challenge, cells were stimulated with 0 or 100 nM KLA. Restimulation was performed using 0 or 100 nM KLA. After 20 mins, cells were fixed to cross-link chromatin and protein interactions and chromatin was sheared. Chromatin immuno-precipitation (ChIP) of either, cross-linked (D-F) BCL-3, (G-I) p50 or (J-L) p65 – chromatin complexes was conducted with BCL-3, p50 or p65 targeting antibodies and, in parallel, with corresponding Isotype control antibodies. DNA from purified Protein – chromatin complexes was quantified with qPCR using specific primers for kB sites in TNF-a (D,G,J), IL-6 (E,H,K) and IL-10 (F,I,L) genes. The amount of chromatin DNA in anti-BCL-3, anti-p50 and anti-p65 antibody immunoprecipitated samples was normalized to DNA amounts in their respective Isotype-control antibody immuno-precipitated samples (fold enrichment). Each replicate was performed with BMDMs from different mice and data include at least 3 replicates (n=3). P-values: Wilcoxon test for KLA stimulation in naïve BMDMs (grey) and Kruskal–Wallis test with post-hoc Dunn-Bonferroni comparisons for 100 nM KLA pre-exposed BMDMs (± anti-IL-10R, green and light-green).

Because BCL-3 contributes to transcriptional repression by competing with p65 for p50-mediated κB-site binding (30, 37, 38, 49), we first asked whether the nuclear abundance of BCL-3, p50 and p65 tracked target-gene regulation. Restimulation-induced nuclear accumulation of p50 and p65 increased with stronger secondary KLA but ceased to increase after 1 and 100 nM priming (Fig. 4A,B; Fig. S6B,C,E,F), consistent with upstream IκBα degradation and p65 phosphorylation (Fig.1E,F). BCL-3 was predominantly nuclear (Fig. S6G), but its nuclear accumulation decreased with higher primary doses (Fig. 4C). IL-10R blockade only slightly reduced the nuclear levels of all three factors (Fig. S6B–D). Neither nuclear accumulation nor mRNA levels of BCL-3, p50 or p65 therefore predicted target-gene suppression, arguing against a simple mass-action competition and indicating that nuclear NF-κB translocation cannot be used to quantify the strength of upstream receptor stimulation.

We therefore examined factor occupancy directly by chromatin immunoprecipitation followed by qPCR at known κB sites in the TNF-α, IL-6 and IL-10 genes. In naïve cells, a 20 min 100 nM challenge enriched BCL-3 at the TNF-α and IL-6 κB sites and reduced it at the IL-10 site (Fig. 4D–F, grey bars); p50 and p65 were both enriched at the TNF-α locus, whereas the IL-6 locus lost p50 but gained p65 (Fig. 4G,H,J,K, grey bars), consistent with p50 negatively regulating TNF-α but not IL-6 (31).

The central result here concerned the TNF-α κB site after strong TLR4 history, where TLR4 history and IL-10 signaling produced opposing, coordinated changes in factor occupancy. Strong priming drove BCL-3 accumulation that was maintained upon restimulation (Fig. 4D), while p65, recruited by acute stimulation, was instead reduced by prolonged strong stimulation and remained suppressed upon restimulation (Fig. 4J). Blocking the IL-10 receptor reversed both: BCL-3 occupancy fell by roughly two orders of magnitude and p65 occupancy was restored (Fig. 4D,J). Because cell-wide and nuclear BCL-3 levels changed only modestly under IL-10R blockade (Fig. S6D), the loss of promoter-bound BCL-3 likely reflects an IL-10-dependent change in its chromatin-binding competence rather than in its abundance. p50 was likewise enriched at the TNF-α κB site after prolonged stimulation but fell to basal levels upon acute restimulation, and, in the absence of IL-10 signaling, restimulation instead increased p50 alongside the IL-10-dependent gain of p65 (Fig. 4G,J). By contrast, the IL-6 and IL-10 loci showed more modest, largely IL-10-independent changes. The IL-6 κB site exhibited only subtle variation in p50 and p65 occupancy (Fig. 4H,K). At the IL-10 κB site, strong stimulation recruited p50, consistent with its role in promoting IL-10 expression. But restimulation did not further increase it. p50 enrichment was abolished by IL-10R blockade even though IL-10 production itself rose, indicating an interrupted autocrine feedback loop (Fig. 4I). BCL-3 enrichment at the IL-10 site was only slightly greater with intact than with blocked IL-10 signaling, suggesting an additional IL-10/STAT3-independent contribution to its recruitment there.

### History-gated IL-10 licensing enables trajectory-aware control of infection in a computational model

To explore and illustrate the possible functional consequences of the IL-10 licensing logic, we implemented an ordinary differential equation (ODE) model of a macrophage population responding to a pathogen challenge over a simulated time course of 10 days. Depending on the inflammatory response, pathogen levels may plateau after an initial rise and then get cleared or may escape (full description in Supplementary Methods Text S1).

TLR4 signaling has been explored in modeling work that focused on details of intracellular signaling (50–52) or more complex innate responses regulating, for instance, wound healing (53). Interestingly, among those prior molecular-level modeling studies was a qualitative assessment of pre-stimulation dependent adaptation based on negative feedback stemming from A20 production (52).

Compared to those studies, the model we used is rather simple as it is limited to exploring the different trajectories an infection may take depending on the type of phenomenological anti-inflammatory regulation the responding macrophages experience. The model assumes that macrophages are recruited in proportion to inflammation and, reflecting the experimental findings presented here, retain a quantitative memory of prior stimulation that establishes graded activation set points governing subsequent responsiveness. They re-activate only when the current stimulus matches or exceeds their remembered set-point, corresponding to the match-or-exceed behavior reported in Fig. 1. IL-10 is produced in proportion to the cells’ TLR4 exposure. To assess the effects of IL-10 licensing, we generated three variants of this model that, importantly, differ only in how IL-10 acts on the cellular inflammatory response: under history-gated licensing (variant 1), only cells that have committed to a strong TLR4 response are susceptible to IL-10. This variant implements history-gated IL-10 licensing on the basis of single-cell experimental data on the dependence of TNF-α and IL-10 production on TLR4 stimulation history (Figs. 2H, I, Fig. S2E, Fig. S7 and Supplementary Text 1 model notes). Accordingly, and in an interesting coincidence reported by our data, cells start producing IL-10 as soon as they can sense its regulatory effects. Under the concentration-only variant (variant 2), every cell is suppressed in proportion to the ambient IL-10 level; and under the no-regulation variant (variant 3), IL-10 has no effect.

**Fig. 5:**
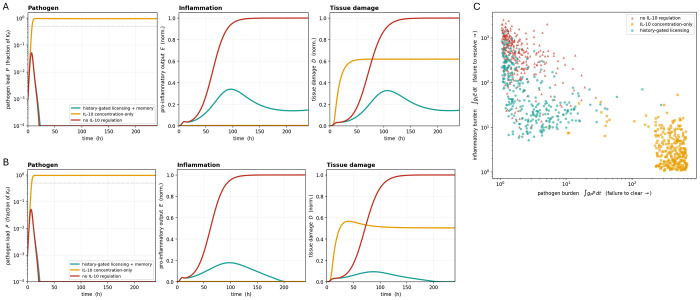
History-gated IL-10 licensing enables trajectory-aware control of infection in a computational model. **(A) Base-model infection time courses for the three regulatory variants.** Standard parameter set; history-gated licensing + memory (teal), IL-10 concentration-only (orange), no IL-10 regulation (red). Left panel: pathogen load (fraction of carrying capacity, log axis): concentration-only pre-mutes cells and the pathogen escapes to carrying capacity, whereas licensing+memory and no-regulation peak near 5% and clear it within ≈25 h. Center panel: pro-inflammatory output (normalized to its cross-variant maximum): absent in the escaped concentration-only case, transient-then-resolving under licensing+memory, sustained under no-regulation. Right panel: tissue damage (normalized): no-regulation locks into a non-resolving sterile-inflammation state, concentration-only incurs pathogen-driven injury, licensing+memory peaks then partially resolves. **(B) Active resolution by M1→M2 polarization.** Time courses with the resolution arm at the standard parameter set; colors as in (**A**). Left panel: pathogen (log axis); Center panel: inflammation; Right panel: tissue damage (center and right normalized to their cross-variant maxima). IL-10 now performs three coupled roles — acute suppression of licensed cells, the match-or-exceed memory gate, and driving the most-adapted cells into the M2-like state. Under licensing+memory the response resolves fully (final D → 0); no-regulation makes no M2 (IL-10R inert) and stays chronically inflamed; concentration-only again leads to pathogen escape. **(C) Two-objective decomposition of the parameter sweep.** Each point is one Latin-hypercube draw (resolution model with M2-like state), plotted by its pathogen burden (horizontal axis; rightward = failure to clear pathogen) against its inflammatory burden (vertical axis; upward = failure to resolve inflammation). The two alternatives to the history-gated licensing fail on opposite axes: concentration-only (orange) toward high pathogen burden, no-regulation (red) toward high inflammatory burden. History-gated licensing (teal) is the only variant that strongly populates the low-burden corner, clearing and resolving together.

The three model variants produce markedly different outcomes (Fig. 5A), and these differences are robust under parameter variation (Fig. S11 in Text S1). Under history-gated licensing (variant 1), naive cells respond to the rising pathogen and clear it within the first day (Fig. 5A, left panel; the teal curve is hidden beneath the red no-regulation curve, which clears the pathogen equally well). Inflammation and tissue damage rise transiently and then declined as the pathogen is cleared and committed cells are restrained by IL-10, settling at a low residual plateau (Fig. 5A, center and right panels). This incomplete resolution reflects the absence of an active resolution program in the base model: with no pro-resolving phenotype to clear accumulated damage, a low-grade damage-associated (DAMP) signal sustains residual inflammation. Under IL-10 concentration-only suppression (variant 2), ambient IL-10 mutes newly recruited cells before they can respond, leading to pathogen escape to its carrying capacity (Fig. 5A, left panel, orange curve) and sustained, pathogen-driven injury (Fig. 5A, right panel, orange curve). The tissue damage in this variant therefore stems from the pathogen load rather than from inflammation, which is suppressed. In the no-regulation variant (variant 3), the pathogen is cleared, but inflammation and tissue damage fail to resolve, locking the tissue into a chronic, self-sustaining inflammatory state (Fig. 5A, red curves). Note that, in the failure modes, comparable injury is caused by opposite mechanisms: unchecked pathogen in the concentration-only variant versus unresolved immunopathology in the no-regulation variant. Only history-gated licensing both controlled the pathogen and limited tissue damage. The reason is that newly recruited naive cells are not yet licensed and so remain free to respond to a rising challenge even in the presence of ambient IL-10, whereas IL-10 restrains only cells that have already committed to a strong response. The population therefore tracks the direction of the pathogen trajectory rather than the absolute IL-10 level, recapitulating the trajectory-aware behavior implied by our data (Fig. 1).

In an extension of this model, and based on the gene-expression characteristics in cluster C3 of Fig. 2E, we further allowed the most strongly adapted, IL-10-suppressed macrophages to polarize toward a pro-resolving (M2-like) state that would eventually withdraw from inflammatory output and promote clearance of damaged tissue, reflecting the established coupling between IL-10 signaling and resolution programs (54). With this extension, the history-gated licensing variant fully clears the pathogen and resolves the inflammation, whereas the concentration-only variant still produces persistent tissue damage through its failure to clear the pathogen, and the no-regulation variant remains locked in uncontrolled inflammation (Fig. 5B).

While the model implements history-gated IL-10 licensing on the basis of single-cell experimental data, its kinetic parameters were not fitted to particular experiments. To assess the robustness of the licensing variant’s apparent advantage over the two alternatives, we performed parameter sweeps for which the parameter sets were drawn by Latin-hypercube sampling (55) to systematically cover the parameter space. Across the sampled space, the two alternatives to licensing failed along opposite axes: the concentration-only variant toward high pathogen burden and the no-regulation variant toward high inflammatory burden. Only the licensing variant consistently reached the low-burden region in which both pathogen and inflammation are controlled (Fig. 5C; per-parameter sensitivities from a Partial Rank Correlation Coefficient analysis can be found in Fig. S11). This structural separation suggests that the TLR4 stimulation memory and IL-10 licensing we identified in vitro may contribute to balancing pathogen clearance and tissue damage during macrophage responses to infection.

## Discussion

Macrophages and other innate immune cells must scale their inflammatory output to pathogen burdens that may change over time: responding vigorously when a threat escalates while limiting collateral damage to host tissue when it does not. By exposing BMDM to systematically varied sequences of KLA concentrations, we found that this balance is achieved, in part, through a quantitative memory of prior TLR4 activation and through a stimulation-history-dependent control of the cells’ own susceptibility to IL-10. Our data confirm established features of TLR4 sensitization and tolerance and the involvement of IL-10, BCL-3, p50 and p65 (6–16, 19, 20), but they additionally reveal three previously unrecognized aspects of macrophage adaptation.

First, MAPK and NF-κB signaling did not merely become blunted after prior stimulation but retained a quantitative memory of it: secondary responses required a stimulus that matched (p65) or exceeded (Erk1/2 and p38) the strength of the primary challenge, and the same match-or-exceed behavior propagated to the secreted pro-inflammatory mediators TNF-α, IL-6 and CXCL-1. Prolonged KLA exposure caused dose-dependent TLR4 endocytosis that did not reverse within several hours, so a reduced surface receptor pool likely contributes to adaptation of the MyD88 branch. Receptor loss alone, however, cannot account for the memory: even after internalization of ~90% of surface TLR4, the macrophage population still discriminated the strength of a secondary stimulus. A recent systematic profiling of single-cell NF-κB activation dynamics across 80 pairwise ligand combinations at varied primary doses showed that the dose and duration of a prior inflammatory stimulus are encoded in subsequent NF-κB signaling, with both tolerance and priming arising in ligand- and dose-dependent ways (10). That work, however, varied only the primary stimulus dose against a single secondary dose, primarily across different TLR ligands. Here, we report a related but distinct form of memory: within a single TLR pathway, the amplitude of a secondary response is set by a quantitative comparison between secondary and primary stimulus strengths, and this match-or-exceed behavior extends from NF-κB dynamics to MAPK activation, IκBα degradation, and downstream cytokine and chemokine output. That this memory also governed TNF-α responses to intact, heat-inactivated gram-negative bacteria (Fig. S8) suggests it operates under physiologically relevant conditions.

Second, and central to this study, the cells’ susceptibility to IL-10 was not constitutive but was itself licensed by TLR4 stimulation history. Blocking the IL-10 receptor abolished the adaptation and memory of TNF-α and IL-6 production, whereas it left p38 and p65 phosphorylation largely unaffected, indicating that IL-10 acts downstream of the receptor-proximal kinase cascades.

Importantly, the requirement for strong prior TLR4 stimulation was not simply a consequence of greater IL-10 production following strong stimulation: titrating exogenous IL-10 to concentrations up to 100-fold above those generated by the cells themselves failed to effectively suppress TNF-α in the absence of strong priming, and, transcriptome-wide, an exogenous IL-10 concentration sufficient to suppress IL-6 altered only a small subset of genes in low-dose-primed macrophages. Consistent with IL-10 as the driver of adaptation, the switch of inflammatory signaling from low-dose sensitization to high-dose adaptation coincided with the onset of substantial IL-10 production. Direct, STAT3-mediated suppression of IL-6 thus appears to be the exception rather than a template for IL-10’s action; for most targets, including TNF-α and BCL-3, IL-10 responsiveness must be enabled by strong TLR4 signaling. This gating may have escaped notice in earlier work because IL-10’s effects on macrophages have typically been assayed with uniformly high LPS or KLA doses (31, 37, 38). The licensing we observed was graded rather than all-or-none: weakly (low-dose) primed macrophages were markedly less responsive to IL-10 than strongly primed cells, not wholly refractory to it. We also note that every condition we examined involved TLR4 engagement, our data do, thus, not address what IL-10 does to the other functions of fully naive macrophages that have never encountered a TLR4 ligand; the licensing we describe concerns the IL-10 susceptibility of the inflammatory program in TLR4-experienced cells.

Speculating on the physiological relevance of this licensing requirement, we think that several scenarios are consistent with our data, though none is directly tested here. Tissue macrophages routinely occupy microenvironments containing ambient IL-10, contributed by regulatory T cells, neighboring myeloid cells, and, at mucosal sites, steady-state epithelial microbiota-driven tone (32, 33). In such an environment, a constitutively IL-10-responsive cell would risk being silenced by this background before sensing a pathogen of its own. A second, complementary, pressure is pathogen-driven IL-10 evasion: multiple intracellular bacteria and protozoa induce host IL-10 (34), and several viruses encode IL-10 mimics that engage the host receptor (35, 36); generic IL-10 suppressibility would let such strategies silence the initial host response on encounter, whereas licensing buys the cell a response window before IL-10 can suppress it. The same gate may also help macrophage populations integrate divergent local trajectories: a resolving inflammatory focus adjacent to an expanding one, or a secondary infection superimposed on a primary one. In this setting, the rising arm could be handled by the quantitative memory described above and the falling arm by IL-10, but only once the cell has committed to a TLR response.

The most important role of IL-10 licensing may, however, be to allow for continuous evaluation of pathogen levels during an ongoing immune response. By tying a cell’s IL-10 susceptibility to its own TLR4 history, licensing lets each newly recruited macrophage respond to the pathogen levels it actually encounters, rather than being governed by cells that were activated earlier under potentially very different conditions.

What these scenarios share is the logic of an AND-gate that decouples IL-10 detection from IL-10 action until sufficient TLR engagement has occurred. Such a gate is potentially provided by the IL-10 and TLR4 stimulation history-dependent NFκB-mediated association of BCL-3 with the chromatin.

This chromatin-level mechanism for IL-10 licensing is the third major finding discussed here. An important detail of this finding is that neither the mRNA levels nor the nuclear accumulation of BCL-3, p50 or p65 predicted whether a target gene such as TNF-α would be suppressed, arguing against a simple mass-action competition among these factors. Chromatin immunoprecipitation instead revealed coordinated, IL-10-dependent changes in factor occupancy at the κB site of the TNF-α gene: after strong TLR4 history, intact IL-10 signaling promoted recruitment of BCL-3 and a reciprocal loss of p65, and blocking the IL-10 receptor reversed both – reducing promoter-associated BCL-3 by roughly two orders of magnitude while cell-wide BCL-3 abundance changed only modestly. IL-10 therefore appears to condition the p50-mediated chromatin-association competence of BCL-3 rather than its abundance, providing a candidate molecular substrate for the history-dependent licensing of IL-10 responsiveness.

An important open question is whether the strength of TLR4 stimulation further sets chromatin accessibility at these loci. Recent work has shown that an initial inflammatory stimulus reshapes the global chromatin accessibility landscape in macrophages in a dose-dependent manner (10), and a TLR4-dose-dependent priming of accessibility specifically at κB sites of inflammatory genes would mechanistically unify the KLA concentration history with IL-10 signaling within a single regulatory step.

At the single-cell level, the history dependence of TNF-α and IL-10 was encoded differently. Strong stimulation recruited essentially the entire population into Tnf expression regardless of priming, yet the per-cell abundance of Tnf transcripts varied with stimulation history – weak-then-strong stimulation generating more high-expressing cells than dual strong stimulation, and closely resembling dual strong stimulation with the IL-10 receptor blocked. For IL10 mRNA, our data showed the converse: per-cell expression was largely invariant across protocols, so that total IL-10 output was set by the fraction of producing cells. These observations reconcile the apparently paradoxical secretion data and are consistent with population-level “quorum licensing” models of macrophage activation (9, 47).

Taken together, our findings indicate that macrophages register the history of a TLR4 challenge across several layers: surface receptor availability, the activation thresholds of MAPK and NF-κB signaling, and the chromatin association of BCL-3. Central to this adaptation is the cells’ adjustable, rather than fixed, responsiveness to anti-inflammatory IL-10, which, as discussed above, may enhance the robustness of macrophage responses when faced with complex antigen- and cytokine environments.

Framing tolerance and sensitization in these quantitative terms suggests that macrophages do not simply switch between “responsive” and “hypo-responsive” states but continuously gauge whether a pathogenic stimulus is escalating or receding and tune both their inflammatory output and their susceptibility to IL-10 accordingly. The mechanisms described here were defined in vitro using BMDM and the defined TLR4 ligand KLA. Their corroboration with intact bacteria notwithstanding, it will be important to test whether the same history-dependent licensing of IL-10 operates in tissue macrophages in vivo, for example, by asking whether IL-10-rich mucosal niches selectively spare TLR4-unprimed cells from IL-10-mediated suppression.

Our experiments identify cell-intrinsic mechanisms shaping how macrophages may sense evolving pathogen loads, combining quantitative memory of TLR4 stimulation with history-gated susceptibility to IL-10. But they do not illustrate how this logic matters at the scale of an infected tissue. Our computational model is a step toward addressing this.

When IL-10 acts only on cells that have committed to a strong TLR4 response, the macrophage population tracks the direction of the pathogenic challenge, mounting a response against a rising load and resolving it as the load falls. When IL-10 instead acts on every cell in proportion to its concentration, newly recruited cells are silenced before they can read their own input. As a consequence, the information whether the pathogen load is rising or falling is lost and the pathogen may escape due to inappropriate suppression of inflammatory output. Removing IL-10 regulation altogether avoids that failure but yields unresolved, chronic inflammation. History-gated licensing escapes this trade-off rather than balancing it: by restricting IL-10’s reach to already-committed cells, it reaches the regime in which the pathogen is controlled and immunopathology is limited, satisfying within a single mechanism both demands, pathogen resistance and tolerance, that the two alternatives can only trade against each other.

The model is conceptual rather than predictive: it is dimensionless aside from a mapping of its time axis to hours, and, even though it reproduces the typical time course of an innate response, is not fitted to in-vivo infection kinetics. Its qualitative conclusions are nonetheless robust to wide parameter variation (Fig. 5C and S10) and are strengthened by the inclusion of an active resolution step (Fig. 5B). The framework also illustrates the cost of indiscriminate suppression: because pathogen escape forecloses the early clearance window, no downstream resolution program can compensate for it. This offers a functional rationale for why susceptibility to IL-10 is gated by TLR4 history rather than imposed uniformly. This framework predicts that ambient IL-10, including IL-10 homologs encoded by certain pathogens, would be considerably less effective as an immune-evasion strategy in a history-gated licensing system than in one governed solely by cytokine concentration

## Materials and Methods

### Materials

Kdo2-Lipid A (KLA) was purchased from Avanti Polar Lipids. Mek inhibitor U0126 (V1121) was obtained from Promega and re-constituted in DMSO (Sigma-Aldrich, D8418). The Alexa Fluor^™^ 647-conjugated threonine 202- and tyrosine 204-phosphorylated Erk1/2 (13148), Pacific Blue^™^-conjugated IκBα (13656), Alexa Fluor^™^ 647-conjugated serine 536-phosphorylated NF-κB p65 (4887) and PE-conjugated NF-κB1 p105/p50 (24961) antibodies were obtained from Cell Signaling Technology. PE-CF594-conjugated threonine 180- and tyrosine 182-phosphorylated p38-MAPK (563569) and PE-conjugated tyrosine 705-phosphorylated STAT3 (612569) antibodies were purchased from BD Biosciences. FITC-conjugated BCL-3 (LS-C62564) antibody was bought from LSBio. The PE-conjugated antibody for murine TLR-4 (145404) was purchased from BioLegend. anti-TNFR1 (Armenian hamster IgG, 16-1202-85), its isotype control (Armenian hamster IgG, 16-4888-85), anti-IL-10R (rat IgG1κ, 16-2101-85), its isotype control (rat IgG1κ, 144301-85), and anti-IFNAR1 (mouse IgG1κ, 16-5945-85), its isotype control (mouse IgG1κ, 14-4714-85) were from Invitrogen. Dulbecco’s modified Eagle’s medium (DMEM) was from Gibco Life Technologies. Heat-inactivated fetal bovine serum (FBS) was bought from GeminiBio.

### Preparation of Heat-inactivated Bacteria

Bacterial strain used in this study included main model *E.coli* strain K12 from ATCC (K12 MG1655). Bacteria were struck out on a Luria-Bertani (LB) agar plate and grown overnight (ON) to isolate single colonies. Single colony was frozen in a 25% glycerol stock. Bacterial culture was grown from glycerol stock with shaking at 220 rpm at 37 °C ON to saturation phase in LB broth media (KD Medical, catalog #: BLF-7030), then adjusted based on OD600 to the indicated MOI, washed 3x with PBS to clear any debris left in the media from the ON, and finally resuspended in PBS. Bacteria were heat-inactivated at 100 °C for 30 min prior to use.

### Cells and Cell Culture

Mice were maintained in specific-pathogen-free conditions at an American Association for Laboratory Animal Care-accredited animal facility at the National Institute of Allergy and Infectious Diseases (NIAID, NIH) and were used under study protocol LISB-4E approved by the NIAID Animal Care and Use Committee (National Institutes of Health, NIH).

Bone marrow cells were harvested from femurs and tibias of C57BL/6J (JAX664) mice and bone marrow progenitors were plated on non-tissue treated dishes and differentiated into BMDM during a 6-day culture in complete Dulbecco’s modified Eagle’s medium (DMEM) containing 5% FBS and supplemented with 60 ng/ml recombinant murine M-CSF (Stemcell Technologies) in a water saturated atmosphere at 37°C. For experiments, 2.5x10^6^ BMDM were seeded on non-tissue treated wells of 48 well plates in 5% FBS containing DMEM. On day of experiment, medium was replaced with fresh 5% FBS containing DMEM 30 min before experimental treatment.

### Flow cytometric analyses

FACS analysis of cell surface markers was conducted by placing cells on ice, replacing the medium with 5 mM EDTA/PBS buffer, and transferring them into a 96 well plate. Cells were blocked with 2% FBS and 0.2% goat serum containing HBSS including 1:1000 Mouse BD Fc Block^™^ (Clone 2.4G2, BDBiosciences) and stained with cell surface marker detecting fluorophore-conjugated antibodies. FACS analysis of intracellular proteins was performed by fixing cells with 2.5% PFA for 10 min at room temperature, permeabilizing in 90% methanol for 30 min at −30°C, and blocking with 2% FBS and 0.2% goat serum containing HBSS including 1:1000 Mouse BD Fc Block^™^ (Clone 2.4G2, BDBiosciences). Intracellular proteins were stained with fluorophore-conjugated antibodies overnight at 4°C. Samples were analyzed using a LSRII flow cytometer (BDBiosciences). The percent maximum value was calculated by subtraction of the baseline MFI value and division of the MFI of each sample by the maximal MFI for each protein species measured within one experiment, multiplied by 100.

### Quantitative real-time PCR

Total RNA was isolated using QiaShredder columns and the RNeasy Mini Kit (Qiagen) according to manufacturer’s instructions. RNA (100 ng) was reverse transcribed into cDNA with iScript cDNA synthesis kit (Bio-RAD) according to manufacturer’s instructions. Gene expression of murine BCL-3, cFos, cJun, CCL-2, CXCL-1. CXCL-10, IL-6, IL-10, IFN-β, Junb, TNF-α, SOCS3 and SDHA was measured with primers for murine CXCL-1 (fw: 5′- GCT TGA AGG TGT TGC CCT CAG -3′, rev: 5′- AAG CCT CGC GAC CAT TCT TG -3′), murine CXCL-10 (fw: 5′-GCC GTC ATT TTC TGC CTC AT -3′, rev: 5′- GCT TCC CTA TGG CCC TCA TT -3′), murine CCL-2 (fw: 5′- TTA AAA ACC TGG ATC GGA ACC AA -3′, rev: 5′- GCA TTA GCT TCA GAT TTA CGG GT -3′), murine IL-6 (fw: 5′-CTC TGC AAG AGA CTT CCA TCC AGT -3′, rev: 5′- GAA GTA GGG AAG GCC GTG G -3′), murine IL-10 (fw: 5′- AAG GCA GTG GAG CAG GTG AA -3′, rev: 5′- CCA GCA GAC TCA ATA CAC AC -3′), murine IFN-β (fw: 5′- AAG AGT TAC ACT GCC TTT GCC ATC -3′, rev: 5′- CAC TGT CTG CTG GTG GAG TTC ATC -3′), murine TNF-α (fw: 5′- CAT CTT CTC AAA ATT CGA GTG ACA A -3′, rev: 5′- TGG GAG TAG ACA AGG TAC AAC CC -3’), murine BCL-3 (fw: 5′- GGA GCC GCG AAG TAG ACG T - 3′, rev: 5′- TGT GGT GAT GAC AGC CAG GT -3′), murine SOCS3 (fw: 5′- GCT CCA AAA GCG AGT ACC AGC -3′, rev: 5′- AGT AGA ATC CGC TCT CCT GCA G -3′), murine JunB (fw: 5’-ATG TGC ACG AAA ATG GAA CA-3’, rev: 5’-CCT GAC CCG AAA AGT AGC TG-3’), murine cFos (fw: 5’-CGA AGG GAA CGG AAT AAG ATG-3’, rev: 5’-GCT GCC AAA ATA AAC TCC AG-3’), murine cJun (fw: 5’-AAA ACC TTG AAA GCG CAA AA-3’, rev: 5’-CGC AAC CAG TCA AGT TCT CA-3’),and murine SDHA (fw: 5’- TGG GGA GTG CCG TGG TGT CA - 3’, rev: 5’- GTG CCG TCC CCT GTG CTG GT -3’). Real-time PCR was performed using Fast SYBR^™^ Green Master Mix (ThermoFisher Scientific, USA) according to manufacturer’s instructions in MicroAmp^™^ Fast Optical 96-Well Reaction Plates ThermoFisher Scientific, 4346906) with a QuantStudio^™^ 6 Flex Real-Time PCR System (ThermoFisher Scientific, 4485691). Quantification of gene expression was calculated as described by Pfaffl et al. (56).

### Immunocytochemistry and widefield fluorescence microscopy

For widefield fluorescence microscopy, 1x10^5^ BMDM cells were seeded on Falcon 96 well Flat Bottom TC-treated Imaging Microplate (Corning, 353219) and cultivated for 24h before experimental treatment. After treatment, cells were fixed with 2.5% PFA for 10 min at room temperature. Cells were then permeabilized in 70% ethanol for 30 min. After permeabilization, cells were blocked with 2% FBS and 0.2% goat serum containing HBSS including 1:1000 Mouse BD Fc Block^™^ (Clone 2.4G2, BDBiosciences). For detection of endogenous p65, BCL-3 and p50, cells were stained with antibodies specific for p65, BCL-3 and p50 overnight at 4°C. Additionally, DAPI (BioLegend) was added for nuclear staining. Samples were imaged using a CellInsight CX7 Pro HCS Platform (ThermoFisher Scientific) equipped with an 40x objective lens. Quantification of the nuclear localization of Alexa Fluor-647-labeled p65, FITC-labeled BCL-3 and PE-labeled p50 were performed by using CellProfiler^™^ for determining the total intensity of fluorophores inside the nucleus, and the relative ratio of intensity of the fluorophores in the nucleus and in the cytoplasm. Nuclear to cytoplasmic ratio was calculated as the nuclear localized fluorophore intensity divided by the intensity of the fluorophore in the cytoplasm.

### Cytokine release quantification

For assessing cyto- and chemokine release, supernatant of treated BMDM was taken after putting cells on ice. Supernatant was frozen at −80°C for long-term preservation. After thawing, supernatant was prepared and analyzed with the LegendPlex^™^ Multiplex Assay Kits Mouse Proinflammatory Chemokine Panel (740451) and Mouse Inflammation Panel (740446) according to manufacturer’s instructions by using a LSRII flow cytometer (BDBiosciences). Raw data was further processed with LegendPlex^™^ Desktop software.

### Chromatin immunoprecipitation (ChIP) assay

Samples for ChIP assays and sequencing from in vitro cultured BMDM have been prepared according to Rousselet (57). After fixation of adherent cells on a 10 cm dish in 1% paraformaldehyde and adding glycine to a final concentration of 125 mM, cells were washed with PBS containing phosphatase and protease inhibitor cocktail Halt^™^ (1:500) (ThermoFisher Scientific, 78440) ,transferred in a 15 ml tube and centrifuged for 8 min at 300x g and 4 °C. Pellet was resuspended in SDS-containing lysis buffer and lysates have been passed through a 27Gx1/2 needle to ensure separation of nuclei. Lysates were sonicated with a BioRuptor UCD-300 (Diagenode) for 13 cycles of 30 s on/30 s off, respectively. This was repeated 3 times. To check for the extent of chromatin shearing, 10% of the sample were transferred in a new tube and treated with Proteinase K and RNase and were reverse crosslinked. DNA was then extracted with phenol/chloroform/isoamylalcohol and precipitated with ethanol. DNA was size fractionated via gel electrophoresis on a 1% agarose gel. Successful chromatin fragmentation resulted in a smear of DNA fragments ranging from 100-600 bp. Fragmented chromatin has been pelleted and resuspended in ChIP dilution buffer (see (57)). Samples were pre-cleared with Pierce^™^ ChIP-grade Protein A/G conjugated magnetic beads (ThermoFisher Scientific, 26162). Beads were removed using a magnetic tube rack and supernatants containing the pre-cleared chromatin were splitted into immunoprecipitation (IP) and input samples. Supernatants of each sample that were used for IP, have been incubated with 10 μg of either anti-BCL3 (SantaCruz, sc-32741) or it’s corresponding isotype control antibody (Armenian Hamster IgG, ThermoFisher Scientific, 16-4888-85) overnight at 4 °C. p50 and p65 IP have been performed with anti-p50 (Santa Cruz, sc-8414X) or anti-p65 (Santa Cruz, sc-8008X) antibodies or their respective isotype control antibody (Mouse IgG1 kappa, ThermoFisher Scientific, 14-4714-85). The next day, in 1.5% fish skin gelatin (Sigma-Aldrich, G7041) and glycogen (Sigma-Aldrich, G8751) pre-blocked Pierce^™^ ChIP-grade Protein A/G conjugated magnetic beads (ThermoFisher Scientific, 26162) were added to IP samples to bind chromatin/antibody complexes and incubated for 2 h at 4 °C. Tubes were placed in a magnetic tube rack, liquid was removed and beads were washed with a succession of buffers according to Rousselet (57). Chromatin/antibody complexes were eluted from the beads for 30 min. IP samples and Input samples were reverse crosslinked over night at 65°C in a rotary shaker. After adding Proteinase K and RNase, samples were incubated for 1 h at 37 °C. DNA was purified using Qiaquick^®^ PCR Purification Kit (Qiagen, 28104).

For analysis using quantitative real-time PCR, IP DNA sample and input sample were used as templates. Real-time PCR analysis of DNA samples was performed according to procedure described in section ‘Quantitative real-time PCR’ using primer for known BCL3 specific κB-sites in the murine TNF-α gene (fw: 5’-CCA GGA GGG AGA ACA GAA ACT C-3’ , rev: 5’- CAC AAG CAG GAA TGA GAA GAG G -3’) (31), murine IL-6 gene (fw: 5’- GAC ATG CTC AAG TGC TGA GTC AC -3’, rev: 5’- AGA TTG CAC AAT GTG ACG TCG -3’) (58) and murine IL-10 gene (fw: 5’- TAG AAG AGG GAG GAG GAG CC -3’, rev: 5’- TGT GGC TTT GGT AGT GC AAG -3’) (59). Data was analyzed and is given as ratio (fold enrichment) comparing the amount of the target sequence measured in the ChIP isolate to the amount measured in the isotype control isolate.

### Bulk RNA-seq

For bulk RNA sequencing, total RNA from BMDM was isolated using QiaShredder columns and the RNeasy Mini Kit (Qiagen) according to manufacturer’s instructions. Before eluting isolated RNA from RNeasy spin columns, bound RNA/DNA has been incubated with DNase I (RNase-Free DNase Set, Qiagen) to remove DNA from samples. After RNA elution, RNA quantity and integrity was assessed by the Bioanalyzer (Agilent). 100 ng of total RNA was used in conjunction with the TruSeq^®^ Stranded Total RNA Library Prep kit (Illumina). The libraries quality was checked by the Bioanalyzer and quantitated by the Qubit (ThermoFisher Scientific). Equimolar quantities from each sample library were pooled and sequenced on a High throughput Next-Seq2000 instrument.

### RNA sequencing data analysis

Paired-end sequence files (.fastq) per sample were quality inspected using the FastQC tool 0.12.1 (https://www.bioinformatics.babraham.ac.uk/projects/fastqc/) Reference mapping of each fastq-file has been performed with the Rhisat2 package (https://bioconductor.org/packages/release/bioc/html/Rhisat2.html) in R using the mouse reference genome GRCm38 (https://cloud.biohpc.swmed.edu/index.php/s/grcm38/download).

Gene expression has been quantified after annotation of mapped genes from the saf file provided by https://bioinf.wehi.edu.au/Rsubread/annot/ and after counting reads to genes from the genomic alignment with the Rsubread package (https://bioconductor.org/packages/release/bioc/html/Rsubread.html ) in R. Counts have been Z-score normalized. For differential gene expression (DEG) analysis, genes with less than 10 reads have been excluded. For genes not discarded, expression differences across sample classes were tested using the DESeq2 (https://bioconductor.org/packages/release/bioc/html/DESeq2.html) package in R.

### Single cell RNA sequencing

Single cell suspensions were collected into PBS+0.5% BSA. Single cell suspensions quality, number and viability were assessed with a dual fluorescence cell counter LUNA-FX7TM (Logos Biosystems). 3,000 cells were targeted from each sample cell suspension. The cells were washed twice with PBS+0.04% BSA and resuspended in about 500 cells per microliter.

10X Genomics’ Chromium instrument and Dual index Single-Cell 3′ Reagent kit (V3.1) were used to prepare the individually barcoded single-cell RNA-Seq libraries following the manufacturer’s protocol. Library qualities were assessed by the TapeStation-4200 traces (Agilent BioAnalyzer High Sensitivity Kit) and quantitated by the Qubit system (ThermoFisher Scientific). Sequencing was done on the Illumina NextSeq-2000 machine, using the P3 kit. Following sequencing, an average 50,000 reads per cell were generated. The bcl files were demultiplexed into a FASTQ, aligned to Mouse transcriptome mm10-2020-A and single-cell 3′ gene counting were performed by the standard 10X Genomics’ CellRanger mkfastq software (V8.0.0).

The single-cell QC and the 10X Genomics’ H5 output files, containing the gene counts per cell matrix were generated by 10X Genomics’ CellRanger Downstream analysis utilized a Python-based pipeline that primarily relied on Scanpy (v1.9.8) for single cell dataset processing. Briefly, H5 count matrices from all samples were simultaneously loaded in using Anndata, and duplicate indices across experiments were given unique names. Mitochondrial-derived genes were identified and used for standard Scanpy quality control checks. After confirming satisfactory quality of the experimental data, the count matrix was filtered to exclude cells that expressed less than 100 genes and genes which were expressed in fewer than 3 cells. This filtered matrix was then subject to normalization using the Scanpy normalize_total function, which normalizes the total counts per cell across the dataset. This normalized matrix was then scaled using a log+1 function, and these filtered, normalized, and scaled matrices were lastly further filtered to only include the 20,000 most highly variable genes. These final matrices were then used for all downstream analyses.

After this standard Scanpy pipeline, analysis was done using a custom script. UMAP plots were created using the Scanpy (v1.9.8) package, with figures generated using matplotlib. Expression node figures were created in Adobe Illustrator based on a simple binary determination of cellular gene expression. Counts of cells expressing each given gene were recorded, while simultaneously keeping track of cellular identity to determine single, double, triple, and quadruple positive cells for given marker genes.

### Standard statistical analyses

Statistical analyses were performed with R (version 4.3.0) and RStudio (version 2023.03.1) for MacOS. Data were tested for normal distribution with the Shapiro-Wilk test and homoscedasticity using Levene test. In case of non-normally distributed or heteroskedastic data, nonparametric tests such as Mann–Whitney-U (for single comparison) and Kruskal–Wallis (followed by post hoc Dunn–Bonferroni for multiple comparisons) were applied. For normally distributed data, parametric tests such as *t*-test (for single comparison) and ANOVA (followed by post hoc Tukey for multiple comparisons) were performed. The designations * p < .05, ** p < .01, *** p < .001 denote p-values for the measured differences. If no p-value is indicated, the comparison is considered non-significant. All experiments contained a minimum of three replicates (n = 3).

## Supplementary Material

1

## Figures and Tables

**Table 1.** Gene-expression clusters defined by TLR4 dose and IL-10 dependence.

Cluster	Dependence on KLA dose	Influence of IL-10	Representative genes

C1	Highest in resting cells, decreases with increasing KLA	Mostly IL-10 independent	Cd300lb, Casp2, Cxcr4, Mafb, Dusp6
C2	Induced by KLA; adapts to high primary dose	Adaptation requires IL-10	Tnf, Ccl4, Irf1, Icam1, Il10ra, H2-M2
C3	Induced by KLA; strongest after high primary dose	Induction requires IL-10	Bcl3, Il19, Dusp7, Sema4c, Timp1, Mt1, Mt2
C4	Induced by strong KLA	Strongly suppressed by IL-10	Nfkb1, Nfkb2, Nfkbib, Il6, Il12b, Il27, Cxcl10, Socs, IL10, IL12b, Ccl2
C5	Increases with KLA dose	Less strongly suppressed by IL-10	Gbp3, Nos2, Ccl5, Jak2, Fpr2, C3
