# Supplementary material for: Macrophages tune responses to pathogen dynamics through TLR4 stimulation memory and by licensing susceptibility to IL-10
Source: bioRxiv. 2026 Jul 9:2024.03.28.587272. Originally published 2024 Mar 31. Preprint. [Version 3] doi: 10.1101/2024.03.28.587272 (PMC11037870; doi:10.1101/2024.03.28.587272)
Supplement: 1 [file NIHPP2024.03.28.587272v3-supplement-1.pdf]

bioRxiv preprint doi: https://doi.org/10.1101/2024.03.28.587272; this version posted July 9, 2026. The copyright holder for this preprint (which was not certified by peer review) is the author/funder. This article is a US Government work. It is not subject to copyright under 17 USC 105 and is also made available for use under a CC0 license.

Fig. S1: Blocking IL-10 does not affect AP-1 family mRNA expression in TLR4 ligand pre-exposed and re-stimulated macrophages.

BMDMs were incubated with 5% FBS DMEM containing either IL-10R blocking antibody or its isotype control. During primary stimulation, cells were stimulated with 0, 0.1, 1 or 100 nM KLA. After 4 hrs, cells were washed, and medium was replaced with 5% FBS DMEM containing either IL-10R blocking antibody or its isotype control and cells were incubated for 1 hr. Subsequently, re-stimulation was performed using 0 or 100 nM KLA. After 1 hr, total RNA was isolated and subjected to qRT-PCR analysis to monitor (A) cFos, (B) JunB and (C) cJun mRNA expression. The expression of aforementioned mRNAs was normalized to SDHA mRNA expression. Relative expression of mRNA is given in fold of mRNA expression in naïve and unstimulated control cells (fold of control (FOC)) as described by Pfaffl et al. (*56*). Each replicate was performed with BMDMs from different mice and data sets include 4 replicates (n=4). Kruskal–Wallis test with post-hoc Dunn-Bonferroni comparisons: *$p < .05$, **$p < .01$, ***$p < .001$, ****$p < .0001$.

bioRxiv preprint doi: https://doi.org/10.1101/2024.03.28.587272; this version posted July 9, 2026. The copyright holder for this preprint (which was not certified by peer review) is the author/funder. This article is a US Government work. It is not subject to copyright under 17 USC 105 and is also made available for use under a CC0 license.

Fig. S2: Low dose KLA priming dependent increase in restimulation induced cyto- and chemokine release is accompanied by reduced IL-10 expression.

BMDMs were incubated with 5% FBS DMEM containing either IL-10R blocking antibody or its isotype control. During primary stimulation, cells were treated with 0, 0.03, 0.1, 0.3, 1, or 100 nM KLA. After 4 hrs, cells were washed, and medium was replaced with 5% FBS DMEM containing either IL-10R blocking antibody or its isotype control and cells were incubated for 1 hr. Subsequently, restimulation was performed using 0, 1 or 100 nM KLA. After 3 hrs, supernatants were collected, processed with the LegendPlex™ Multiplex Assay Kits and cyto- and chemokine levels for (A) TNF-α, (B) IL-6, (C) CXCL-1, (D) IFN-β, (E) IL-10, (F) CCL-2, (G) CCL-4, (H) CCL-5, (I) CXCL-10, and (J) IL-12p70 were determined by using a LSRII flow cytometer (BDBiosciences). Raw data was processed with LegendPlex™ Desktop software. Each replicate was performed with BMDMs from different mice and data sets include 6 replicates (n=6). Data was normalized to the maximal cytokine secretion (set as 100%). Kruskal–Wallis test with post-hoc Dunn-Bonferroni comparisons: *$p < .05$, **$p < .01$, ***$p < .001$, ****$p < .0001$.

Fig. S3: High dose KLA primary stimulation induces hyporesponsive behavior of cytokine and chemokine release in response to restimulation while blocking IL-10 reverses hyporesponsiveness.

BMDMs were incubated with 5% FBS DMEM containing either IL-10R blocking antibody or its isotype control. During primary stimulation, cells were treated with 0, 1, 10 or 100 nM KLA. After 4 hrs, cells were washed, and medium was replaced with 5% FBS DMEM containing either IL-10R blocking antibody or its isotype control and cells were incubated for 1 hr. Subsequently, restimulation was performed using 0, 1, 10 or 100 nM KLA. After 3 hrs, supernatants were

bioRxiv preprint doi: https://doi.org/10.1101/2024.03.28.587272; this version posted July 9, 2026. The copyright holder for this preprint (which was not certified by peer review) is the author/funder. This article is a US Government work. It is not subject to copyright under 17 USC 105 and is also made available for use under a CC0 license.

collected, processed with the LegendPlex™ Multiplex Assay Kits and cyto- and chemokine levels for (A) TNF-α, (B) IL-6, (C) CXCL-1, (D) IFN-β, (E) IL-10, (F) CCL-2, (G) CCL-4, (H) CCL-5, (I) CXCL-10, and (J) IL-12p70 were determined by using a LSRII flow cytometer (BDBiosciences). Raw data was processed with LegendPlex™ Desktop software. Each replicate was performed with BMDMs from different mice and data sets include at least 5 replicates (n=5). Kruskal–Wallis test with post-hoc Dunn-Bonferroni comparisons: *$p < .05$, **$p < .01$, ***$p < .001$, ****$p < .0001$.

Fig. S4: KLA concentrations during primary stimulations affect RNA production of cytokines and chemokines upon restimulation. Blocking IL-10 reverses hypo-responsiveness. BMDMs were incubated with 5% FBS DMEM containing either IL-10R blocking antibody or its isotype control. During primary stimulation, cells were treated with 0, 0.1, 1 or 100 nM KLA. After 4 hrs, cells were washed, and medium was replaced with 5% FBS DMEM containing either IL-10R blocking antibody or its isotype control and cells were incubated for 1 hr. Subsequently, re-stimulation was performed using 0 or 100 nM KLA. After 1 hr, total RNA was isolated and subjected to qRT-PCR analysis to monitor (A) TNF-α, (B) IL-6 (C) IL-10, (D) IFN-β, (E) CCL-2, (F) CXCL-1, (G) CCL-4, (H) CCL-5 and (I) CXCL-10 mRNA expression. The expression of aforementioned mRNAs was normalized to SDHA mRNA expression. Relative expression of mRNA is given in fold of mRNA expression in naïve and unstimulated control cells (fold of control (FOC)) as described by Pfaffl et al. (*56*). Each replicate was performed with BMDMs from different mice and data sets include at least 5 replicates (n=5). Kruskal–Wallis test with post-hoc Dunn-Bonferroni comparisons: *$p < .05$, **$p < .01$, ***$p < .001$, ****$p < .0001$.

bioRxiv preprint doi: https://doi.org/10.1101/2024.03.28.587272; this version posted July 9, 2026. The copyright holder for this preprint (which was not certified by peer review) is the author/funder. This article is a US Government work. It is not subject to copyright under 17 USC 105 and is also made available for use under a CC0 license.

Fig. S5: IL-10 without strong prestimulation is insufficient to induce hyporesponsiveness.

BMDMs were incubated with 5% FBS DMEM. During primary stimulation, cells were treated with 0, 0.1 or 100 nM KLA. 20 minutes later, recombinant murine IL-10 was added in depicted concentrations. After 4 hrs, cells were washed, medium was replaced with 5% FBS DMEM and cells were incubated for 1 hr. Subsequently, restimulation was performed using 0 or 100 nM KLA. 20 minutes later, recombinant murine IL-10 was added in depicted concentrations. After 3 hrs, supernatants were collected, processed with the LegendPlex™ Multiplex Assay Kits and cytokine and chemokine levels for (A) IFN-b, (B) CXCL-1, (C) CCL-2, (D) CCL-4, (E) CCL-5 , (F) CXCL-10, and (G) IL-12p70 were determined by using a LSRII flow cytometer (BDBiosciences). Raw data was processed with LegendPlex™ Desktop software. Each replicate was performed with BMDMs from different mice and data sets include 6 replicates (n=6). Data are given as mean ± standard error of mean. Kruskal–Wallis test with post-hoc Dunn-Bonferroni comparisons: *$p < .05$, **$p < .01$, ***$p < .001$, ****$p < .0001$.

(H) Recombinant IL-10 induces STAT3 phosphorylation.

BMDMs were incubated with 5% FBS DMEM. During primary stimulation, cells were treated with 0, 0.1 or 100 nM KLA. 20 minutes later, recombinant murine IL-10 was added in depicted concentrations. After 4 hrs, cells were washed, medium was replaced with 5% FBS DMEM and cells were incubated for 1 hr. Subsequently, restimulation was performed using 0 or 100 nM KLA. 20 minutes later, recombinant murine IL-10 was added in depicted concentrations for 20 minutes. Subsequently, cells were washed, fixed, permeabilized and intracellularly stained for phosphorylated STAT3. Median fluorescence intensity of samples was determined by using a LSRII flow cytometer (BDBiosciences). Raw data was processed with FlowJo™. Data is given in

bioRxiv preprint doi: https://doi.org/10.1101/2024.03.28.587272; this version posted July 9, 2026. The copyright holder for this preprint (which was not certified by peer review) is the author/funder. This article is a US Government work. It is not subject to copyright under 17 USC 105 and is also made available for use under a CC0 license.

% of maximal median fluorescence intensity within each replicate (set as 100%) and was normalized by subtracting median fluorescence intensity of the sample with the detected minimal median fluorescence intensity (set as 0%). Each replicate was performed with BMDMs from different mice and data set includes 3 replicates (n=3). Data are given as mean ± standard error of mean. Kruskal–Wallis test with post-hoc Dunn-Bonferroni comparisons of each sample with unstimulated control (planned comparisons): *$p < .05$, **$p < .01$, ***$p < .001$, ****$p < .0001$.

Fig. S6: Fluorescence microscopy images of p65, p50 and BCL-3 localization and ratio nuclear vs. cytoplasmic localization

(A) BMDMs were incubated with 5% FBS DMEM containing either IL-10R blocking antibody or its isotype control. During primary stimulation, cells were treated with 0, 0.1, 1 or 100 nM KLA. After 4 hrs, cells were washed, and medium was replaced with 5% FBS DMEM containing either IL-10R blocking antibody or its isotype control and cells were incubated for 1 hr. Subsequently, restimulation was performed using 0, 1 or 100 nM KLA. After 20 mins, cells were washed, fixed, permeabilized and intracellularly stained for p65, p50, and BCL-3, as well as stained with DAPI, and images were acquired using the CellInsight CX7 Pro HCS Platform (Thermo Fisher Scientific) equipped with an 40x objective lens. (B-G) Raw data was processed with CellProfiler™. Data is given in relative mean nuclear intensity as % of maximal nuclear fluorescence intensity within each replicate (set as 100%) (B-D) or nuclear to cytoplasmic ratio (E-G) of the corresponding fluorophore-coupled antibody for p65, p50 or BCL-3. Each replicate was performed with BMDMs from different mice and data sets include at least 6 replicates (n=6). Kruskal–Wallis test with post-hoc Dunn-Bonferroni comparisons: *$p < .05$, **$p < .01$, ***$p < .001$, ****$p < .0001$.

bioRxiv preprint doi: https://doi.org/10.1101/2024.03.28.587272; this version posted July 9, 2026. The copyright holder for this preprint (which was not certified by peer review) is the author/funder. This article is a US Government work. It is not subject to copyright under 17 USC 105 and is also made available for use under a CC0 license.

Fig. S7: Single cell sequencing of Tnf and Il10 gene expression

BMDM were incubated with 5% FBS DMEM containing either IL-10R blocking antibody or its isotype control. During priming, cells were stimulated with 0, 0.1, or 100 nM KLA. After 4 hrs, cells were washed, and medium was replaced with 5% FBS DMEM containing either IL-10R blocking antibody or its isotype control and cells were incubated for 1 hr. Subsequently, re-stimulation was performed using 0 or 100 nM KLA. After 1 hr, cells were collected and subjected to single cell RNA sequencing. Pre-processed data was used for unsupervised UMAP clustering of all treatment conditions. Each dot represents one cell either negative (grey) or positive (color) for the respective expressed gene. Percent of expressing and non-expressing cells depicted as pie charts.

Fig. S8: Bacterial load during 1st challenge sets TNF-α and IL-6 threshold bacterial load for increased responsiveness toward 2nd challenge.

During primary and secondary stimulation, BMDMs were incubated with 5% FBS DMEM. During the 1stchallenge, cells were stimulated with 0, 0.01, 0.1,1, 10, and 100 MOI of heat-inactivated K12 *E.coli*. Restimulation was performed using 0, 0.01, 0.1,1, 10, and 100 nM MOI of heat-inactivated K12 *E.coli*. After 3 hrs, supernatants were collected, processed with the LegendPlex™ Multiplex Assay Kits and cytokine and chemokine levels of (A) TNF-α, (B) IL-6, (C) IL-10, (D) IFN-β, (E) CCL-2, and (F) IL-12p70 were determined. Raw data was processed with LegendPlex™ Desktop software. Each replicate was performed with BMDMs from different mice. Data include at least 6 replicates (n=6). Data was normalized to the mean maximal cytokine secretion induced by restimulation in naïve (0 MOI primary challenge) cells (set as 100%). Data are given as mean ± SEM. P-values: Kruskal–Wallis test with post-hoc Dunn-Bonferroni

bioRxiv preprint doi: https://doi.org/10.1101/2024.03.28.587272; this version posted July 9, 2026. The copyright holder for this preprint (which was not certified by peer review) is the author/funder. This article is a US Government work. It is not subject to copyright under 17 USC 105 and is also made available for use under a CC0 license.

comparisons. In (A), Dark red-colored triangles attached to blue curve (1st challenge = 10 MOI) indicate the increase in TNF-α secretion following 2nd stimulation for every 10-fold increase in bacterial load of the secondary challenge. The biggest increase can be seen between the 2nd challenges with 1 and 10 MOI. Similarly, for a 1st challenge 0.1 or 1 MOI, the strongest changes in the responses can be seen for 2nd challenges between 0.01 and 0.1 or 0.1 and 1 MOI, respectively. The same behavior (strongest increases following secondary stimulation matching the first) was observed for IL-6. Stimulation with a MOI of 100 resulted in persistent production of TNF-α and IL-6 and hypo-responsiveness toward secondary challenge.

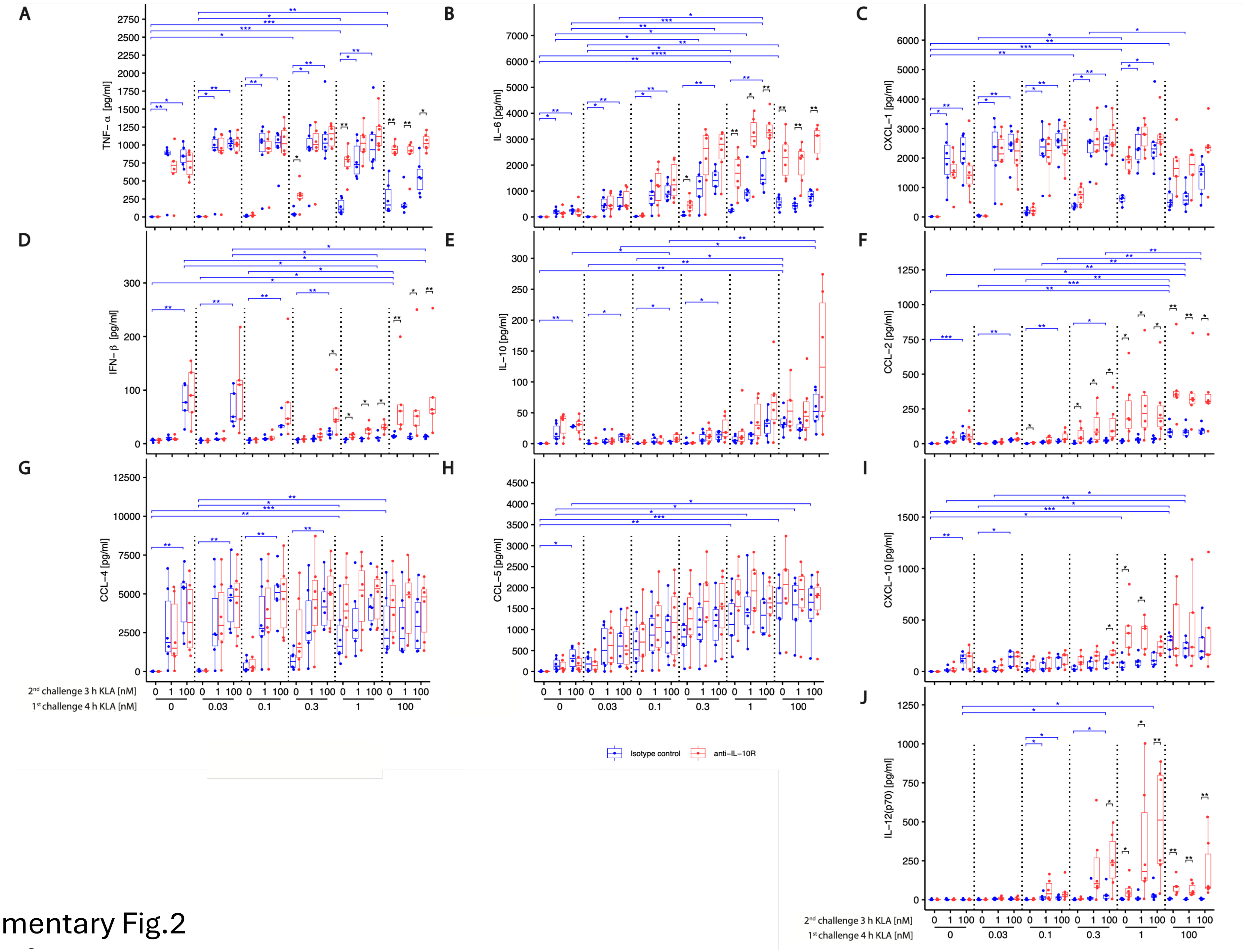

Supplementary Fig.2

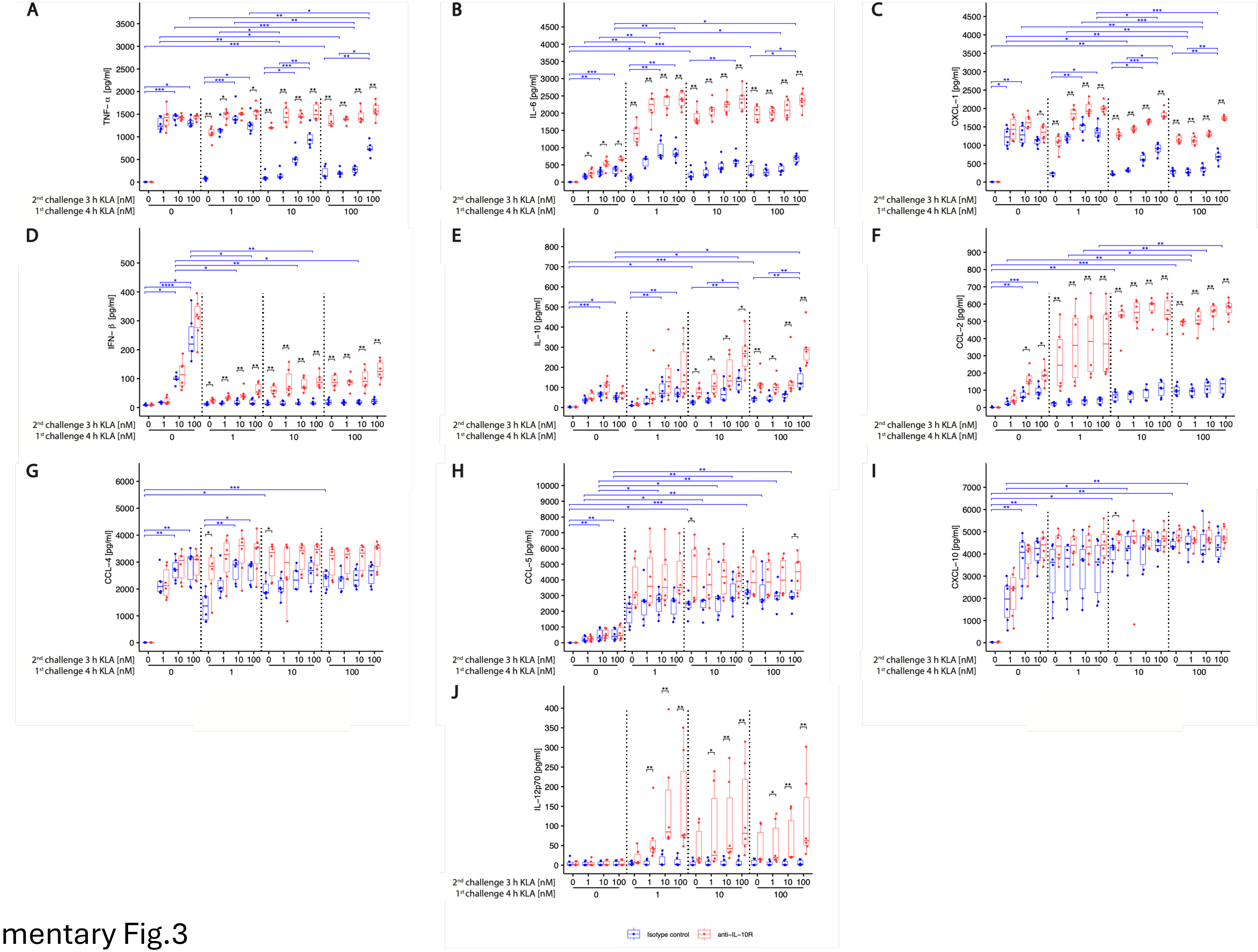

Supplementary Fig.3

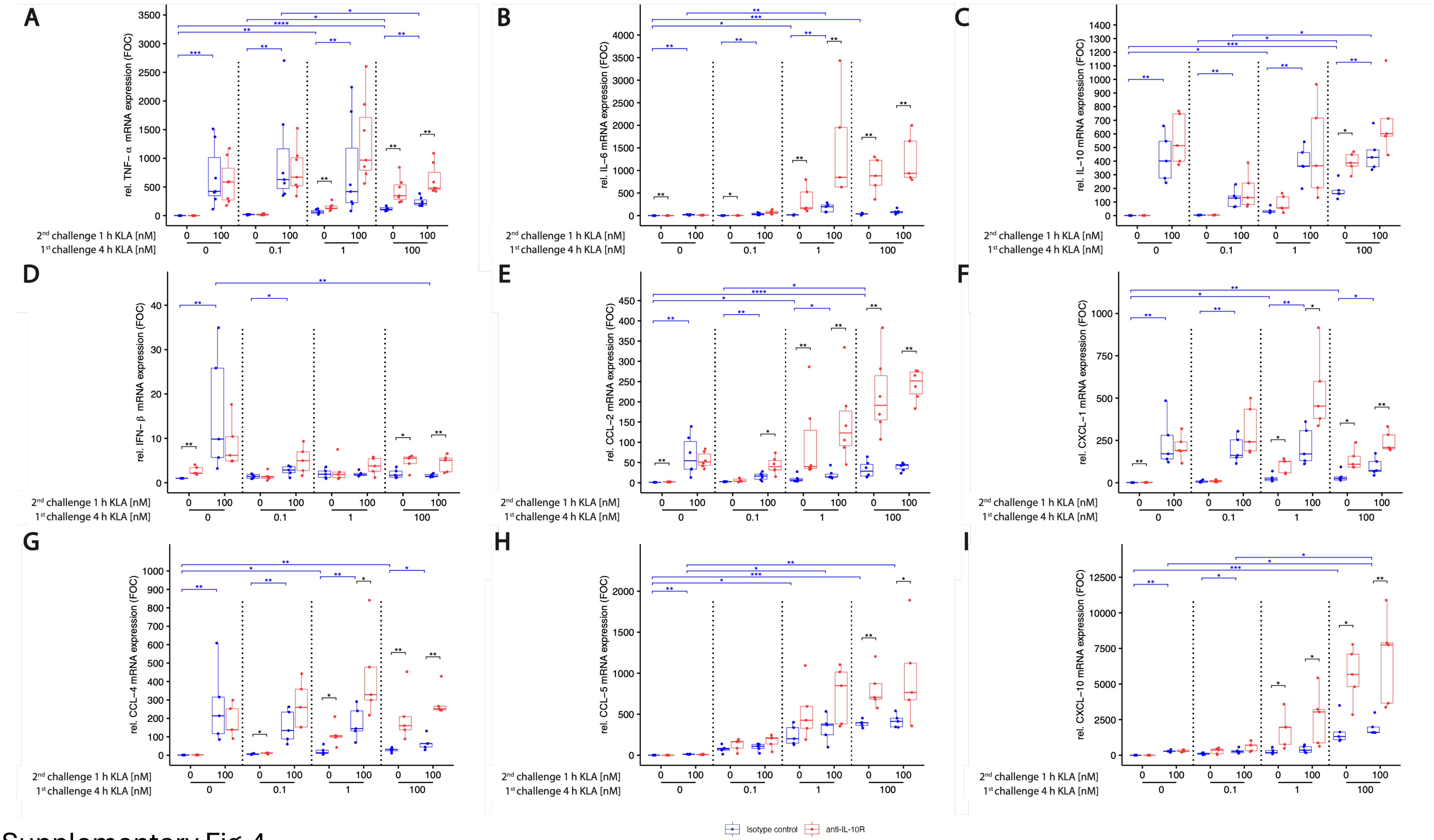

Supplementary Fig.4

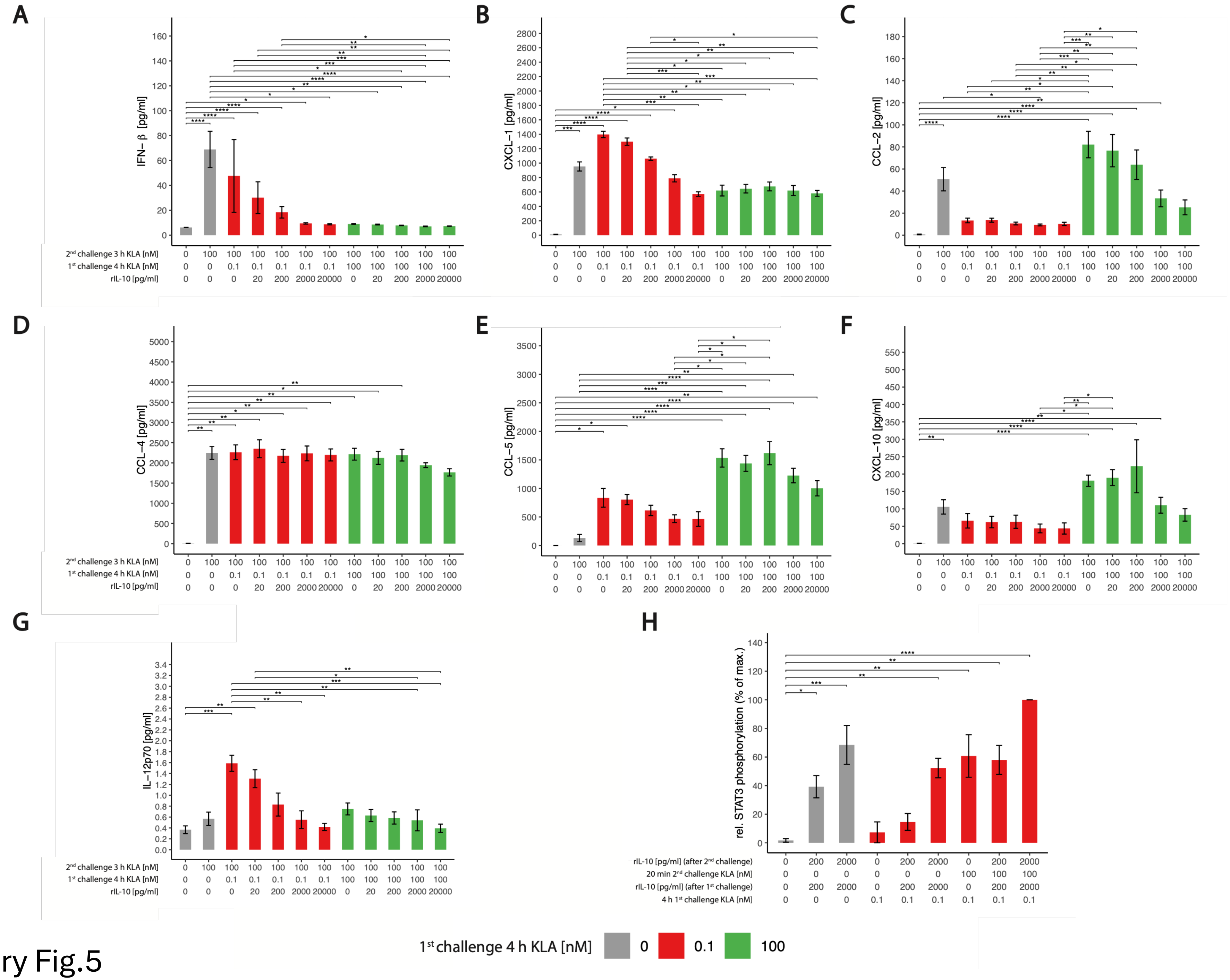

Supplementary Fig.5

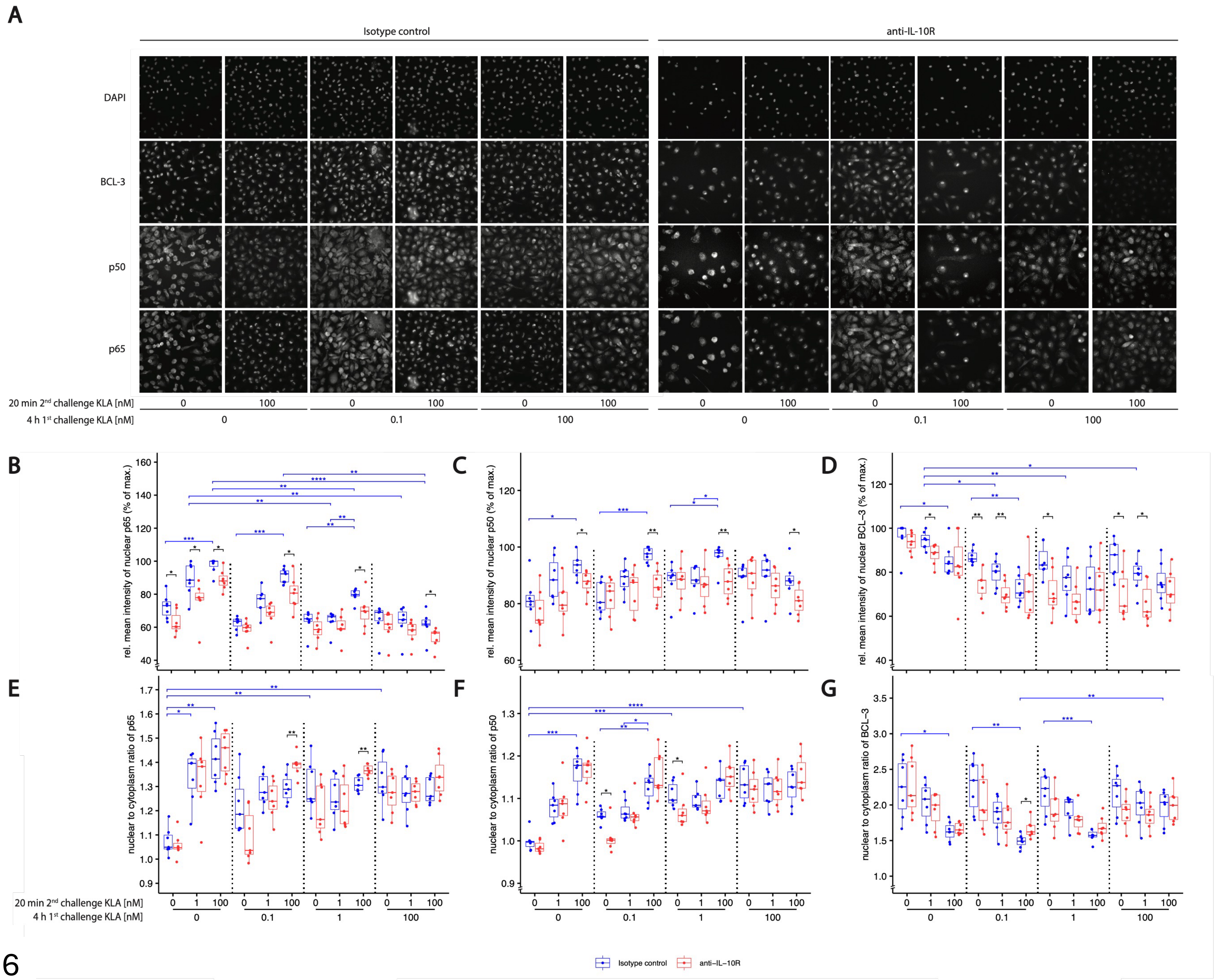

Supplementary Fig.6

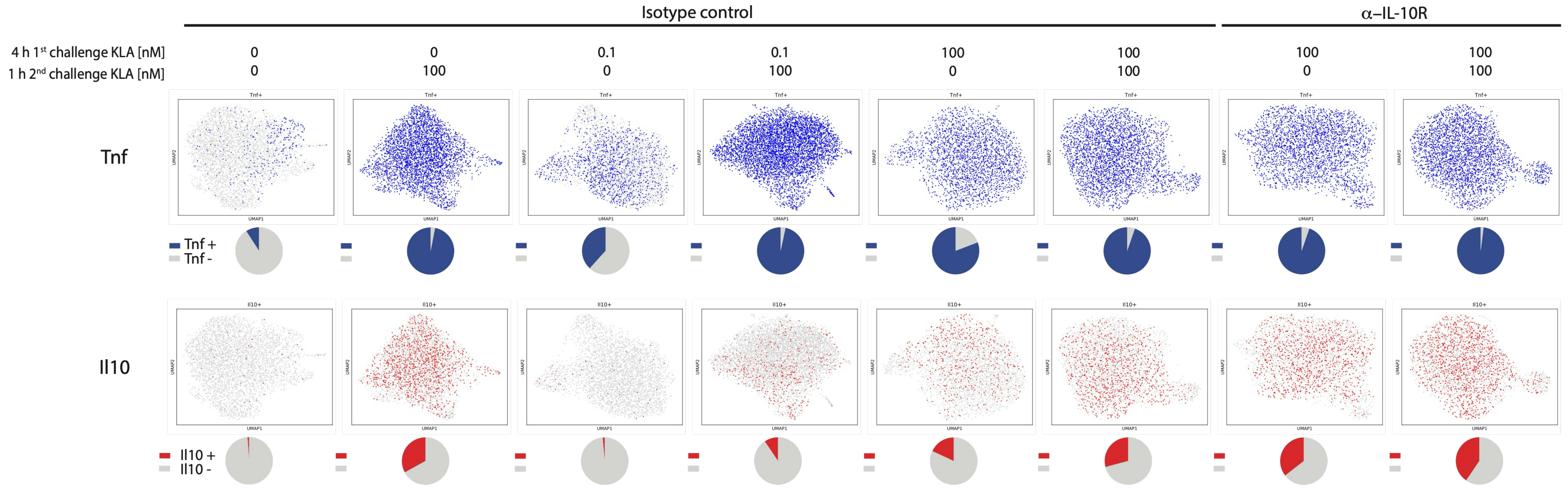

Supplementary Fig.7

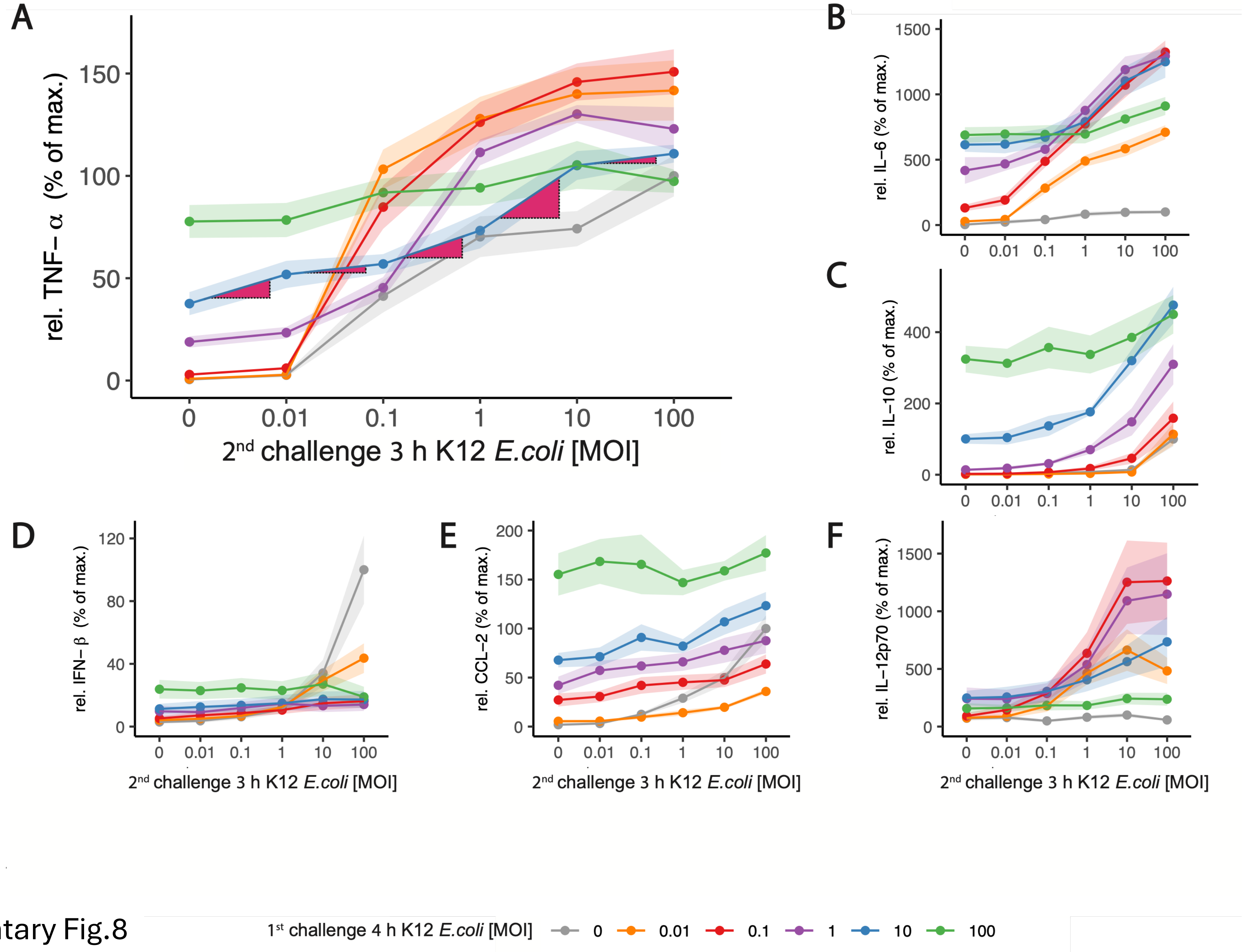

Supplementary Fig.8

bioRxiv preprint doi: https://doi.org/10.1101/2024.03.28.587272; this version posted July 9, 2026. The copyright holder for this preprint (which was not certified by peer review) is the author/funder. This article is a US Government work. It is not subject to copyright under 17 USC 105 and is also made available for use under a CC0 license.

Supplementary Text 1: A minimal model of IL-10 licensing and TLR4 memory during a macrophage response to infection

1. Model purpose and scope

This model illustrates the functional consequence of two coupled features of macrophage regulation, namely history-dependent IL-10 licensing and quantitative (match-or-exceed) memory, over the course of a single infection. It is deliberately minimal and dimensionless: it is not fitted to kinetic data, but is used to compare three regulatory logics under identical parameters, so that any difference in outcome is attributable to the single per-cell term by which IL-10 acts on inflammatory output.

2. State variables

To encode the stimulation-specific memory of macrophages, the model uses a series of $K + 1$ hierarchical bins (rungs), representing geometrically spaced levels of TLR4 experience. So the base model (with $K = 12$) integrates 13 macrophage states plus four scalar entities. The resolution extension adds a transition of to an M2-like state (Section 10):

- $P(t)$ — pathogen load, normalized to its carrying capacity K_P;
- $M_j(t),\ j = 0, \dots, K$ — macrophages occupying memory rung j (a discretized adaptation set-point, see below), with M_0 the naive cells;
- $E(t)$ — lumped pro-inflammatory output (a TNF-α/IL-6 proxy) that drives pathogen clearance;
- $R(t)$ — IL-10;
- $D(t)$ — tissue-damage proxy;
- $M2(t)$ — pro-resolving (M2) macrophages (added in the extension, Section 10).

3. Sensing and history variables

Sensing drive. All cells sense the same drive $d(t)$, a saturating sum of a pathogen term and a sterile damage-associated (DAMP) term, so that released damage re-activates the same TLR4-type pathway. The drive is a dynamic quantity computed from the current state (it is not a free parameter and does not appear in the parameter Table 1); it enters the escape gate, the inflammatory output, and IL-10 production below:

$$d(t) = \frac{P}{P + K_s} + w_D \frac{D}{D + K_D}. \quad (1)$$

IL-10 suppression factor. The fraction of pro-inflammatory output that remains under the current IL-10 level (a Hill function), with $f = 1$ when IL-10 is absent or its receptor is blocked and $f \to 0$ at saturating IL-10:

$$f(R) = \frac{K_R^n}{K_R^n + R^n} \in [0, 1]. \quad (2)$$

bioRxiv preprint doi: https://doi.org/10.1101/2024.03.28.587272; this version posted July 9, 2026. The copyright holder for this preprint (which was not certified by peer review) is the author/funder. This article is a US Government work. It is not subject to copyright under 17 USC 105 and is also made available for use under a CC0 license.

Memory set-points (the ladder). Cells carry a discretized adaptation set-point on a fixed geometric ladder spanning the sensing dynamic range; the ladder positions are set by that range, not fitted:

$$\theta_0 = 0, \qquad \theta_j = \theta_{min}\left(\frac{\theta_{max}}{\theta_{min}}\right)^{\frac{j-1}{K-1}}, \quad j = 1, \ldots, K. \tag{3}$$

Licensing. A cell's susceptibility to IL-10 is a smooth (graded) function of its memory set-point: only cells with a strong stimulation history are IL-10-suppressible:

$$\ell_j = \frac{\theta_j^m}{\theta_j^m + \theta_{1/2}^m} \in [0, 1]. \tag{4}$$

Escape (match-or-exceed). A stimulus that exceeds a cell's set-point re-activates it. This soft threshold also drives the upward ratchet of the ladder:

$$\sigma_j = \frac{1}{1 + \exp[-(d - \theta_j)/\varepsilon]}. \tag{5}$$

4. Dynamics

Macrophage recruitment and memory ratchet. Cells are recruited at the lowest rung with a baseline term plus an inflammation-driven term, bounded by a finite pool M_{max} and ratchet upward through rungs they exceed, at rate α (see Fig. S10).

$$\text{recruit} = (a_0 + a_1 E)\,\max\Big(0,\ 1 - \frac{N}{M_{max}}\Big), \qquad N = \sum_j M_j, \tag{6}$$

$$u_j = \alpha\,\sigma_{j+1} M_j, \quad j = 0, \ldots, K-1 \quad (\text{flux } j \to j+1), \tag{7}$$

$$\dot{M}_0 = \text{recruit} - u_0 - d_M M_0, \tag{8}$$

$$\dot{M}_j = u_{j-1} - u_j - d_M M_j, \quad 1 \le j \le K-1, \tag{9}$$

$$\dot{M}_K = u_{K-1} - d_M M_K. \tag{10}$$

A cohort therefore accumulates at the rung matching the highest drive it has experienced (its memory), and falls silent on a plateau or a falling input.

Pro-inflammatory output (the only term that differs between the three variants). The graded per-cell IL-10 amplitude factor is $\mu_j = 1 - \ell_j(1-f)$. So $\mu_j = 1$ for an unlicensed cell and $\mu_j = f$ for a fully licensed cell. The population produces inflammatory output in proportion to the common sensing drive d (Eq. 1), weighted by a per-cell output bracket B_j:

$$\dot{E} = p_E \sum_j M_j\, d\, B_j - d_E E. \tag{11}$$

The three variants differ only in B_j:

$$\textbf{(lm) licensing + memory:} \quad B_j = \sigma_j\,\mu_j, \qquad \dot{E} = p_E \sum_j M_j\, d\, \sigma_j\, \mu_j - d_E E; \tag{12}$$

bioRxiv preprint doi: https://doi.org/10.1101/2024.03.28.587272; this version posted July 9, 2026. The copyright holder for this preprint (which was not certified by peer review) is the author/funder. This article is a US Government work. It is not subject to copyright under 17 USC 105 and is also made available for use under a CC0 license.

(conc) concentration-only: $\mathrm{B_j = f}, \qquad \dot{\mathrm{E}} = \mathrm{p_E\, f} \sum_{\mathrm{j}} \mathrm{M_j\, d - d_E E};$ (13)

(none) no regulation: $\mathrm{B_j = 1}, \qquad \dot{\mathrm{E}} = \mathrm{p_E} \sum_{\mathrm{j}} \mathrm{M_j\, d - d_E E}.$ (14)

Under history-gated licensing, a cell contributes only when its escape gate is open (σ_j), and IL-10 then scales the amplitude of that firing by μ_j, to the extent the cell is licensed. This means: memory sets *which and how many* cells fire, IL-10 sets *how strongly* each firing cell outputs, and the two act multiplicatively (Section 6).

IL-10, variable R (identical in all three variants). Production is driven by the same sensing drive d (Eq. 1) and weighted by the licensing function. As reported in Fig. S2 (compare prestimulation with 0.3 nM KLA in **A** to **E**), cells begin secreting IL-10 exactly where they become able to respond to it. In addition, there is a small tonic term and a constant ambient influx:

$$\dot{\mathrm{R}} = \mathrm{p_R} \sum_{\mathrm{j}} \mathrm{M_j\, d}\,(\mathrm{r_{tonic}} + \ell_\mathrm{j}) + \mathrm{R_{amb}} - \mathrm{d_R R}. \tag{15}$$

Pathogen, variable P. Logistic growth, cleared by a saturable, inflammation-dependent term:

$$\dot{\mathrm{P}} = \mathrm{r_P\, P}\Big(1 - \frac{\mathrm{P}}{\mathrm{K_P}}\Big) - \mathrm{k_P} \frac{\mathrm{E\,P}}{\mathrm{P + h}}. \tag{16}$$

Tissue damage, variable D. Driven by inflammation and by pathogen burden, repaired at a constant rate:

$$\dot{\mathrm{D}} = \mathrm{g_E E + g_P P - d_D D}. \tag{17}$$

5. The three regulatory variants

All three variants we are exploring use identical parameters for pathogen kinetics, macrophage recruitment, and IL-10 production. The variants differ only in which cells IL-10 suppresses.

- **Licensing + memory (lm).** Only history-licensed cells ($\ell_\mathrm{j} > 0$) are IL-10-suppressible, and the escape gate σ_j lets any cell respond to a stimulus exceeding its remembered level. Newly recruited (naive) cells are refractory to ambient IL-10.
- **Concentration-only (conc).** Every cell's output is scaled by $\mathrm{f(R)}$ regardless of history: ambient IL-10 pre-mutes cells in proportion to its concentration.
- **No regulation (none).** IL-10 has no effect on output ($\mathrm{f} \equiv 1$); pure pathogen/DAMP sensing.

6. How single-cell data determine the shape of the modeled inflammatory responses

How memory (σ_j) and IL-10/licensing (ℓ_j, f) combine in the per-cell bracket $\mathrm{B_j}$ is a modeling choice that strongly affects the dynamics. We considered three functional forms and discriminated among them using single-cell TNF measurements (see Fig. 2 H, I in the main text).

Candidate forms: One potential way to implement the influence of TLR4 memory and IL-10 is an *escape-dominant additive* bracket, in which an escaping (firing) cell contributes its full output and only the non-escaping fraction is IL-10-suppressible (so a firing cell is *immune* to IL-10), with an IL-10-independent residual γ:

$$\mathrm{B_j} = \sigma_\mathrm{j} + (1 - \sigma_\mathrm{j})(1 - \gamma)\,\mu_\mathrm{j}. \tag{18}$$

bioRxiv preprint doi: https://doi.org/10.1101/2024.03.28.587272; this version posted July 9, 2026. The copyright holder for this preprint (which was not certified by peer review) is the author/funder. This article is a US Government work. It is not subject to copyright under 17 USC 105 and is also made available for use under a CC0 license.

A somewhat simpler *threshold-shift* alternative folds IL-10 into the gate itself: IL-10 raises a licensed cell's effective set-point rather than reducing its amplitude. This means that one sigmoid carries both controls, and because σ_j also drives the ratchet, IL-10 would couple to memory *formation*:

$$\theta_j^{eff} = \theta_j + \kappa\,\ell_j(1-f), \qquad B_j = \sigma_j \text{ evaluated at } \theta_j^{eff}. \tag{19}$$

The adopted *product* form (Eq. 12) instead has IL-10 multiply the output of firing cells irrespective of escape.

The three forms differ on one experimentally accessible question: *is a cell that is already firing (escaped) suppressible by IL-10?*

form	output bracket B_j	firing cell IL-10-suppressible?	verdict from data
additive	$\sigma_j + (1-\sigma_j)(1-\gamma)\mu_j$	**no** — escape overrides suppression	contradicted
threshold-shift	σ_j at θ_j^{eff}	indirectly — alters *whether* it fires, not amplitude	not the observed mode
**product (adopted)**	$\sigma_j\,\mu_j$	**yes** — amplitude scaled by μ_j	**supported**

Single-cell data. (For details, see description of data in Fig. 2 I, H.) Macrophages were primed (first challenge 0, 1, or 100 nM KLA), rested, and re-challenged (second challenge 0 or 100 nM), with isotype control or anti-IL-10R antibody on the 100 nM-primed arm; per-cell *Tnf* expression was quantified in TNF^+ cells ($n \approx 6.7$–9.1×10^3 per condition). Two axes emerge: *Memory sets the responding fraction*: with increasing first-challenge dose the TNF^+ fraction rises steeply ($\approx 8.9\% \to 38.5\% \to 81.4\%$ at second challenge 0), a fraction-encoded response. *IL-10 sets the per-cell amplitude of firing cells*: at the saturating second challenge ($\approx 95\%$ TNF^+, so the fraction is fixed), the per-cell TNF among firing cells is lower for heavily-primed cells (100/100, mean ≈ 2.56) than for lightly-primed cells (1/100, mean ≈ 3.72), and IL-10R blockade restores the heavily-primed cells to the lightly-primed level (100/100 + anti-IL-10R, mean ≈ 3.59). These are escaped, firing cells, yet IL-10 still suppresses their amplitude. This directly contradicts the additive form's escape-immunity and identifying amplitude (gain) control (Fig. S9A).

Uniform gain, not a suppressed subpopulation. Three tests on the single-cell distributions distinguish a near-uniform per-cell gain reduction from an IL-10-silenced subpopulation. (i) The suppressed distribution (100/100, isotype) is statistically unimodal (according to Hartigan dip test $p \approx 0.95$) no separated low-TNF mode. (ii) IL-10R blockade lifts the entire distribution by ≈ 1 log unit at every quantile (from $\approx +1.16$ at the lowest decile to $\approx +0.88$ at the highest; the quantile–quantile map is close to a constant offset, Fig. S9B,C), so essentially all firing cells are suppressed, not a fraction of them. (iii) Suppression barely changes the spread (standard deviation 0.70 versus 0.64 log units; a two-population mixture would broaden it), and a two-component Gaussian improves barely on a single component here.

Together these observations exclude a discrete IL-10-refractory subpopulation and show that the memory-controlled firing fraction and the IL-10-controlled per-cell amplitude are separable quantities that combine **multiplicatively**. The model therefore represents IL-10 as an amplitude factor $\mu_j = 1 - \ell_j(1-f)$ acting on firing cells, giving the product output term $B_j = \sigma_j\,\mu_j$.

bioRxiv preprint doi: https://doi.org/10.1101/2024.03.28.587272; this version posted July 9, 2026. The copyright holder for this preprint (which was not certified by peer review) is the author/funder. This article is a US Government work. It is not subject to copyright under 17 USC 105 and is also made available for use under a CC0 license.

7. Initial conditions, integration, and tissue damage metrics

The system starts from rest: $P(0) = P_0$; $M_0(0) = M_{n0}$ with all other rungs zero; $E(0) = 0$; $R(0) = R_{amb}/d_R$ (steady-state ambient tone); $D(0) = 0$; and $M2(0) = 0$. Equations are integrated to $t = 240$ h with LSODA (SciPy), relative tolerance 10^{-7}, absolute tolerance 10^{-10}. The summary metrics are the peak and final P, the final D, and the cumulative tissue insult

$$I = \int_0^T \left(g_E E + g_P P\right) dt, \tag{20}$$

decomposed into an inflammatory burden $\int_0^T g_E E\, dt$ (penalizing failure to resolve) and a pathogen burden $\int_0^T g_P P\, dt$ (penalizing failure to clear).

8. Parameters

Table 1. Default parameters are either dimensionless or rates per hour.

group	symbol = value	meaning
Pathogen	$P_0 = 0.001$	initial pathogen load
	$K_P = 1$	carrying capacity (normalization)
	$r_P = 0.9$	growth rate
	$k_P = 4$	maximal immune killing rate
	$h = 0.35$	pathogen at half-maximal killing
	$K_s = 0.05$	pathogen at half-maximal sensing
Macrophages	$M_{n0} = 0.05$	initial resident (naive) cells
	$a_0 = 0.003$	baseline recruitment
	$a_1 = 0.08$	inflammation-driven recruitment
	$d_M = 0.01$	macrophage turnover
	$M_{max} = 3$	recruitable pool bound
	$\alpha = 0.3$	memory ratchet (climbing) rate
Memory / licensing	$K = 12$	number of ladder rungs (K+1 states)
	$\theta_{min} = 0.05$	lowest non-zero set-point
	$\theta_{max} = 1.6$	highest set-point
	$\theta_{1/2} = 0.35$	licensing midpoint
	$n = 3$	licensing Hill coefficient
	$\varepsilon = 0.04$	escape softness
	$\gamma = 0.35$	(additive form only; unused in product model)
Output / IL-10	$p_E = 2$	output per activated cell
	$d_E = 1$	output decay
	$p_R = 0.8$	IL-10 production per activated cell
	$d_R = 0.15$	IL-10 decay
	$R_{amb} = 0.5$	ambient IL-10 influx
	$K_R = 0.6$	IL-10 for half-maximal suppression
	$m = 2$	IL-10 Hill coefficient
	$r_{tonic} = 0.05$	tonic IL-10 production

bioRxiv preprint doi: https://doi.org/10.1101/2024.03.28.587272; this version posted July 9, 2026. The copyright holder for this preprint (which was not certified by peer review) is the author/funder. This article is a US Government work. It is not subject to copyright under 17 USC 105 and is also made available for use under a CC0 license.

group	symbol = value	meaning
Damage	$g_E = 1$	immunopathology per unit inflammation
	$g_P = 2$	injury per unit pathogen
	$d_D = 0.1$	tissue repair rate
	$w_D = 0.6$	DAMP loop gain
	$K_D = 1$	damage at half-maximal DAMP sensing
Resolution (Sec. 10)	$k_{pol} = 0.08$	M1→M2 polarization rate
	$d_{M2} = 0.03$	M2 turnover
	$k_{clr} = 0.30$	M2-mediated damage clearance

9. Results at the default operating point

Table 2. Outcome at the default operating point (base model, product form, t = 240 h).

variant	peak P	final P	chronic D	infl. insult	path. insult	total insult
licensing + memory	0.05	→ 0 (clears)	4.5	133	1	134
concentration-only	0.97	0.96 (escape)	19.3	3	446	449
no IL-10 regulation	0.05	→ 0 (clears)	31.2	555	1	556

Interpretation. Licensing+memory is the only variant that is low on *both* failure axes (main-text Fig. 5A). Concentration-only pre-mutes naive cells through the ambient IL-10 tone, so the rising pathogen is never met and it escapes (peak $P \approx 1$); its injury is pathogen-driven. No-regulation clears the pathogen but, lacking an IL-10 brake, ignites the DAMP loop into a non-resolving sterile-inflammation state (chronic $D \approx 31$). Licensing keeps newly recruited cells refractory to ambient IL-10 so they clear the pathogen early. Then, once licensed, they become IL-10-suppressible, letting IL-10 terminate the loop. A residual low-grade sterile inflammation persists (final $D \approx 4.5$) because the DAMP plateau is sub-maximal, so cells license only partially and IL-10 suppression is incomplete. This indefinite persistence is a limitation of the base model, which has no active resolution program. The M2 extension below supplies a mechanism for resolution.

10. Extension: active resolution by M1→M2 polarization

Motivation. The base model's only brake is IL-10, and it is partial; there is no efferocytosis, no M1→M2 switch, no pro-resolving-mediator program. The bistable DAMP loop therefore has no exit and sterile inflammation persists at a fixed point indefinitely, which is unrealistic for an infection cleared within a day. Therefore, we add a minimal, mechanistic resolution arm in which the most adapted, most IL-10-suppressed macrophages convert to an M2/pro-resolving phenotype, so that resolution becomes part of the same IL-10/licensing program.

Polarization flux. A new pool M2 receives cells from the ladder. The per-rung polarization weight is the IL-10 suppression that the adapted cell at rung j experiences and is the product of its licensing and the active IL-10 fraction:

$$w_j = \ell_j\,(1-f)\ \ (\text{lm, conc}), \qquad w_j = 0\ \ (\text{none; IL-10R inert}). \tag{21}$$

bioRxiv preprint doi: https://doi.org/10.1101/2024.03.28.587272; this version posted July 9, 2026. The copyright holder for this preprint (which was not certified by peer review) is the author/funder. This article is a US Government work. It is not subject to copyright under 17 USC 105 and is also made available for use under a CC0 license.

Highly-licensed cells ($\ell_j \to 1$) under strong IL-10 ($f \to 0$) convert fastest. The conservative flux $q_j = k_{pol}\, w_j\, M_j$ is removed from each ladder equation and summed into the M2 pool, which clears the DAMP source (note additional term in equation (23)).

$$\dot{M}_j \to \dot{M}_j - q_j, \qquad q_j = k_{pol}\, \ell_j\, (1-f)\, M_j, \tag{22}$$

$$\dot{M2} = \sum_j q_j - d_{M2}\, M2, \qquad \dot{D} = g_E E + g_P P - d_D D - k_{clr}\, M2\, D. \tag{23}$$

M2 cells produce no inflammatory output. Having left the ladder, they are absent from the $\sum_j M_j$ that drives $\dot{E}$. The rates k_{pol}, d_{M2}, k_{clr} are illustrative, not fitted; $k_{pol} = 0$ recovers the base model.

Table 3. Outcome with active resolution (product form, $t = 240$ h; time courses in main-text Fig. 5B).

variant	peak P	final P	chronic D	max M2	total insult
licensing + memory	0.05	→ 0 (clears)	0.0 (resolves)	0.52	60
concentration-only	0.97	0.97 (escape)	15.7	0.08	453
no IL-10 regulation	0.05	→ 0 (clears)	31.2	0.00	556

Note on a non-physiological simplification in the model: Setting $w_j = 0$ under no-regulation makes resolution purely IL-10-driven, so that variant cannot resolve at all. Real M2 polarization also has IL-10-independent drivers (IL-4/IL-13, efferocytosis, TGF-β); a small IL-10-independent term would let no-regulation partially resolve and could reorder it against concentration-only.

Fig. S10 shows how the memory rungs of the model with the resolution extension are filled over time and then lose cells again as these enter the resolution M2-like population. The θ levels in the legend correspond to the discretized memory setpoints in Eq. 3. With the M2 extension, higher memory rungs are filled only weakly, as cells escape into the M2 state and inflammation is terminated around 200 hours. In the base model, without M2-like state, inflammation would continue beyond 200 hours (compare Fig. 5A to Fig. 5B) and cells would linger in medium-high memory rungs.

11. Global sensitivity of the licensing advantage

Setup. We quantify the robustness of the licensing+memory advantage through the two cumulative-insult differences

$$A_{conc} = I(\text{conc}) - I(\text{lm}), \qquad A_{none} = I(\text{none}) - I(\text{lm}), \tag{24}$$

the insult that licensing+memory avoids relative to concentration-only and to no-regulation; the per-draw decomposition into pathogen and inflammatory burden is shown in main-text Fig. 5C. We draw Latin-hypercube samples ($N = 300$) with every non-structural parameter uniform on $[0.5, 1.5]\times$ its default (structural quantities K_P, K, the ladder bounds, P_0, M_{n0}, the Hill exponent n, and the unused γ remain fixed), and rank each parameter's monotone influence by partial rank correlation (PRCC).

Base model. Licensing+memory has the lowest cumulative insult in 80% of draws (beating concentration-only in 83%, no-regulation in 97%; median $A_{conc} = 306$, $A_{none} = 333$). The two advantages behave oppositely, and the PRCC explain why through a sign flip (Fig. S11): the inflammation-cost parameters

bioRxiv preprint doi: https://doi.org/10.1101/2024.03.28.587272; this version posted July 9, 2026. The copyright holder for this preprint (which was not certified by peer review) is the author/funder. This article is a US Government work. It is not subject to copyright under 17 USC 105 and is also made available for use under a CC0 license.

(output per cell p_E, DAMP gain w_D, immunopathology g_E, the macrophage ceiling M_{max}) lower A_{conc} but raise A_{none}. Making inflammation expensive penalizes licensing+memory's own (controlled) response relative to concentration-only's near-silence, yet simultaneously makes the IL-10 brake more valuable relative to the unbraked case. The pathogen-danger parameter g_P acts almost only on A_{conc}, punishing concentration-only's escape. This is the well-known resistance-versus-tolerance trade-off, recovered mechanistically: regulated resistance (licensing+memory) beats indiscriminate suppression when the pathogen is dangerous (g_P high) and inflammation is containable (d_E high, $p_E/w_D/g_E$ low); when inflammation is extremely costly and the pathogen benign, tolerating it can win.

With M1→M2 conversion. Repeating the analysis on the resolution model increases the advantage of the licensing+memory variant: licensing+memory is now best in 87% of samples (versus 80%), beats concentration-only in 91% (versus 83%), and the no-regulation advantage holds at 96%; the median advantages rise (A_{conc}: 306 $\to$ 318, A_{none}: 333 $\to$ 387). The mechanism is visible in the PRCC: the inflammation-cost parameters that erode A_{conc} in the base model have weaker influence once M2 is present, because licensing+memory can now resolve the inflammation it generates, so the regime in which tolerance beats resistance contracts. The per-cell output rate p_E enters the two advantages with opposite sign, narrowing the clearance gap against concentration-only but widening the resolution gap against no-regulation.

Caveats. The analysis uses $\pm 50\%$ uniform ranges and PRCC, which assumes monotone parameter effects; a few affinities (e.g. K_R) may be non-monotone and have their influence understated. The M2 rates are illustrative; their own influence is modest, so the robustness gain is a structural consequence of possessing a resolution arm, not a product of tuning unfitted parameters. Parameters were not refitted to the product-form operating point, so the qualitative ordering and the licensing advantage are robust while absolute magnitudes are not determined by the data or the model variants.

12. A parameter set without runaway pathogen in the concentration-only case

Concentration-only regulation fails in most of the parameter space by runaway pathogen escape. However, when the pathogen is intrinsically self-limiting (lower carrying capacity K_P) and IL-10 suppression is weaker (higher K_R), the muted response is still sufficient to hold the pathogen at a sub-maximal plateau rather than losing control entirely (Fig. S13; final P ≈0.51, about 73% of ceiling, at $K_P = 0.7$, $K_R = 1.25$).
This regime is rather special: across a $\pm 50\%$ sweep of the non-structural parameters, concentration-only escapes outright (>85% of ceiling) in $\approx$ 89% of draws, controls only partially in $\approx$ 7%, and clears in $\approx$ 4%. Fig. S14 shows where this partial-control regime is located in the (K_R, K_P) plane: it is reached by simultaneously weakening suppression and lowering the ceiling from the default operating point (final $P : 0.96 \to 0.51$). The plot also shows that history-gated licensing still incurs the lower cumulative insult there. Partial control therefore softens the concentration-only failure without reversing the ordering: even where blanket suppression avoids runaway pathogen, it trades escape for a persistent sub-maximal pathogen burden and the accompanying damage. As a result, it pays more in total tissue insult than history-gated licensing. The licensing advantage is thus not an artifact of comparing it to an always catastrophically failing scenario with runaway pathogen. It holds whether concentration-only fails by escape or merely by excess injury under partial control.

bioRxiv preprint doi: https://doi.org/10.1101/2024.03.28.587272; this version posted July 9, 2026. The copyright holder for this preprint (which was not certified by peer review) is the author/funder. This article is a US Government work. It is not subject to copyright under 17 USC 105 and is also made available for use under a CC0 license.

Supplementary Figures

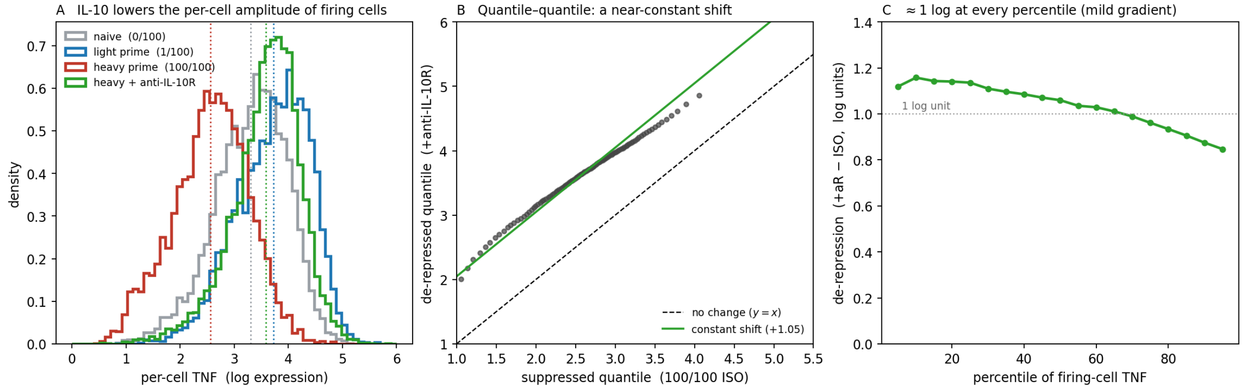

Fig. S9. Single-cell TNF in TNF$^+$ cells discriminates gain from subpopulation control (data shown in Fig. 2 H, I) (A) Per-cell TNF distributions of firing cells: IL-10 slides the heavily-primed distribution (100/100, mean 2.56) downward relative to the lightly-primed one (1/100, mean 3.72), and anti-IL-10R blockade restores it (mean 3.59); naive cells (0/100, mean 3.30) are shown for reference. **(B)** Quantile–quantile comparison of the suppressed (100/100) and de-repressed (+anti-IL-10R) distributions lies close to a constant offset ($\approx +1.05$ log units; uniform gain), not peeling away at the lower quantiles as a suppressed subpopulation would. **(C)** The de-repression is $\approx$ 1 log unit at every percentile, with only a mild gradient (low expressers suppressed slightly more).

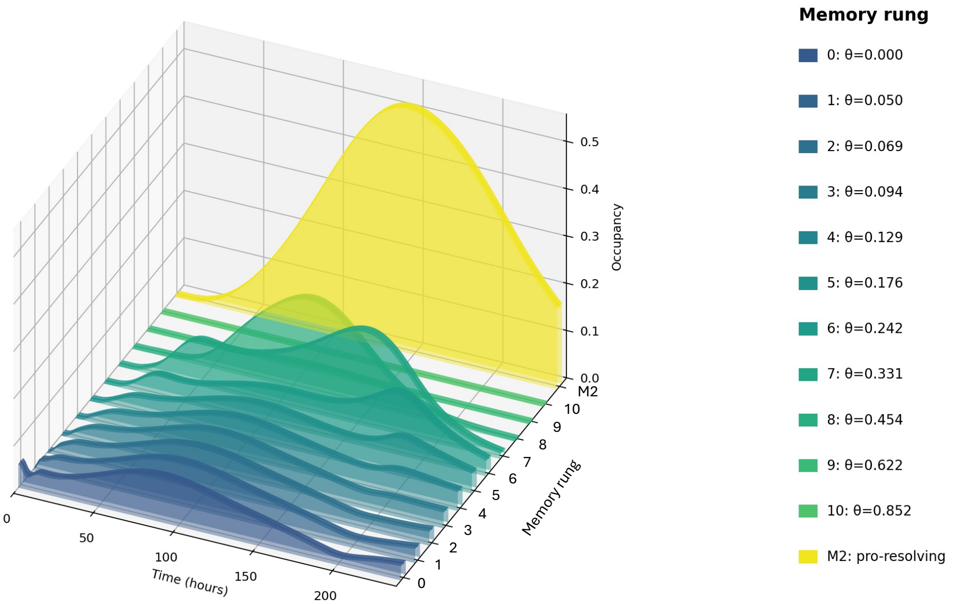

Fig. S10. Dynamics of memory rung occupation over time (resolution model). Dynamics of memory rung occupation over time for a simulation of the resolution model. The values in the legend correspond to the memory set points in (Eq. 3). Note that, in this extension with M2-like cells, the higher memory rungs are filled only weakly since the cells escape into the M2 state.

bioRxiv preprint doi: https://doi.org/10.1101/2024.03.28.587272; this version posted July 9, 2026. The copyright holder for this preprint (which was not certified by peer review) is the author/funder. This article is a US Government work. It is not subject to copyright under 17 USC 105 and is also made available for use under a CC0 license.

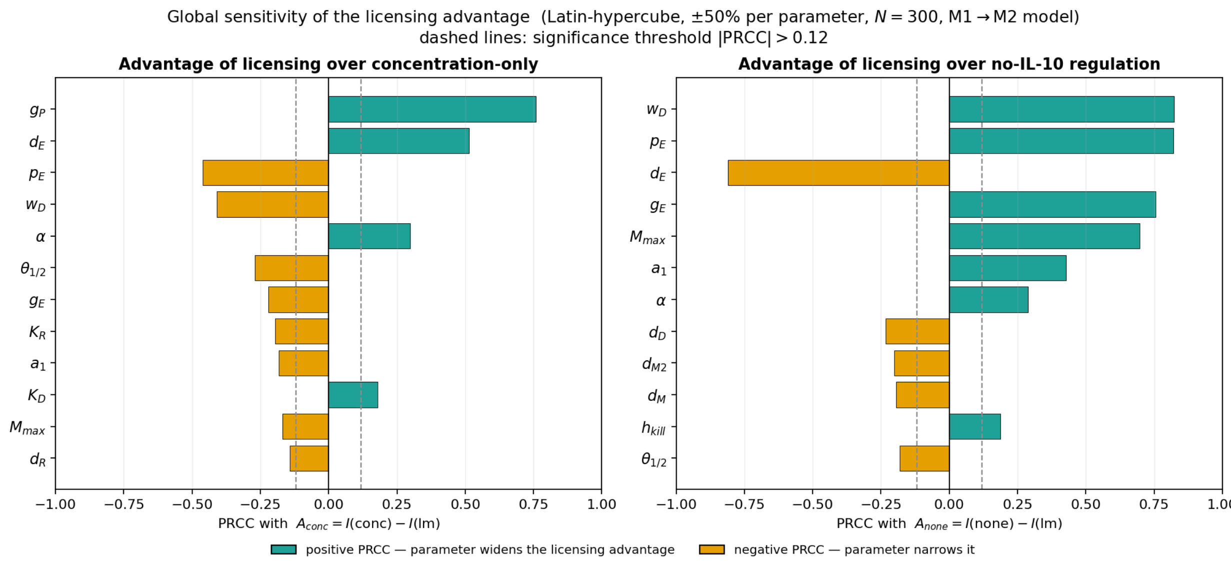

Fig. S11. Global sensitivity of the licensing advantage (resolution model). PRCC between each parameter and the advantage of history-gated licensing over **(left)** concentration-only, A_{conc}, and **(right)** no regulation, A_{none} (twelve largest-magnitude parameters per panel; teal = widens the advantage, orange = narrows it; dashed lines, $|\text{PRCC}| > 0.12$). The advantage over concentration-only is governed mainly by the pathogen-cost parameter g_P and narrowed by weaker IL-10 suppression (larger K_R); the advantage over no-regulation by inflammation-cost parameters (w_D, p_E, g_E, M_{max}). The output rate p_E changes sign between the panels, illustrating the resistance/tolerance trade-off in a single parameter.

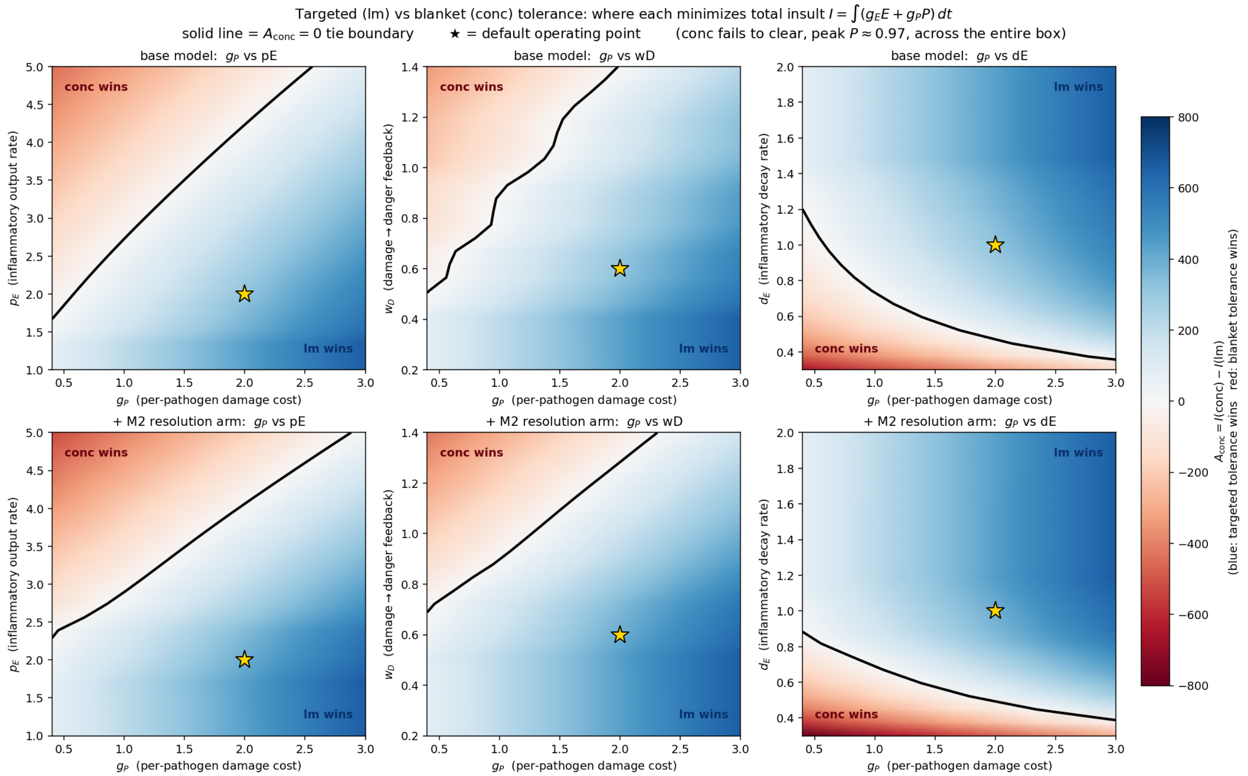

Fig. S12. Boundary maps of the licensing advantage. A_{conc} on a 24 × 24 grid (blue: licensing lower insult;

bioRxiv preprint doi: https://doi.org/10.1101/2024.03.28.587272; this version posted July 9, 2026. The copyright holder for this preprint (which was not certified by peer review) is the author/funder. This article is a US Government work. It is not subject to copyright under 17 USC 105 and is also made available for use under a CC0 license.

red: concentration-only lower; solid line: $A_{conc} = 0$ tie; gold star: default operating point). Columns vary g_P against the tolerance parameters p_E (left), w_D (center), d_E (right); top row base model, bottom row with resolution. Licensing gives the lower insult across most of each panel (67–83%), and concentration-only never clears the pathogen anywhere in the box (peak $P \approx 0.97$): its "wins" are pure tolerance wins. The resolution arm leaves the tie boundary essentially unchanged. Note that the 'wavy' structure in the upper middle panel is an artifact, but not of limited sampling, but stemming from the discrete nature of the memory rungs.

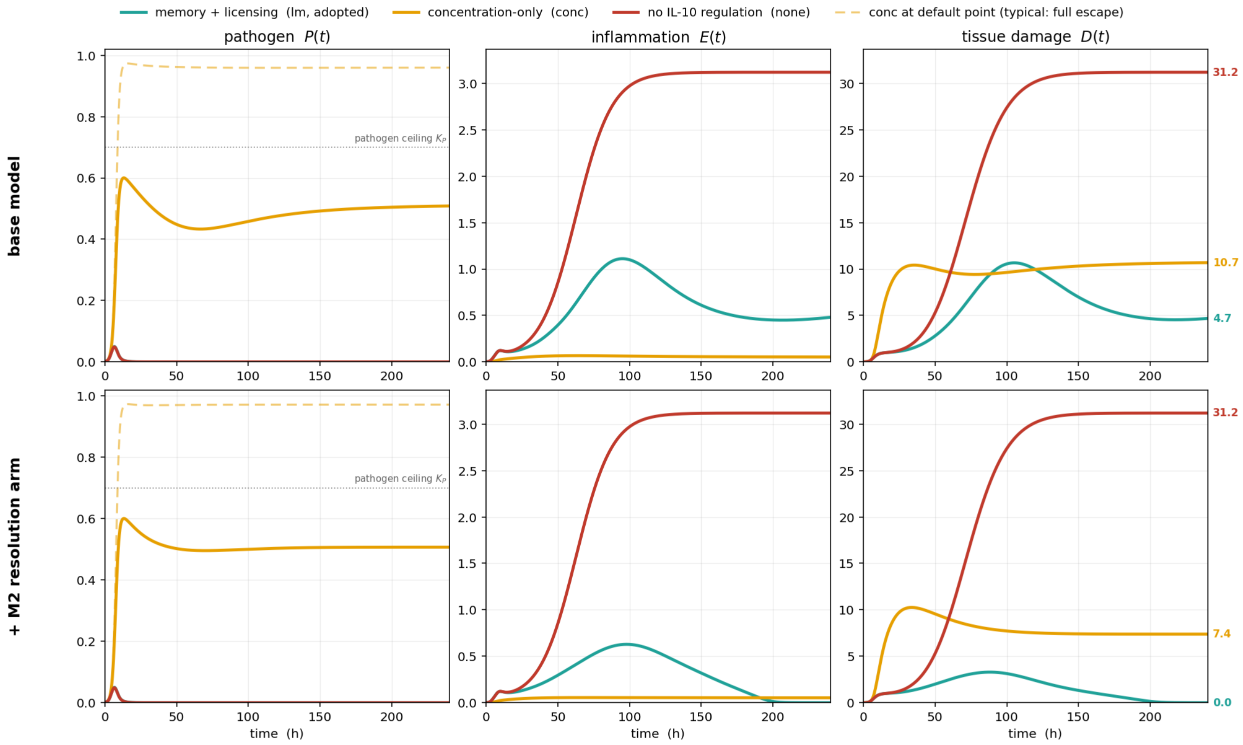

Fig. S13. A non-generic operating point where concentration-only fails only partially. Time courses at $K_P = 0.7$, $K_R = 1.25$ (self-limiting pathogen, modestly weaker IL-10); base model (top) and with resolution (bottom); colors as in Fig. 5A, with the faint dashed curve showing concentration-only at the default point (typical full escape). Here concentration-only holds the pathogen at ≈ 0.51 ($\approx 73\%$ of ceiling) rather than escaping, yet still incurs the greater damage. This regime is non-generic: across a $\pm 50\%$ sweep, concentration-only fully escapes ($> 85\%$ of ceiling) in $\approx 89\%$ of draws, partially controls in $\approx 7\%$, and clears in $\approx 4\%$.

bioRxiv preprint doi: https://doi.org/10.1101/2024.03.28.587272; this version posted July 9, 2026. The copyright holder for this preprint (which was not certified by peer review) is the author/funder. This article is a US Government work. It is not subject to copyright under 17 USC 105 and is also made available for use under a CC0 license.

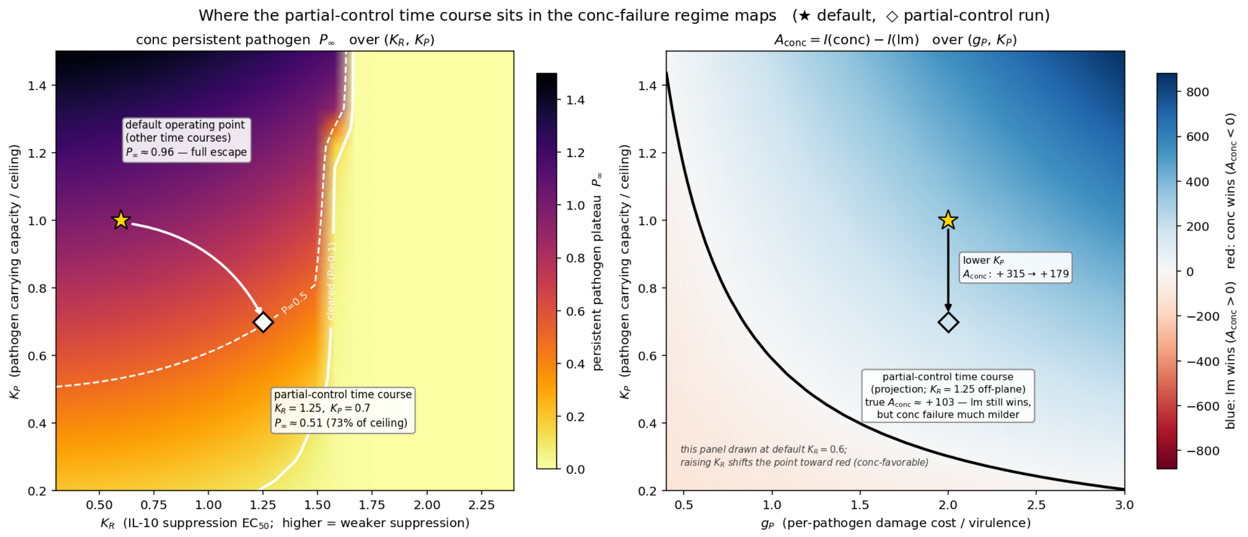

Fig. S14. Location of the partial-control regime. (Left) Persistent pathogen plateau P_∞ under concentration-only over (K_R, K_P): the default point (star) escapes fully ($P_\infty \approx 0.96$); weakening suppression and lowering the ceiling reaches the partial-control point (diamond, $P_\infty \approx 0.51$). **(Right)** A_{conc} over (g_P, K_P); because the panel is drawn at the default K_R, the partial-control point appears as an off-plane projection. Licensing still gives the lower insult there ($A_{conc} \approx +103$): the partial-control regime makes the concentration-only failure milder without making it the better strategy.
